# Synthesis of 3-Aryl-3-(Furan-2-yl)Propanoic Acid Derivatives, and Study of Their Antimicrobial Activity

**DOI:** 10.3390/molecules27144612

**Published:** 2022-07-19

**Authors:** Mikhail V. Kalyaev, Dmitry S. Ryabukhin, Marina A. Borisova, Alexander Yu. Ivanov, Irina A. Boyarskaya, Kristina E. Borovkova, Lia R. Nikiforova, Julia V. Salmova, Nikolay V. Ul’yanovskii, Dmitry S. Kosyakov, Aleksander V. Vasilyev

**Affiliations:** 1 Department of Chemistry, Saint Petersburg State Forest Technical University, Institutsky Per., 5, 194021 Saint Petersburg, Russia; kalyaev2017@yandex.ru (M.V.K.); rdms@bk.ru (D.S.R.); marina96.00@mail.ru (M.A.B.); 2 All-Russia Research Institute for Food Additives—Branch of V.M. Gorbatov Federal Research Center for Food Systems of RAS, Liteyniy Pr., 55, 191014 Saint Petersburg, Russia; 3 Center for Magnetic Resonance, Research Park, Saint Petersburg State University, Universitetskiy Pr., 26, 198504 Saint Petersburg, Russia; alexander.ivanov@spbu.ru; 4 Department of Organic Chemistry, Institute of Chemistry, Saint Petersburg State University, Universitetskaya Nab., 7/9, 199034 Saint Petersburg, Russia; iralbo@yahoo.com; 5 Research and Manufacturing Company «Home of Pharmacy», Zavodskaya St., 3-245, Kuz’molovskiy Settlement, 188663 Saint Petersburg, Russia; borovkova.ke@doclinika.ru (K.E.B.); nikiforova.lr@doclinika.ru (L.R.N.); salmova.uv@doclinika.ru (J.V.S.); 6 Core Facility Center “Arktika”, Northern (Arctic) Federal University, Nab. Severnoy Dviny, 17, 163002 Arkhangelsk, Russia; uluanovskii_n@mail.ru (N.V.U.); kosyakov@mail.ru (D.S.K.)

**Keywords:** furans, Friedel–Crafts reaction, carbocations, superelectrophilic activation, antibacterial activity

## Abstract

Reactions of 3-(furan-2-yl)propenoic acids and their esters with arenes in Brønsted superacid TfOH affords products of hydroarylation of the carbon–carbon double bond, 3-aryl-3-(furan-2-yl)propenoic acid derivatives. According to NMR and DFT studies, the corresponding O,C-diprotonated forms of the starting furan acids and esters should be reactive electrophilic species in these transformations. Starting compounds and their hydroarylation products, at a concentration of 64 µg/mL, demonstrate good antimicrobial activity against yeast-like fungi *Candida albicans*. Apart from that, these compounds suppress *Escherichia coli* and *Staphylococcus aureus*.

## 1. Introduction

Nowadays, biomass-derived furans such as furfural and 5-hydroxymethylfurfural (5-HMF) are paid great attention to, and they are considered platform chemicals [1,2,3,4]. These compounds and their derivatives are widely used for the synthesis of many fine chemicals, pharmaceuticals, polymers, resins, solvents, adhesives, fungicides, paints, antifreezes, fuels, and others [5,6,7,8,9,10]. It should be especially emphasized that furans and tetrahydrofurans are well-known drugs that are actively used for medicine and veterinary practices, for instance, Nitrofural, Nitrofurantoin, and Lasalocid (antibacterial agents); Monensin and Nigericin (polyether antibiotics); Nifurtimox (antiparasitic drug); Naftidrofuryl (peripheral vasodilator drug); Ranitidine (histamine H2 antagonist); Darunavir (HIV protease inhibitor); and Ribavirin, Taribavirin, Remdesivir, and Molnupiravir (drugs to treat virus infections, including COVID-19) [11,12]. Thus, the further development of synthesis of novel compounds from furfural and 5-HMF is an actual goal not only for organic chemistry but also for medicine, material science, and for other fields.

Based on our research on superelectrophilic activation in organic synthesis [13,14] and our investigation in furan chemistry [15,16], we undertook this study on the synthesis of novel compounds from furfural and 5-HMF. The main goals of this work were the synthesis of 3-(furan-2-yl)propenoic acids and their esters, investigation of their reactions of with arenes under the activation by strong Brønsted (triflic acid TfOH (CF_3_SO_3_H)) and Lewis (AlCl_3_ and AlBr_3_) acids, study on the reaction cationic intermediates by NMR and DFT calculations, and study on the biological activity of the reaction products.

## 2. Results and Discussion

**Figure 1:**
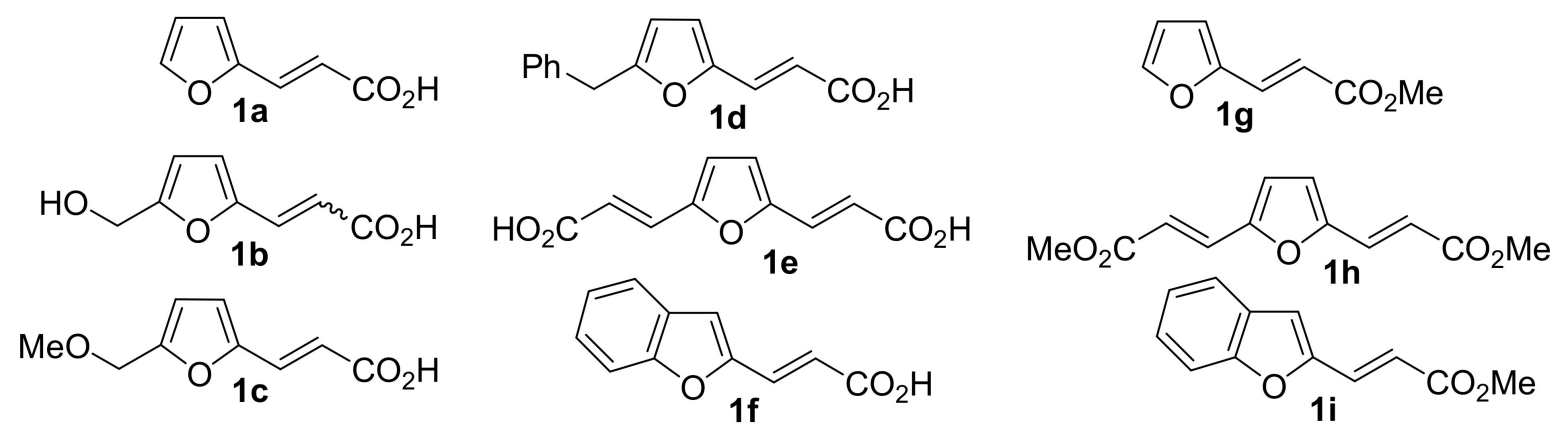
Starting 3-(furan-2-yl)propenoic acid derivatives **1a**–**i** used in this study.

Starting 3-(furan-2-yl)propenoic acids **1a**–**f** were synthesized by condensation of the corresponding furan-2-carbaldehydes (furfural (for **1a**), 5-HMF, and its derivatives (for **1b–d**), 2,5-DFF (for **1e**), and benzofurfural (for **1f**)) with malonic acid, methyl esters **1g**, **1h,** and **1i** were obtained by esterification of acids **1a**, **1e,** and **1f,** respectively (Figure 1). Acid **1b** was obtained as a mixture of E-,Z-isomers. Other compounds of **1** were isolated as E-isomers. See the preparation and characterization of compounds **1a**–**i** in the Experimental part and SI.

First, reactions of acid **1a** with benzene under the actions of various Brønsted or Lewis acids have been investigated. It was found that compound **2a** as product of hydrophenylation of the carbon–carbon double bond has been formed (Table 1). The highest yield of **2a** (65%) has been achieved under the use of AlCl_3_ at room temperature for 1 h (entry 4). Prolongation of the reaction time until 4 h leads to a decrease of the yield of **2a** to 47% (entry 5). Reaction with AlBr_3_ gives a comparable yield of the target compound (entry 7). Acidity of trifluoroacetic acid has been found to be not enough for electrophilic activation of starting substrate **1a** (entry 1). On the other hand, H_2_SO_4_ and FeBr_3_ lead to the formation of mixtures of oligomeric materials (entries 2 and 3). Yields of **2a** for reactions in TfOH under different conditions are moderate, 22–33%, at the complete conversion of starting acid **1a** (entries 7–10).

**Scheme 1:**
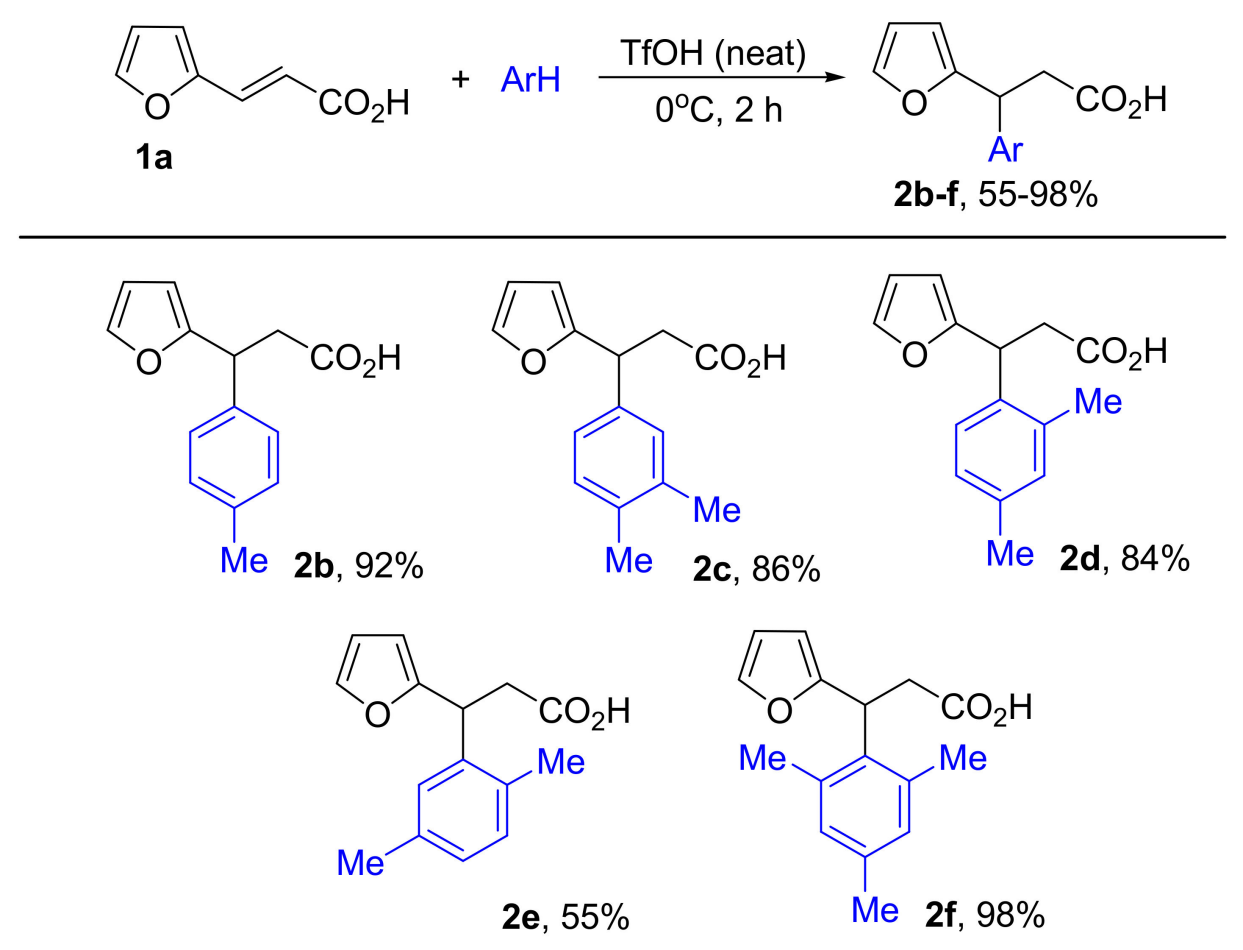
Reactions of acid **1a** with methylated arenes in TfOH leading to compounds **2b–f**.

Then, reactions of acid **1a** with other arenes have been studied. Reactions with methylated arenes (*o-*, *m-*, *p*-xylenes, mesitylene, and durene) in TfOH at 0 °C for 2 h afford the corresponding products of hydroarylation **2b**–**f** in good yields of 55–98% (Scheme 1). Contrary to the reaction with benzene (Table 1, entry 4), AlCl_3_-promoted transformations of **1a** with these methylated arenes lead mainly to oligomeric compounds with the formation of small amounts of target compounds **2b**–**h**. It should be noted that the reaction of **1a** with electron-donating arenes, anisole (methoxybenzene), veratrole (1,2-dimethoxybenzene), or electron-poor 1,2-dichlorobenzene under the action of both TfOH and AlCl_3_ give oligomeric materials.

Reaction of acids **1b**–**d**, obtained from 5-HMF, with benzene under the action of TfOH or AlCl_3_ furnishes compound **2g** (Table 2). Apart from hydrophenylation of the carbon–carbon double bond, the additional alkylation of benzene by the CH_2_OH or CH_2_oMe groups takes place for acids **1b**,**c**. Yields of target compound **2g** are comparable for a reaction with TfOH or AlCl_3_ (compare pairs of entries: 2 and 6, 7 and 9, and 10 and 12).

**Scheme 2:**
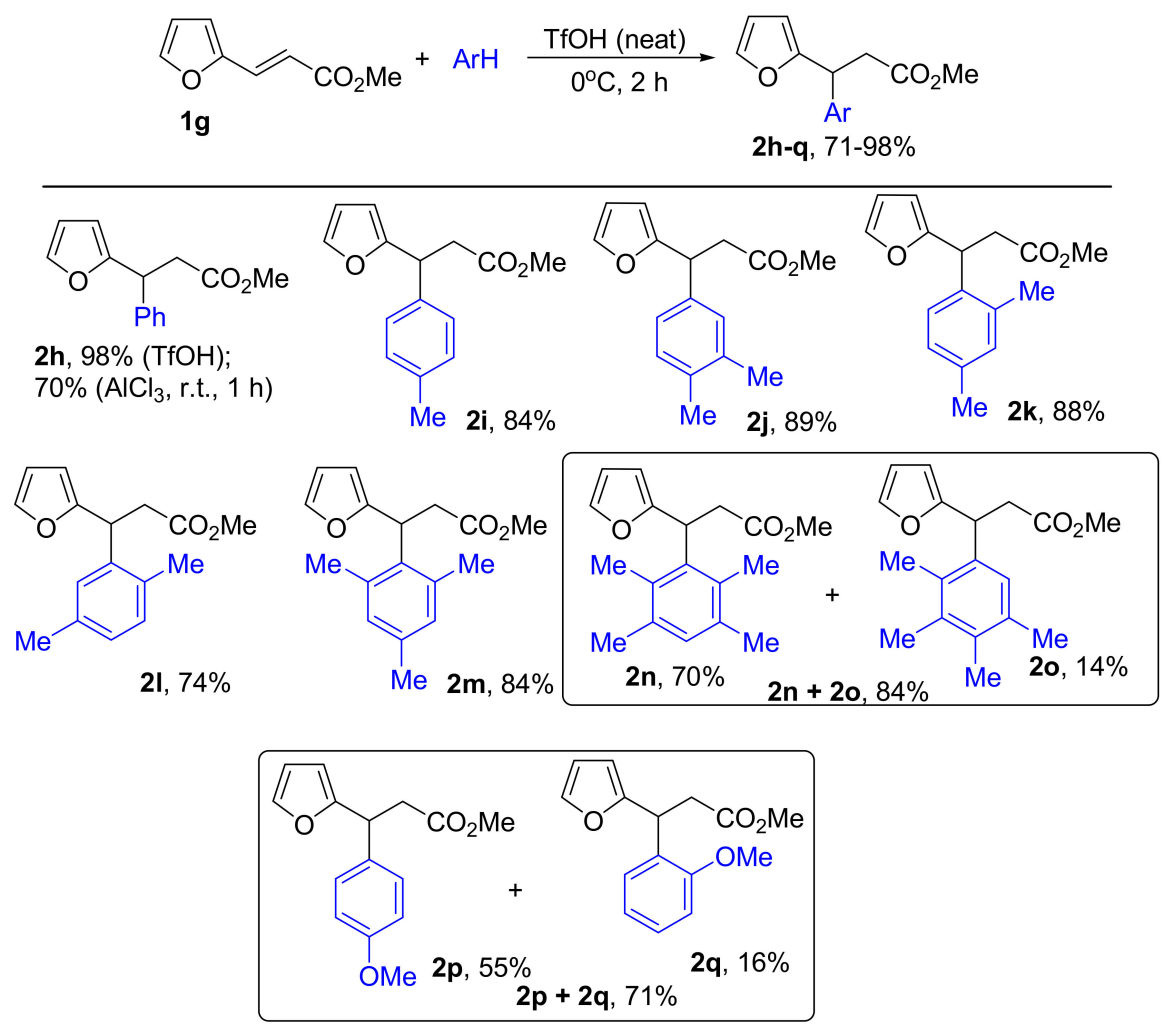
Reactions of ester **1g** with arenes in TfOH leading to compounds **2h–q**.

Hydroarylation of ester **1g** by different arenes in TfOH at 0 °C for 2 h is shown in Scheme 2. These reactions lead to the formation of compounds **2h–q** in good yields. In general, yield of esters **2** are higher with the use of TfOH rather than AlCl_3_ (see yields for **2j** in Scheme 2). Reactions with anisole and durene give mixtures of isomers **2n** and **2o** and **2p** and **2q,** respectively.

**Scheme 3:**
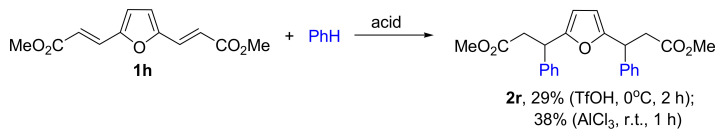
Reactions of ester **1h** with benzene under the action of TfOH or AlCl_3_ leading to compound **2r**.

In the same transformation, diester **1h** with benzene under the action of TfOH or AlCl_3_ gives a product of double hydrophenylation **2r** as an equimolar mixture of diastereomers in a moderate yield (Scheme 3).

Diacid **1e**, and benzofuran derivatives **1f** and **1i** in reactions with benzene and other arenes under the action of both TfOH and AlCl_3_ afford complex mixtures of oligomeric materials.

As it has been mentioned above, acids **1a** (Table 1, entry 2) and **1b** (Table 2, entry 1) in reactions with benzene in H_2_SO_4_ at room temperature for 1 h gave oligomeric compounds. According to the HPLC-HRMS analysis (Figure S51), the latter were represented by a number (about 20–25 chromatographic peaks for each parent compound) of dimers–hexamers with molecular weights in the range of 200–700 Da, while the most intense signals belonged to trimeric and tetrameric compounds (Table 3).

A specific feature of these products is a surprisingly large number of oxygen atoms in their elemental compositions and RDB (ring and double bond equivalent or unsaturation degree) values lower than expected. Tandem mass spectra of the corresponding precursor ions presented in the Supplementary Materials demonstrate the loss of 1–4 (depending on compound) water molecules (−18.0106 Da), which is evidence of the presence of aliphatic hydroxyl groups in their structures. This makes it possible to assume that, under applied reaction conditions, the hydration of carbon–carbon double bonds in the side chain and furane ring occurs in addition to the hydrophenylation described above for other reaction systems and confirmed by the presence of the tropylium ion [C_7_H_7_]^+^ signal at *m*/*z* 91.0565. Moreover, in most cases, the same double bond simultaneously undergoes phenylation and hydroxylation. The further oligomerization proceeds through the addition of **1a** or its hydrated derivatives and, thus, the formation of ether or ester bonds, in some cases along with the side processes of decarboxylation (the latter also can proceed during ESI in the ion source). In the case of starting compound **1b,** the same patterns were observed; however, the structures of the oligomers typically included two phenyl moieties. The plausible structural formulas and tandem mass spectra for all products listed in Table 3 are presented in the Supplementary Materials (Figures S52–S59). These oligomers are humin-like compounds similar to those obtained from furan derivatives in acidic media [17,18].

To investigate the reaction mechanism, we carried out a NMR study on the protonation of compounds **1** in TfOH. NMR monitoring of the solutions of compounds **1a–d**,**f**,**g**,**i**, having only one conjugated enone system, has shown that these compounds are rapidly transformed into oligomeric materials in TfOH. That reveals a high electrophilic reactivity of intermediate cations. Contrary to that, diacid **1e** and diester **1h**, having two conjugated enone systems, give stable solutions of O,O-diprotonated species **Ae** and **Ah,** respectively (see ^13^C NMR data in Table 4). Comparison of the chemical shifts of carbon atoms in starting compounds **1e** and **1h** and their protonated forms **Ae** and **Ah** show large down field shifts of the corresponding signals in cations. A positive charge is substantially delocalized from the carbonyl group into the carbon–carbon double bond and furan ring. Thus, differences in chemical shifts *Δδ* for carbons C^3^ and C^5^ are around 7 and 27 ppm, correspondingly (Table 4).

Then, we did DFT calculations of intermediate cations **Aa**-**Ch** derived under the protonation of 3-(furan-2-yl)propenoic acid derivatives **1a**,**g**, diacid **1e,** and diester **1h** to estimate the electrophilic properties and reactivity of these species (Table 5). Gibbs energies ∆G_298_ of protonation reactions **1**→**A**→**B**→**C**; electronic and orbital characteristics (charge distribution, HOMO/LUMO energies, contribution of atomic orbitals into LUMO, and global electrophilicity index ω [19]) of cations **Aa**-**Ch** have been calculated.

Big negative values of Gibbs energies of the protonation of acid **1a** and ester **1g,** leading to the corresponding O-protonated forms **Aa** and **Ag,** reveal that these reactions are thermodynamically favorable (entries 1 and 3). Despite positive values of Gibbs energies for the second protonation of cations, **Aa** and **Ag** with the formation of O,C-diprotonated species **Ba** and **Bg** (entries 2 and 4), for two-step processes **1a**→**Aa**→**Ba** and **1g**→**Ag**→**Bg**, the ∆G values are negative. That indicates the possibility of the formation of dications **Ba** and **Bg** in Brønsted superacids. Thus, one may propose that dications **B**, generated from 3-(furan-2-yl)propenoic acid derivatives **1a–d**,**f**,**g**, are key reactive electrophilic intermediates. Calculations of electrophilic properties of species **Ba** and **Bg** show that they have values of the electrophilicity index ω 5.2 and 5.3 eV, correspondingly. These species bear small positive charges (0.02 e) on the reactive center C^3^. However, this carbon gives a big contribution to LUMO (~27–30%) (entries 2 and 4). This points out that the reactivity of carbon C^3^ in dications **B** is explained by orbital factors rather than electronic ones.

Diprotonation of carbonyl oxygens in diacid **1e** and diester **1h,** leading to O,O-diprotonated species **Ae** and **Ah,** is thermodynamically favorable, and the corresponding ∆G values are −117.3 and −54.6 kJ/mol (entries 5 and 8). The next protonation steps **Ae**→**Be**→**Ce** and **Ah**→**Bh**→**Ch** are much less thermodynamically favorable. It should be emphasized that dications **Ae** and **Ah** have electrophilicity indexes ω 5.2 and 5.4 eV, and these values are very close to indexes for dications **Ba**, **Bg** derived from compounds **1a** and **1g** (vide supra). Taking into account NMR data on the formation of solutions of dications, **Ae** and **Ah** in TfOH (Table 4), one may propose that these species may be reactive intermediates in reactions with aromatic nucleophiles. Electronic properties of dications **Ae** and **Ah** show that the reactivity of carbon C^3^ is mainly explained as orbital factors, since this carbon possesses a negative charge (−0.19 e), but it gives an 11.4–11.6% contribution into LUMO (entries 5 and 8).

**Scheme 4:**
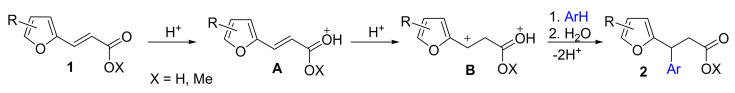
Plausible reaction mechanism of the reaction of 3-(furan-2-yl)propenoic acid derivatives **1** (except diester **1h**) with arene in Brønsted superacid TfOH, leading to compounds **2**.

Based on the data obtained on the reactions of compounds **1** with arenes (Table 1 and Table 2 and Scheme 1, Scheme 2 and Scheme 3), NMR (Table 3) and DFT (Table 4) studies of intermediate cations, one may propose a plausible reaction mechanism of the reaction of compounds **1**, except diester **1h** (vide infra), with arenes leading to products of hydroarylation **2** (Scheme 4). The first protonation of substrates **1** in Brønsted superacid TfOH occurs onto carboxyl oxygen forming O-protonated species **A**. Then, the protonation of the carbon–carbon double bond may give O,C-diprotonated species **B**. In principle, both species **A** and **B** may take part in electrophilic aromatic substitution with arenes. However, taking into account a strong electron-donating character of furan substituent, the second protonation of the conjugated C=C bond may proceed, leading to dications **B**. Moreover, the formation of such O,C-diprotonated species from various conjugated enones, such as butenones [20], indenones [21], cinnamic acids, and their esters and amides [22,23,24,25], was proven by NMR in Brønsted superacids. These dications are key reactive intermediates in various processes of electrophilic aromatic substitution [20,21,22,23,24,25,26,27,28]. Thus, it is the most probable that dications **B** lie in the reaction pathway from compounds **1** to **2**. Reactions under the action of AlCl_3_ proceed in the same manner when the electrophilic activation of substrate **1** is achieved by coordination of this strong Lewis acid onto carbonyl oxygen of the carbon–carbon double bond, leading to reactive intermediate species.

**Scheme 5:**
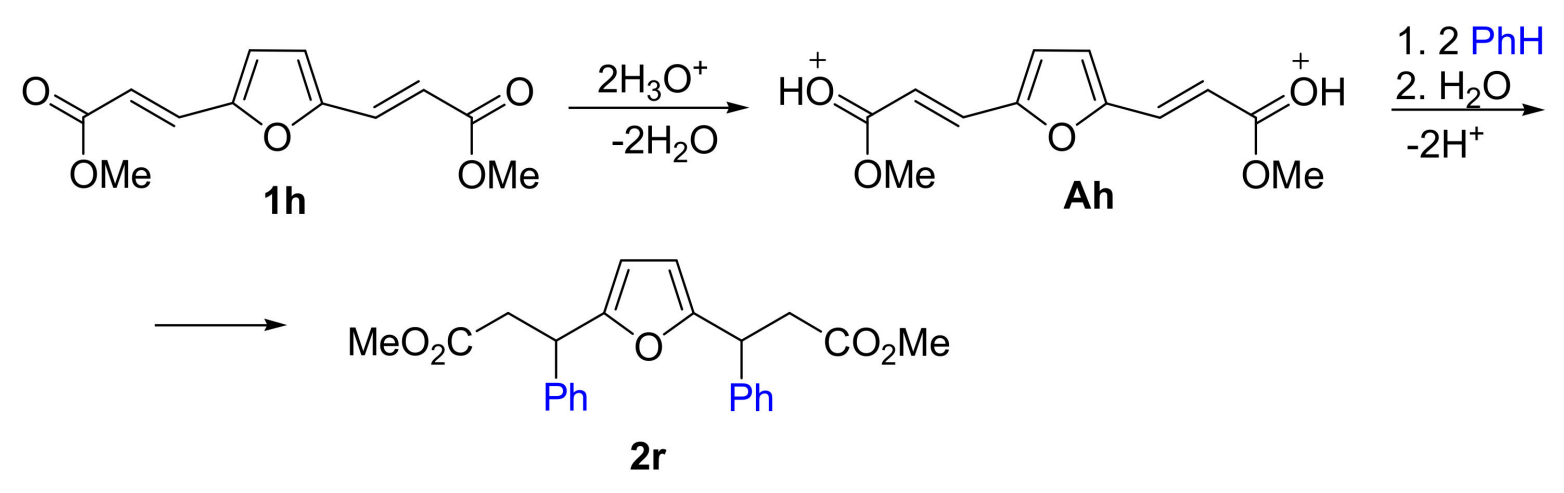
Plausible reaction mechanism of the reaction of diester **1h** with benzene in TfOH, leading to compounds **2t**.

In the case of diester **1h**, the reaction in TfOH proceeds through the intermediate formation of O,O-diprotonated species **Ah**, which reacts with benzene, affording bis-hydrophenylation product **2r** (Scheme 5).

At the final stage of this study, the antimicrobial activity of the starting furan derivatives **1** and products of their hydroarylation **2** were investigated relative to the bacteria *Escherichia coli* ATCC 25922 and *Staphylococcus aureus* ATCC 29213 and to yeast-like fungi *Candida albicans* (ATCC 10231) (see details in SI). It was found that all the compounds **1** and **2** inhibited the growth of yeast-like fungi *Candida albicans* at a concentration of 64 µg/mL. Concerning to the *S. aureus* strain, a minimum inhibitory concentration (MIC) was 128 µg/mL for most of the tested objects. The best result demonstrated acid **2d**, which suppressed the growth of microorganisms at a concentration of 64 µg/mL. However, the tested compounds **2i**, **2m**, and **2r** did not show antimicrobial activity at the specified concentrations. For most of the tested compounds, the MIC against *E. coli* ranged between 64 and 128 µg/mL.

Disinfectants, such as benzalkonium chloride and cetylpyridinium chloride, have similar MIC values for *E. coli* and *S. aures* bacterial strains. Wu et al. investigated the antimicrobial activity of quaternary ammonium compounds (QAC). According to their results, for *E. coli,* the MIC values ranged from ≤8 to 128 μg/mL benzalkonium chloride (MIC_90_ = 128 μg/mL) and ≤32 and 256 μg/mL cetylpyridinium chloride (MIC_90_ = 128 μg/mL). For *S. aureus,* isolates MIC of QAC varied from ≤2 to 128 μg/mL of benzalkonium chloride (MIC_90_ = 128 μg/mL) and from ≤4 to 256 μg/mL of cetylpyridinium chloride (MIC_90_ = 256 μg/mL) [29]. Zhang et al. established the susceptibility to cetylperidinium chloride and benzalkonium chloride of 255 *E. coli* retail meat isolates. The MIC for cetylperidinium chloride against *E. coli* ranged from 8 to 512 µg/mL and from 16 to 1024 µg/mL to benzalkonium chloride [30]. Guskova et al. established, that hydroxymethylquinoxaline dioxide (dioxidine) has antibacterial and antifungal activity ranging between 64 and 512 µg/mL against *S. aureus* strains, MIC = 16 µg/mL against the *E.coli* reference strain and MIC = 1024 µg/mL for yeast-like fungi *C. albicans* [31].

## 3. Conclusions

A novel method of synthesis of 3-aryl-3-(furan-2-yl)propenoic acid derivatives has been developed on the basis of hydroarylation of the carbon–carbon double bond of 3-(furan-2-yl)propenoic acids and their esters by arenes under superelectrophilic activation conditions in neat triflic acid TfOH. The obtained furans have demonstrated a high level of antimicrobial activity against yeast-like fungi *Candida albicans*, and they also can inhibit *Escherichia coli* and *Staphylococcus aureus*.

## 4. Experimental Part

### 4.1. General Information

The NMR spectra of solutions of compounds in CDCl_3_ were recorded on Bruker AM-500 spectrometer (Bruker Company, Germany) at 25 °C at 500 and 125 MHz for ^1^H and ^13^C NMR spectra, respectively. The residual proton-solvent peaks CDCl_3_ (*δ* 7.26 ppm), DMSO-*d*_6_ (*δ* 2.50 ppm), CD_3_OD (*δ* 3.31 ppm), (CD_3_)_2_CO (*δ* 2.05 ppm) for ^1^H NMR spectra, and the carbon signals of CDCl_3_ (*δ* 77.0 ppm), DMSO-*d*_6_ (*δ* 39.52 ppm), CD_3_OD (*δ* 49.00 ppm), (CD_3_)_2_CO (*δ* 29.84 ppm) for ^13^C NMR spectra were used as references. NMR spectra in the superacids TfOH at room temperature were recorded on Bruker 400 spectrometer at 400 and 100 MHz for ^1^H and ^13^C NMR spectra, respectively. NMR spectra in TfOH were referenced to the signal of CH_2_Cl_2_ added as the internal standard: *δ* 5.30 ppm for ^1^H NMR spectra and *δ* 53.52 ppm for ^13^C NMR spectra. HRMS was carried out with instruments Bruker maXis HRMS-ESI-QTOF and Varian 902-MS MALDI Mass Spectrometer. IR spectra of the compounds in KBr were taken with a FSM-1201 spectrometer. GC-MS spectra were taken with the Shimadzu GCMS QP-2010 SE machine. The preparative reactions were monitored by thin-layer chromatography carried out on silica gel plates (Alugram SIL G/UV-254), using UV light for detection.

The study of oligomeric products was carried out using a TripleTOF 5600+ high-resolution quadrupole time-of-flight (QTOF) mass spectrometer (AB Sciex, Concord, ON, Canada) equipped with a Duospray ion source with ESI probe. A mass spectrometer was combined with an LC-30 Nexera HPLC system (Shimadzu, Kyoto, Japan) consisting of a DGU-5A vacuum degasser, two LC-30AD chromatographic pumps, an SIL-30AC autosampler, and an STO-20A column thermostat.

Chromatographic separation was achieved at 40 °C on a Nucleodur PFP column (Macherey-Nagel, Duren, Germany) with a pentafluorophenyl-propyl stationary phase, 150×2 mm, particle size 1.8 μm. A mixture of water (A) and acetonitrile (B) containing 0.1% formic acid was used as a mobile phase. The gradient elution was programmed as follows: 0–3 min: 10% B, 3–40 min: ramp to 100% B, and 40–45 min: 100% B. The mobile phase flow rate was 0.3 mL/min, and the injection volume was 5 µL. Nontargeted screening of reaction products was performed in a data-dependent acquisition mode using positive electrospray ionization (ESI+). The following ion source parameters were used: nebulizing, drying, and gas curtain pressure—40, 40, and 30 psi, respectively, capillary voltage—5500 V, and source temperature—400 °C. The parameters used for recording the mass spectra in a TOF MS mode were as follows: declusterization potential—80 V, *m*/*z* range—150–1200, and acquisition time—150 ms. Tandem (CID) mass spectra were recorded for precursor ions with signal intensities above a threshold of 100 cps. Nitrogen was used as the collision gas and collision energy—50 eV with a spread of 30 eV. The maximum number of simultaneously fragmented precursor ions—15, *m*/*z* range—20–1200. Data processing was performed using MasterView and Formula Finder (AB Sciex, Concord, ON, Canada) software packages. Elemental compositions of the detected compounds were determined based on the accurate masses of ions, their isotopic distributions, and product ions *m*/*z*. The following constraints were applied: maximal number of atoms: C—100, H—300, O—20, mass error < 5 ppm (MS) and <10 ppm (MS/MS), and signal-to-noise ratio (S/N) > 10.

### 4.2. DFT Calculations

All computations were carried out at the DFT/HF hybrid level of theory using hybrid exchange functional B3LYP by using GAUSSIAN 2009 program packages [32]. The geometries optimization was performed using the 6-311+G(2d,2p) basis set (standard 6-311G basis set added with polarization (d,p) and diffuse functions). Optimizations were performed on all degrees of freedom, and solvent phase optimized structures were verified as true minima with no imaginary frequencies. The Hessian matrix was calculated analytically for the optimized structures in order to prove the location of correct minima and to estimate the thermodynamic parameters. Solvent-phase calculations used the Polarizable Continuum Model (PCM, solvent = water).

### 4.3. Study of Biological Activity

MICs of furan compounds against *Escherichia coli* ATCC 25922, *Staphylococcus aureus* ATCC 29213, and *Candida albicans* ATCC 10231 were determined using broth microdilution as described in ISO 20776-1:2019 and ISO 16256:2021. Stock solutions of furan compounds in neat (pure) DMSO were prepared in sterile tubes and used on the same day. Two-fold dilutions of the furan compounds in the appropriate culture medium were added to the wells of a 96-well plate. The final concentrations of the test substances (after inoculation) were 256, 128, 64, 32, 16, 8, 4, 2, and 1 µg/mL. Solutions of furan compounds were added to the wells of the plates, 50 µL per well for *S. aureus* and *E. coli* and 100 µL for *C. albicans*.

RPMI-1640 medium, buffered with MOPS (3-(N-morpholino)propanesulfonic acid) containing l-glutamine and lacking sodium bicarbonate was used for *C. albicans*. The medium for *E. coli* and *S. aureus* was Mueller–Hinton broth. The fungus inoculum was prepared in the test medium and adjusted to match the turbidity of a 0.5 McFarland standard. A 1:100 dilution followed by a 1:20 dilution was performed for the yeast strain to obtain a final inoculum ranging from 0.5 to 2.5 × 10^3^ CFU/mL. Then, 100 µL of the fungal inoculum was added to each well containing furan compounds.

Bacterial inoculums were prepared in sterile sodium chloride solution and adjusted to the 0.5 McFarland standard. A volume of 50 µL of this suspension was diluted in 10 mL of Mueller–Hinton broth until a concentration of approximately 5 × 10^5^ CFU/mL was reached. Of this suspension, 50 μLwas inoculated into each furan compounds-containing wells.

To ensure that the inoculum contained the required number of cells, the viability of the inoculum suspensions was counted. One hundred microliters of the inoculum was taken from the growth control tube immediately after inoculation and diluted in 9.9 mL of sodium chloride solution. One hundred microliters of this dilution were applied to the surface of a suitable agar plate (Sabouraud dextrose agar plate for *C. albicans* and Trypticase soy agar plate for *S. aureus* and *E. coli*), which were then incubated overnight.

After inoculation, the plates were incubated at 37 °C for 18 h for bacterial strains, 22 h for *C. albicans*. The susceptibility to furans was assessed on the basis of visual observation of growth the strains in the culture media. The minimal inhibitory concentration (MIC) is the lowest concentration of an antimicrobial that inhibits visible growth of a bacterial culture under a defined set of experimental conditions.

### 4.4. Preparation and Characterization of Compounds

#### 4.4.1. General Procedure for Synthesis of 3-(furan-2-yl)propenoic Acids 1a–f from furan-2-carbaldehydes and Malonic Acid

Malonic acid (0.91 g, 8.9 mmol) and substituted furan-2-carbaldehyde (8.9 mmol) were added to pyridine (10 mL). Then, piperidine (0.23 g, 2.7 mmol) was added dropwise for 5 min, and the mixture was stirred 4 h at 115 °C. The mixture was poured into water (50 mL), and aqueous HCl was added to a slightly acidic medium (pH 5–6), while orange precipitate was observed. A precipitate was filtered off and washed with water.

#### 4.4.2. General Procedure for Synthesis of 3-(furan-2-yl)-3-phenylpropanoic Esters 1g-i from 3-(furan-2-yl)propeonic Acids 1a,e,f

The solution of NaOH (0.29 g, 7.2 mmol) in MeOH (3 mL) was added to a stirring mixture of acids **1** (7.2 mmol) in MeOH (5 mL). Dimethyl sulfate (1.21 g, 8.6 mmol) was added dropwise for 5 min, and the mixture was stirred for 1h at 60 °C. The mixture was poured into water (50 mL) and extracted with diethyl ether (3 × 50 mL). The extracts were combined, washed with water, and dried with Na_2_SO_4_; the solvent was distilled under reduced pressure.

#### 4.4.3. General Procedure for Synthesis of 3-(furan-2-yl)-3-phenylpropanoic Acids and Esters 2a-r from Compounds **1** and Arenes in TfOH

To the mixture of compound **1** (0.36 mmol), arene (0.1 mL), and CH_2_Cl_2_ (1 mL) was added TfOH (0.5 mL, 6.45 mmol). The reaction mixture was stirred at 0 °C for 2 h and poured into water (50 mL) and extracted with chloroform (3 × 50 mL). The combined extract was washed with water (3 × 50 mL) and dried over Na_2_SO_4_; the solvent was distilled under a reduced pressure.

#### 4.4.4. General Procedure for Synthesis of 3-(furan-2-yl)-3-phenylpropanoic Acids and Esters 2 from Compounds **1** and Benzene under the Action of AlX_3_ (X = Cl, Br)

Compound **1** (0.36 mmol) was added to a suspension of AlX_3_ (1.8 mmol) in benzene (2 mL) at room temperature. The reaction mixture was stirred at room temperature for 1 h and poured into 50 mL of water. Extracted with ethyl acetate (3 × 30 mL), the combined organic extracts with washed with water (3 × 50 mL) and dried with Na_2_SO_4_. The solvent was distilled under a reduced pressure.

**3-(Furan-2-yl)propenoic acid (1a)** [32] was obtained as light orange solid from furan-2-carbaldehyde in a yield of 53%. M.p. 143–145 °C (lit. 140 °C [33]). ^1^H NMR (500 MHz, CDCl_3_): *δ* = 6.32 d (1H, =CH, *J =* 15.7 Hz), 6.49–6.50 m (1H_hetarom._), 6.67 d (1H_hetarom._, *J =* 3.4 Hz), 7.51–7.54 m (2H).^13^C NMR (125 MHz, CDCl_3_): *δ* = 112.6, 115.0, 115.9, 133.2, 145.4, 150.8, 172.6. IR (KBr), cm^–1^: ~3000 (O–H), 1699 (C=O). GC-MS, *m*/*z*, (I_rel_., %): 138 (100) [M]^+^, 121 (38), 110 (30), 92 (27), 81 (20), 65 (46), 53 (11).

**3-(5-(Hydroxymethyl)furan-2-yl)propenoic acid 1b** were obtained from 5-hydroxymethylfuran-2-carbaldehyde as mixture of *E*-/*Z*-isomers in a ratio of 1:0.6 with m.p. 114–117 °C for the mixture.

***E*-3-(5-Hydroxymethylfuran-2-yl)propenoic acid (1b)** [33]. Yield 50%. Light orange solid.^1^H NMR (500 MHz, CD_3_OD), from the spectrum of the mixture of isomers: *δ* = 4.55 s (2H, CH_2_), 6.24 d (1H, =CH, *J =* 15.7 Hz), 6.42 d (1H, H_hetarom._, *J* = 3.3 Hz), 6.69 d (1H, H_hetarom._, *J* = 3.3 Hz), 7.40 d (1H, =CH, *J =* 15.7 Hz). ^13^C NMR (125 MHz, CD_3_OD), from the spectrum of the mixture of isomers: *δ* = 57.5, 111.0, 116.2, 117.2, 132.8, 151.8, 159.0, 170.4. IR (KBr), cm^–1^: ~3000 (O–H), 1696 (C=O). HRMS, for the mixture of isomers, *m*/*z* calculated for C_8_H_8_O_4_ [M+H]: 169.0495. Found: 169.0497.

***Z*-3-(5-Hydroxymethylfuran-2-yl)propenoic acid (1b):** Yield 30%. Light orange solid. ^1^H NMR (500 MHz, CD_3_OD), from the spectrum of the mixture of isomers: *δ* = 4.55 s (2H, CH_2_), 6.17 d (1H, =CH, *J =* 15.7 Hz), 6.31 d (1H_hetarom._, *J* = 3.3 Hz), 6.69 d (1H_hetarom._, *J* = 3.3 Hz), 7.38 d (1H_arom._, *J =* 15.7 Hz). ^13^C NMR (125 MHz, CD_3_OD), from the spectrum of the mixture of isomers: *δ* = 28.3, 111.0, 115.8, 117.6, 132.7, 151.5, 155.4, 170.4. HRMS, for the mixture of isomers, *m*/*z* calculated for C_8_H_8_O_4_ [M+H]: 169.0495. Found: 169.0497.

**3-(5-Methoxymethylfuran-2-yl)propenoic acid (1c)** was obtained as a dark orange solid from 5(methoxymethylfuran-2-carbaldehyde in a yield of 49%.M.p. 103–104 °C. ^1^H NMR (500 MHz, CDCl_3_): *δ* = 3.41 s (3H, Me), 4.43 s (2H, CH_2_), 6.33 d (1H, =CH, *J =* 15.7 Hz), 6.42 d (1H_hetarom._, *J =* 3.3 Hz), 6.62 d (1H_hetarom._, *J =* 3.3 Hz), 7.48 d (1H, =CH, *J =* 15.7 Hz). ^13^C NMR (125 MHz, CDCl_3_): *δ* = 58.4, 66.6, 111.9, 115.2, 116.6, 132.9, 150.9, 155.0, 172.2. IR (KBr), cm^–1^: ~3000 (O–H), 1689 (C=O). HRMS, *m*/*z* calculated for C_9_H_10_O_4_ [M+H]: 183.0652. Found: 183.0653.

**3-(5-Benzylfuran-2-yl)propenoic acid (1d)** was obtained as a dark orange solid from 5-benzylfuranfuran-2-carbaldehyde in a yield of 83%.M.p. 118–120 °C. ^1^H NMR (500 MHz, CDCl_3_): *δ* = 4.00 s (2H, CH_2_), 6.08 d (1H_hetarom._, *J =* 3.2 Hz), 6.23 d (1H, =CH, *J =* 15.6 Hz), 6.60 d (1H_hetarom._, *J =* 3.3 Hz), 7.25–7.27 m (3H), 7.33 t (2H_arom._, *J =* 7.1 Hz), 7.44 d (1H, =CH, *J =* 15.6 Hz).^13^C NMR (125 MHz, CDCl_3_): *δ* = 35.0, 109.7, 113.7, 117.4, 127.0, 128.8, 129.0, 133.2, 137.1, 150.0, 158.6, 172.6. IR (KBr), cm^–1^: ~3000 (O–H), 1691 (C=O). HRMS, *m*/*z* calculated for C_14_H_12_O_3_ [M+H]: 229.0859. Found: 229.0861.

**3-[5-(2-Carboxyethenyl)furan-2-yl]propenoic acid(1e)** [34] was obtained as a light orange solid from furan-2,5-dicarbaldehyde in a yield of 48%. M.p. 284–286 °C. ^1^H NMR (500 MHz, CDCl_3_): *δ* = 3.68 s (2H, OH), 6.37 d (2H, =CH, *J* = 15.5 Hz), 6.99 s (2H_hetarom._), 7.37 d (2H, =CH, *J* = 15.5 Hz). ^13^C NMR (125 MHz, CDCl_3_): *δ* = 117.3, 118.1, 129.7, 151.8, 166.9. IR (KBr), cm^–1^: ~3000 (O–H), 1680 (C=O). HRMS, *m*/*z* calculated for C_10_H_9_O_5_ [M+H]: 209.0445. Found: 209.0447.

**3-(Benzofuran-2-yl)propenoic acid (1f)** [35] was obtained as a light orange solid from benzofuran-2-carbaldehyde in a yield of 65%. M.p. 225–227 °C. ^1^H NMR (500 MHz, (CD_3_)_2_CO): *δ* = 6.49–6.53 m (1H), 7.27–7.20 m (2H), 7.41–7.44 m (1H), 7.56–7.58 m (1H), 7.60–7.64 m (1H), 7.69–7.70 m (1H). ^13^C NMR (125 MHz, (CD_3_)_2_CO): *δ* = 112.1, 112.2, 119.9, 122.9, 124.4, 127.5, 129.5, 132.3, 153.4, 154.0, 156.5, 167.3. IR (KBr), cm^–1^: ~3000(O–H), 1700 (C=O).

**Methyl 3-(furan-2-yl)propenoate (1g)** [36] was obtained as a dark orange oil from acid **1a** in a yield of 60%.^1^H NMR (500 MHz, CDCl_3_): *δ* = 3.78 s (3H, Me), 6.31 d (1H, =CH, J = 15.8 Hz), 6.45–6.46 m (1H_hetarom._), 6.61 d (1H, H_hetarom._, J = 3.4 Hz), 7.41–7.48 m (2H). ^13^C NMR (125 MHz, CDCl_3_): *δ* = 51.7, 112.4, 114.9, 115.6, 131.3, 144.9, 151.0, 167.6. IR (KBr), cm^–1^: ~3000(O–H), 1696 (C=O).GC-MS, *m*/*z*, (I_rel_., %): 152 (58) [M]^+^, 121 (100), 65 (38), 53(4).

**Methyl 3-[5-(2-methylcarbonylethenyl)furan-2-yl]propenoate (1h)** [34] was obtained as a yellow solid from acid **1e** in a yield of 92%.M.p. 115–118 °C.^1^H NMR (500 MHz, CDCl_3_): *δ* = 3.80 s (3H, Me), 6.42 d (1H, =CH, *J* = 15.8 Hz), 6.65 s (1H_hetarom._), 7.39 d (1H, =CH, *J* = 15.8 Hz). ^13^C NMR (125 MHz, CDCl_3_): *δ* = 51.0, 115.9, 116.7, 129.4, 151.6, 166.2. IR (KBr), cm^–1^: 1694 (C=O).

**Methyl 3-(benzofuran-2-yl)propenoate (1i)** [37] was obtained as a light orange solid from acid **1e** in a yield of 53%.M.p. 224–226 °C. ^1^H NMR (500 MHz, CDCl_3_): *δ* = 3.81 s (3H, Me), 6.58 d (1H, =CH, *J* = 15.7 Hz), 6.91 s (1H_hetarom._), 7.23 t (1H_arom._, *J* = 7.6 Hz), 7.35 t (1H_arom._, *J* = 8.0 Hz), 4.74 d (1H_arom._, *J* = 16.5 Hz), 7.58–7.53 m (2H). ^13^C NMR (125 MHz, CDCl_3_): *δ* = 51.9, 111.3, 111.5, 118.5, 121.8, 123.4, 126.5, 128.4, 131.5, 152.4, 155.6, 167.2. IR (KBr), cm^–1^: 1698 (C=O).

**3-(Furan-2-yl)-3-phenylpropanoic acid (2a)** was obtained as a light orange oil from acid **1a** and benzene in TfOH in yields of 33% (in TfOH), 52% (under the action of AlBr_3_), and 65% (under the action of AlCl_3_) (see Table 1). NMR ^1^H (500 MHz, CDCl_3_): *δ* = 2.94 dd (1H, CH_2_, *J =* 16.2, 7.7 Hz), 3.15 dd (1H, CH_2_, *J =* 16.2, 7.7 Hz), 4.54 t (1H, CH, *J =* 7.7 Hz), 6.06 (1H_hetarom._, *J =* 3.0 Hz), 6.29 dd (1H_hetarom._, *J =* 3.0, 1.9 Hz), 7.25–7.32 m (6H_arom._). NMR ^13^C (125 MHz, CDCl_3_): *δ* = 39.5, 41.1, 106.0, 110.3, 127.3, 127.8, 128.8, 141.0, 141.9, 156.0, 177.4. IR (KBr), cm^–1^: ~3000(O–H), 1701 (C=O).GC-MS, *m*/*z*, (I_rel._, %): 216 (16) [M]^+^, 157 (100), 141 (11), 128 (30), 115 (12), 77 (8), 65 (4). HRMS, *m*/*z* calculated for C_13_H_12_O_3_[M+H]: 217.0859. Found: 217.0859.

**3-(Furan-2-yl)-3-(4-methylphenyl)propanoic acid (2b)** was obtained as a light orange oil from acid **1a** and toluene in TfOH in a yield of 92%. ^1^H NMR (500 MHz, CDCl_3_): *δ* = 2.32 s (3H, Me), 2.92 dd (1H, CH_2_, *J =* 16.1, 7.7 Hz), 3.13 dd (1H, CH_2_, *J =* 16.1, 7.7 Hz), 4.50 t (1H, CH, *J =* 7.7 Hz), 6.04 d (1H_hetarom._, *J =* 3.1 Hz), 6.28 m (1H_hetarom._), 7.11–7.20 m (4H_arom._), 7.31 br.s (1H_hetarom._). ^13^C NMR (125 MHz, CDCl_3_): *δ* = 21.1, 39.5, 40.8, 105.8, 110.2, 127.7, 129.5, 136.9, 138.0, 141.9, 156.3, 177.2. IR (KBr), cm^–1^: ~3000 (O–H), 1698 (C=O).HRMS, *m*/*z* calculated for C_14_H_14_O_3_ [M+H]: 231.1016. Found: 231.1018.

**3-(Furan-2-yl)-3-(3,4-dimethylphenyl)propanoic acid (2c)** was obtained as a light orange oil from acid **1a** and *ortho*-xylene in TfOH in a yield of 86%. ^1^H NMR (500 MHz, CDCl_3_): *δ* = 2.22 s (6H, 2Me), 2.91 dd (1H, CH_2_, *J* = 16.1, 7.6 Hz), 3.11 dd (1H, CH_2_, *J* = 16.1, 8.0 Hz), 4.46 t (1H, CH, *J* = 7.7 Hz), 6.04 d (1H_hetarom._, *J* = 3.1 Hz), 6.26–6.27 m (1H_hetarom._), 6.97–7.00 m (2H_arom._), 7.05–7.07 m (1H_arom._), 7.30 br.s (1H_hetarom._). ^13^C NMR (125 MHz, CDCl_3_): *δ* = 19.5, 20.0, 39.5, 40.8, 105.8, 110.2, 125.1, 129.1, 130.0, 135.5, 136.9, 138.5, 141.8, 156.4, 177.1. IR (KBr), cm^–1^: ~3000 (O–H), 1701 (C=O).HRMS, *m*/*z* calculated for C_15_H_16_O_3_ [M+H]: 245.1172. Found: 245.1173.

**3-(Furan-2-yl)-3-(2,4-dimethylphenyl)propanoic acid (2d)** was obtained as a light orange oil from acid **1a** and *meta*-xylene in TfOH in a yield of 84%. ^1^H NMR (500 MHz, CDCl_3_): *δ* = 2.28 s (3H, Me), 2.36 s (3H, Me), 2.91 dd (1H, CH_2_, *J =* 16.3, 7.3 Hz), 3.11 dd (1H, CH_2_, *J =* 16.3, 8.1 Hz), 4.75 t (1H, CH, *J =* 7.7 Hz), 5.98 d (1H_hetarom._, *J =* 3.2 Hz), 6.25–6.26 m (1H_hetarom._), 6.96–6.99 m (2H_arom._), 7.03–7.04 m (1H_arom._), 7.29–7.30 m (1H_hetarom._).^13^C NMR (125 MHz, CDCl_3_): *δ* = 19.5, 21.1, 36.6, 38.8, 105.9, 110.2, 126.8, 127.2, 131.6, 135.9, 136.1, 136.6, 141.8, 156.3, 177.0. IR (KBr), cm^–1^: ~3000 (O–H), 1699 (C=O).HRMS, *m*/*z* calculated for C_15_H_16_O_3_ [M+H]: 245.1172. Found: 245.1172.

**3-(Furan-2-yl)-3-(2,5-dimethylphenyl)propanoic acid (2e)** was obtained as a light orange oil from acid **1a** and *para*-xylene in TfOH in a yield of 55%. ^1^H NMR (500 MHz, CDCl_3_): *δ* = 2.27 s (3H, Me), 2.36 s (3H, Me), 2.91 dd (1H, CH_2_, *J =* 16.3, 7.0 Hz), 3.12 dd (1H, CH_2_, *J =* 16.3, 8.3 Hz), 4.75 t (1H, CH, *J =* 7.6 Hz), 6.00 d (1H_hetarom._, *J =* 2.8 Hz), 6.26–6.27 m (1H_hetarom._), 6.94–6.96 m (2H_arom._), 7.04–7.07 m (1H_arom._), 7.31 br.s (1H_hetarom._). ^13^C NMR (125 MHz, CDCl_3_): *δ* = 19.1, 21.3, 36.8, 38.7, 106.0, 110.2, 127.6, 127.9, 130.7, 132.9, 135.8, 138.99, 141.8, 156.1, 177.0. IR (KBr), cm^–1^: ~3000 (O–H),1702 cm^–1^.HRMS, *m*/*z* calculated for C_15_H_16_O_3_ [M+H]: 245.1172. Found: 245.1172.

**3-(Furan-2-yl)-3-(2,4,6-trimethylphenyl)propanoic acid (2f)** was obtained as a light orange oil from acid **1a** and mesitylene in TfOH in a yield of 98%. ^1^H NMR (500 MHz, CDCl_3_): *δ* = 2.26 s (3H, Me), 2.29 s (6H, Me), 2.89 dd (1H, CH_2_, *J =* 18.6, 6.0 Hz), 3.34 dd (1H, CH_2_, *J =* 18.3, 8.6 Hz), 5.07 t (1H, CH, *J =* 6.7 Hz), 5.97 br.s (1H_hetarom._), 6.30 m (1H_hetarom._), 6.82–6.84 m (2H_arom._), 7.30 br.s (1H_hetarom._). ^13^C NMR (125 MHz, CDCl_3_): *δ* = 20.9, 21.3, 35.7, 36.7, 105.4, 110.4, 127.1, 134.5, 136.6, 137.0, 137.9, 141.2, 156.0, 178.1. IR (KBr), cm^–1^: ~3000 (O–H), 1705 (C=O).HRMS, *m*/*z* calculated for C_16_H_19_O_3_[M+H]: 259.1329. Found: 259.1330.

**3-(5-Benzylfuran-2-yl)-3-phenylpropanoic acid (2g)** was obtained as a dark orange oil from benzene under the action of TfOH in yields of 43% (from acid **1b**), 46% (from acid **1c**), 63% (from acid **1d**), and 75% (fromacid **1d** under the action of AlCl_3_) (see Table 2 NMR ^1^H (500 MHz, CDCl_3_): *δ* = 2.91 dd (1H, CH_2_, *J =* 16.1, 7.8 Hz), 3.11 dd (1H, CH_2_, *J =* 16.1, 7.8 Hz), 3.90 s (2H, CH_2_-Ar), 4.49 t (1H, CH, *J =* 7.8 Hz), 5.84 d (1H_hetarom._, *J =* 2.6 Hz), 5.91 d (1H_hetarom._, *J =* 2.6 Hz), 7.18–7.24 m (6H_arom._), 7.25–7.31 m (4H_arom._). NMR ^13^C (125 MHz, CDCl_3_): *δ* = 34.6, 41.3, 106.7, 106.9, 126.5, 127.2, 127.9, 128.4, 128.4, 128.5, 128.7, 128.8, 141.2, 145.2, 154.0, 181.3.).IR (KBr), cm^–1^: ~3000 (O–H), 1700 (C=O).HRMS, *m*/*z* calculated for C_20_H_18_O_3_[M+H]: 307.1329. Found: 307.1327.

**Methyl 3-(furan-2-yl)-3-phenylpropanoate (2h)** was obtained as a dark orange oil from ester **1g** and benzene in TfOH in a yield of 98%. NMR ^1^H (500 MHz, CDCl_3_): *δ* = 2.90 dd (1H, CH_2_, *J =* 15.7, 7.8 Hz), 3.10 dd (1H, CH_2_, *J =* 15.7, 7.8 Hz), 3.61 s (3H, Me), 4.55 t (1H, CH, *J =* 7.8 Hz), 6.05 d (1H_hetarom._, *J =* 3.0 Hz), 6.28 dd (1H_hetarom._, *J =* 1.9, 3.0 Hz), 7.23–7.26 m (3H_arom._), 7.29–7.32 m (3H_arom._). NMR ^13^C (125 MHz, CDCl_3_): *δ* = 39.7, 41.5, 52.0, 105.9, 110.3, 127.2, 127.9, 128.8, 139.2, 141.3, 141.9, 172.0. GC-MS, *m*/*z*, (I_rel_., %): 230 (13) [M]^+^, 170 (25), 157 (100), 128 (27). IR (KBr), cm^–1^: 1699 (C=O). HRMS, *m*/*z* calculated for C_14_H_14_O_3_ [M+H]: 231.1016. Found: 231.1015.

**Methyl 3-(furan-2-yl)-3-(4-methylphenyl)propanoate (2i)** was obtained as a light orange oil from ester **1g** and toluene in TfOH in a yield of 84%.^1^H NMR (500 MHz, CDCl_3_): *δ* = 2.31 s (3H, Me), 2.89 dd (1H, CH_2_, *J =* 15.3, 7.9 Hz), 3.09 dd (1H, CH_2_, *J =* 14.5, 7.9 Hz), 3.62 s (3H, Me), 4.52 t (1H, CH, *J =* 7.9 Hz), 6.04 d (1H_hetarom._, *J =* 3.1 Hz), 6.26–6.27 m (1H_hetarom._), 7.10–7.15 m (4H_arom._), 7.31 br.s (1H_hetarom._). ^13^C NMR (125 MHz, CDCl_3_): *δ* = 21.1, 39.7, 41.1, 51.8, 105.7, 110.2, 127.7, 129.4, 136.7, 138.3, 141.8, 156.6, 172.0. IR (KBr), cm^–1^: 1698 (C=O). HRMS, *m*/*z* calculated for C_15_H_16_O_3_ [M+H]: 245.1172. Found: 245.1174.

**Methyl 3-(furan-2-yl)-3-(3,4-dimethylphenyl)propanoate (2j)** was obtained as a light orange oil from ester**1g** and *ortho*-xylene in TfOH in a yield of 89%.^1^H NMR (500 MHz, CDCl_3_): *δ* = 2.22 s (3H, Me), 2.23 s (3H, Me), 2.88 dd (1H, CH_2_, *J =* 16.1, 7.8 Hz), 3.08 dd (1H, CH_2_, *J =* 16.1, 8.0 Hz), 3.62 s (3H, Me), 4.49 t (1H, CH, *J =* 7.8 Hz), 6.05 d (1H_hetarom._, *J =* 3.0 Hz), 6.27–6.28 m (1H_hetarom._), 6.97–7.02 m (2H_arom._), 7.06–7.07 m (1H_arom._), 7.31 br.s (1H_hetarom._). ^13^C NMR (125 MHz, CDCl_3_): *δ* = 19.5, 20.0, 39.7, 41.1, 51.8, 105.6, 110.2, 125.1, 129.1, 130.0, 135.4, 136.9, 138.7, 141.8, 156.7, 172.1. IR (KBr), cm^–1^: 1700 (C=O). HRMS, *m*/*z* calculated for C_16_H_18_O_3_ [M+H]: 259.1329. Found: 259.1331.

**Methyl 3-(furan-2-yl)-3-(2,4-dimethylphenyl)propanoate (2k)** was obtained as a light orange oil from ester**1g** and *meta*-xylene in TfOH in a yield of 88%.^1^H NMR (500 MHz, CDCl_3_): *δ* = 2.28 s (3H, Me), 2.37 s (3H, Me), 2.88 dd (1H, CH_2_, *J =* 15.8, 7.3 Hz), 3.08 dd (1H, CH_2_, *J =* 15.8, 8.2 Hz), 3.62 s (3H, Me), 4.76 t (1H, CH, *J =* 7.7 Hz), 5.97 d (1H_hetarom._, *J =* 2.7 Hz), 6.24–6.25 m (1H_hetarom._), 6.95–6.98 m (2H_arom._), 7.02–7.04 m (1H_arom._), 7.29 br.s (1H_hetarom._). ^13^C NMR (125 MHz, CDCl_3_): *δ* = 19.5, 21.1, 36.9, 39.0, 51.9, 105.8, 110.2, 126.9, 127.1, 131.6, 135.9, 136.3, 136.5, 141.7, 156.5, 172.2. IR (KBr), cm^–1^: 1701 (C=O). HRMS, *m*/*z* calculated for C_16_H_18_O_3_[M+H]: 259.1329. Found: 259.1330.

**Methyl 3-(furan-2-yl)-3-(2,5-dimethylphenyl)propanoate (2l)** was obtained as a light orange oil from ester**1g** and *para*-xylene in TfOH in a yield of 74%. ^1^H NMR (500 MHz, CDCl_3_): *δ* = 2.27 s (3H, Me), 2.37 s (3H, Me), 2.90 dd (1H, CH_2_, *J =* 15.8, 7.1 Hz), 3.09 dd (1H, CH_2_, *J =* 17.0, 8.4 Hz), 3.63 s (3H, OMe), 4.78 t (1H, CH, *J =* 7.8 Hz), 6.00 d (1H_hetarom._, *J =* 3.1 Hz), 6.26 dd (1H, H_hetarom._, *J =* 1.9, 3.1 Hz), 6.94–6.95 (2H_arom._), 7.04–7.06 m (1H_arom._), 7.30 d (1H_hetarom._, *J =* 1.9 Hz). ^13^C NMR (125 MHz, CDCl_3_): *δ* = 19.1, 21.3, 37.1, 39.0, 51.9, 105.9, 110.2, 127.7, 127.8, 130.7, 132.9, 135.8, 139.1, 141.7, 156.4, 172.2. IR (KBr), cm^–1^: 1700 (C=O). HRMS, *m*/*z* calculated for C_16_H_18_O_3_ [M+H]: 259.1329. Found: 259.1330.

**Methyl 3-(furan-2-yl)-3-(2,4,6-trimethylphenyl)propanoate (2m)** was obtained as a light orange oil from ester **1g** and mesitylene in TfOH in a yield of 84%. ^1^H NMR (500 MHz, CDCl_3_): *δ* = 2.25 s (6H, Me), 2.29 s (3H, Me), 2.86 dd (1H, CH_2_, *J =* 15.3, 6.5 Hz), 3.29 dd (1H, CH_2_, *J =* 13.5, 8.5 Hz), 3.66 s (3H, OMe), 5.08 t (1H, CH, *J =* 6.9 Hz), 5.95 br.s (1H_hetarom._), 6.28–6.29 m (1H_hetarom._), 6.81–6.83 m (2H_arom._), 7.29 br.s (1H_hetarom._). ^13^C NMR (125 MHz, CDCl_3_): *δ* = 20.9, 36.0, 36.7, 51.9, 105.3, 110.3, 127.0, 128.5, 134.6, 136.5, 137.0, 141.2, 156.3, 172.7. IR (KBr), cm^–1^: 1699 (C=O). HRMS, *m*/*z* calculated for C_16_H_20_O_3_[M+H]: 273.1485. Found: 273.1487.

Methyl 3-(furan-2-yl)-3-(2,3,5,6-tetramethylphenyl)propanoate (2n) and methyl 3-(furan-2-yl)-3-(2,3,4,5-tetramethylphenyl)propanoate (2o) were obtained as an oily mixture of regioisomers in a ratio of 1:0.2 from ester 1g and durene in TfOH in a general yield of 84%.

**Methyl 3-(furan-2-yl)-3-(2,3,5,6-tetramethylphenyl)propanoate(2n)** was obtained in a yield of 70%. ^1^H NMR (500 MHz, CDCl_3_), from the spectrum of a mixture of isomers: *δ* = 2.18–2.25 m (12H, Me), 2.83 dd (1H, CH_2_, *J =* 15.1, 5.7 Hz), 3.34 dd (1H, CH_2_, *J =* 14.6, 8.2 Hz), 5.24 t (1H, CH, *J =* 6.8 Hz), 5.91–5.94 m (1H_hetarom._), 6.28–6.29 m (1H_hetarom._), 6.85–6.94 m (1H_arom._), 7.29 br.s (1H_hetarom._). ^13^C NMR (125 MHz, CDCl_3_), from the spectrum of a mixture of isomers: *δ* = 16.0, 16.4, 19.2, 19.3, 19.5, 20.6, 31.0, 36.5, 36.9, 37.4, 39.1, 51.9, 56.0, 105.1, 105.7, 110.1, 110.4, 111.6, 121.0, 128.2, 129.1, 131.2, 132.1, 133.2, 133.7, 137.8, 141.0, 141.7, 156.9, 172.9.IR (KBr), for a mixture of isomers, cm^–1^: 1701 (C=O). HRMS, for a mixture of isomers, *m*/*z* calculated for C_15_H_22_O_3_[M+H]: 287.1642. Found: 287.1641.

**Methyl 3-(furan-2-yl)-3-(2,3,4,5-tetramethylphenyl)propanoate (2o)** was obtained in a yield of 14%. ^1^H NMR (500 MHz, CDCl_3_), from the spectrum of a mixture of isomers: *δ* = 2.18–2.25 m (11H, Me), 2.88–2.96 m (1H), 3.09 dd (1H, CH_2_, *J =* 15.3, 8.2 Hz), 6.00 br.s (1H_hetarom._), 6.26 br.s (1H_hetarom._), 6.85–6.94 m (1H_arom._), 7.29 br.s (1H_hetarom._). ^13^C NMR (125 MHz, CDCl_3_), from the spectrum of a mixture of isomers: *δ* = 16.0, 16.4, 19.2, 19.3, 19.5, 20.6, 31.0, 36.5, 36.9, 37.4, 39.1, 51.9, 56.0, 105.1, 105.7, 110.1, 110.4, 111.6, 121.0, 128.2, 129.1, 131.2, 132.1, 133.2, 133.7, 137.8, 141.0, 141.7, 156.9, 172.9.IR (KBr), for a mixture of isomers, cm^–1^: 1701 (C=O).HRMS, for a mixture of isomers, *m*/*z* calculated for C_15_H_22_O_3_[M+H]: 287.1642. Found: 287.1641.

**Methyl 3-(furan-2-yl)-3-(4-methoxyphenyl)propanoate(2p)** and **methyl 3-(furan-2-yl)-3-(2-methoxyphenyl)propanoate(2q)** were obtained as an oily mixture of regioisomers in a ratio of 1:0.3 from ester **1g** and anisole in TfOH in a general yield of 71%.

**Methyl 3-(furan-2-yl)-3-(4-methoxyphenyl)propanoate (2p)** was obtained as a light orange oil from ester **1g** in TfOH in a yield of 55%. ^1^H HMR (500 MHz, CDCl_3_), from the spectrum of a mixture of isomers: *δ* =2.84–3.10 (m, AB system, CH_2_, 2H), 3.61 (s, MeO, 3H), 3.78 (s, MeO, 3H), 4.50 (t, *J*=7.8 Hz, CH, 1H), 6.02 (d, *J* = 3.2 Hz, 1H), 6.26–6.27 (m, 1H), 6.84 (d, *J* = 8.7 Hz, 2H_arom._), 7.17 (d, *J* = 8.7 Hz, 2H_arom._), 7.31(br. s, 1H) ^13^C NMR (125 MHz, CDCl_3_), from the spectrum of a mixture of isomers: *δ* =39.7, 40.6, 51.7, 55.2, 105.5, 110.0, 114.0, 128.7, 133.2, 141.6, 156.5, 158.5, 171.9. IR (KBr), for a mixture of isomers, cm^–1^: 1702 (C=O). HRMS, for a mixture of isomers, *m*/*z* calculated for C_15_H_17_O_4_ [M+H]: 261.1121. Found: 261.1123.

**Methyl 3-(furan-2-yl)-3-(2-methoxyphenyl)propanoate (2q)** was obtained as a light orange oil from ester **1g** in TfOH in a yield of 16%.^1^H HMR (500 MHz, CDCl_3_), from the spectrum of a mixture of isomers: *δ* =: 2.90–3.02 m (2H, CH_2_,), 3.62 s (3H, Me), 3.84 s (3H, Me), 5.00–5.02 m (1H, CH,), 6.07 d (1H, *J* =3.0 Hz,), 6.27–6.28 m (1H), 6.83–7.31 m (5H_arom._) ^13^C NMR (125 MHz, CDCl_3_), from the spectrum of a mixture of isomers: *δ* = 34.6, 38.2, 51.6, 55.5, 105.8, 110.8, 119.0, 120.6, 128.0, 128.2, 128.7, 141.4, 156.0, 158.5, 171.9. IR (KBr), for a mixture of isomers, cm^–1^: 1702 (C=O).HRMS, for a mixture of isomers, *m*/*z* calculated for C_15_H_17_O_4_ [M+H]: 261.1121. Found: 261.1123.

**Methyl 3-[5-(2-methylcarbonyl-1-phenylethyl)furan-2-yl]-3-phenylpropenoate(2r)** was obtained as a light orange oil from ester **1h** and benzene as an equimolar mixture of diastereomers in yields of 29% (in TfOH) and 38% (under the action of AlCl_3_). NMR ^1^H (500 MHz, CDCl_3_): *δ* = 2.82–2.87 m (4H), 3.01–3.06 m (4H), 3.56 s (12H, Me), 4.48 t (2H, CH, *J =* 7.8 Hz), 5.58 s (2H), 5.59s (2H), 7.19–7.37 m (40H_arom._). ^13^C NMR (125 MHz, CDCl_3_): *δ* = 39.8, 41.5, 51.8, 106.6, 127.1, 127.8, 128.6, 141.3, 155.4, 172.0. GC-MS, *m*/*z*, (I_rel_., %): 392 (10) [M]^+^, 319 (25), 259 (53), 229 (100), 187 (9). IR (KBr), cm^–1^: 1703 (C=O). HRMS, *m*/*z* calculated for C_24_H_25_O_5_ [M+H]: 393.1697. Found: 393.1691.

**Cation Aa** generated at the protonation of compound **1e** in TfOH. ^1^H NMR (400 MHz, TfOH): *δ* = 6.94 s (2H, CH), 7.52s (2H, CH), 8.31 s (2H_hetarom._). ^13^C NMR (100 MHz, TfOH): *δ* = 110.1, 128.5, 145.0, 155.8, 182.2.

**Cation Ah** generated at the protonation of compound **1h** in TfOH. ^1^H NMR (400 MHz, TfOH): *δ* = 6.08 s (3H, Me), 7.71 s (2H, CH), 8.21 s (2H, CH), 8.96 s (2H_hetarom._). ^13^C NMR (100 MHz, TfOH): *δ* = 62.7, 110.3, 128.0, 143.6, 155.6, 181.5.

## Figures and Tables

**Table 1:** Reaction of acid **1a** with benzene under the action of Brønsted or Lewis acids leading to compound **2a**.

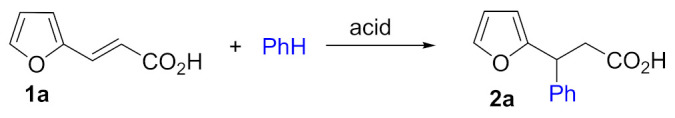				
**Entry**	**Reaction Conditions**			**Yield of 2a, %**
	**Acid (Equiv.)**	**Temperature, °C**	**Time, h**	
1	CF_3_CO_2_H (19)	r.t.	1	no reaction
2	H_2_SO_4_ (52)	r.t.	1	oligomerization
3	FeBr_3_ (5.5)	r.t.	1	oligomerization
4	AlCl_3_ (5.5)	r.t.	1	65
5	AlCl_3_ (5.5)	r.t.	4	47
6	AlBr_3_ (5.5)	r.t.	1	52
7	TfOH (16)	r.t.	1	22
8	TfOH (16)	0	0.25	28
9	TfOH (16)	0	1	32
10	TfOH (16)	0	2	33

**Table 2:** Reaction of acids **1b**–**d** with benzene under the action of Brønsted or Lewis acids leading to compound **2i**.

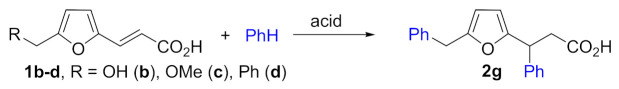					
**Entry**	**Starting Compound 1b**-**d**	**Reaction Conditions**			**Yield of 2g, %**
		**Acid (Equiv.)**	**Temperature, °C**	**Time, h**	
1	**1b**	H_2_SO_4_ (55)	r.t.	1	oligomerization
2	**1b**	AlCl_3_ (5.5)	r.t.	1	44
3	**1b**	AlCl_3_ (5.5)	r.t.	4	17
4	**1b**	TfOH (19)	r.t.	1	37
5	**1b**	TfOH (19)	0	0.25	43
6	**1b**	TfOH (19)	0	1	37
7	**1c**	AlCl_3_ (5.5)	r.t.	1	36
8	**1c**	TfOH (19)	0	0.25	39
9	**1c**	TfOH (19)	0	1	46
10	**1d**	AlCl_3_ (5.5)	r.t.	1	75
11	**1d**	TfOH (19)	0	0.25	60
12	**1d**	TfOH (19)	0	1	63

**Table 3:** The main products detected in reaction mixtures of **1a** and **1b** with benzene in H_2_SO_4_ by HPLC-ESI-HRMS.

Starting Compound	Retention Time, min	Peak Area, Arb. Units	Elemental Composition	RDB ^a^	[M+H]^+^ *m*/*z* (Found)	[M+H]^+^ *m*/*z* (Calcul.)	Δ, ppm
**1a**	10.0	472	C_13_H_14_O_5_	7	251.0914	251.0914	0
	10.9	1400	C_19_H_18_O_7_	11	359.1124	359.1125	−0.4
	11.6	1550	C_20_H_22_O_9_	10	407.1335	407.1337	−0.4
	12.4	900	C_23_H_26_O_10_	11	463.1591	463.1599	−1.7
**1b**	17.7	907	C_28_H_26_O_7_	16	475.1744	475.1751	−1.5
	18.3	410	C_20_H_20_O_4_	11	325.1436	325.1434	0.5
	19.8	712	C_24_H_30_O_6_	10	415.2117	415.2115	0.4
	23.4	1220	C_28_H_28_O_5_	15	445.2008	445.2010	−0.3

Note. ^a^ RDB is a ring and double bond equivalent or unsaturation degree.

**Table 4:** ^13^C NMR data of starting compounds **1e** and **1h** and their protonated forms **Ae** and **Ah** generated in TfOH.

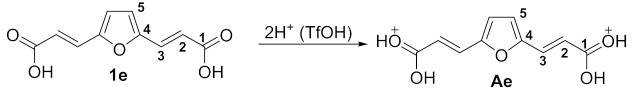						
**Compound/Cation**	**^13^C NMR, *δ*, ppm**					
	C^1^		C^2^	C^3^	C^4^	C^5^
Acid **1e** in CDCl_3_	166.9		129.7	117.3	151.8	118.1
Cation **Ae** in TfOH	182.2		128.5	110.1	155.8	145.0
*Δδ* = *δ***_Ae_** − *δ***_1e_**	15.3		1.2	7.2	4.0	26.9
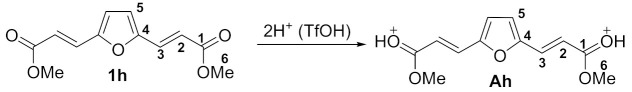						
**Compound/Cation**	**^13^C NMR, *δ*, ppm**					
	C^1^	C^2^	C^3^	C^4^	C^5^	C^6^
Ester **1h** in CDCl_3_	166.2	129.4	116.7	151.8	115.9	51.0
Cation **Ah** in TfOH	181.5	128.0	110.3	155.6	143.6	62.7
*Δδ* = *δ***_Ah_** − *δ***_1h_**	15.3	1.4	6.4	3.8	27.7	11.7

**Table 5:** Selected calculated (DFT) electronic characteristics of the protonated forms of furans, and values of Cribbs energies of protonation reactions (ΔG, kJ/mol).

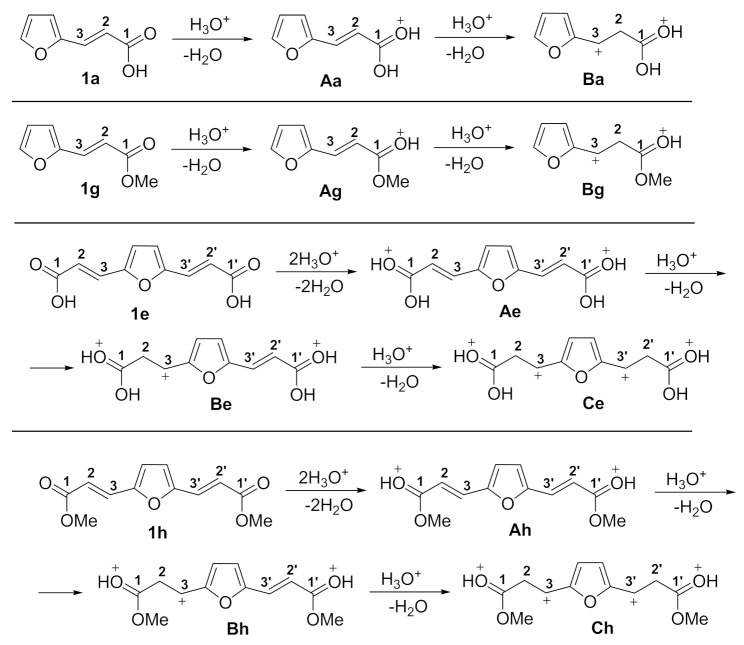									
**Entry**	**Species**	**E_HOMO_, eV**	**E_LUMO_, eV**	**ω, ^a^ eV**	**q(C^1^), ^b^ e**	**q(C^3^), ^b^ e**	**k(C^1^)_LUMO_, ^c^ %**	**k(C^3^)_LUMO_, ^c^ %**	**Δ** **G, ^d^ kJ/mol**
1	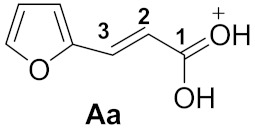	−6.85	−3.28	3.6	0.83	−0.08	22.0	27.1	**1a→Aa** −69.1
2	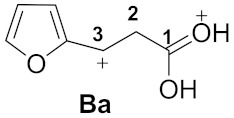	−9.17	−4.63	5.2	0.94	0.02	6.9	30.6	**Aa→Ba** 26.0
3	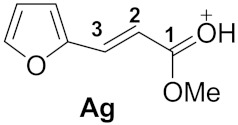	−6.86	−3.34	3.7	0.83	−0.08	23.2	26.0	**1g→Ag** −39.9
4	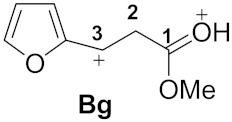	−9.18	−4.63	5.3	0.95	0.02	13.2	27.0	**Ag→Bg** 30.8
5	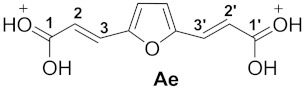	−7.02	−4.06	5.2	0.86	−0.19	11.8	11.6	**1e→Ae** −117.3
6	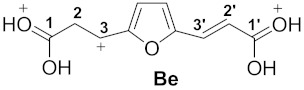	−8.82	−5.25	6.9	**C^1^** 0.89 **C^1^**′ 0.94	**C^3^** −0.17 **C^3^**′ 0.01	**C^1^** 4.3 **C^1^**′ 6.2	**C^3^** 4.0 **C^3^**′ 19.1	**Ae→Be** 53.4
7	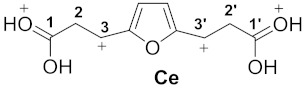	−10.61	−7.28	12.0	0.94	0.24	2.2	21.0	**Be→Ce** 222
8	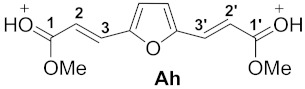	−7.03	−4.13	5.4	0.88	−0.19	12.0	11.4	**1h→Ah** −54.6
9	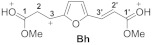	−8.82	−5.27	7.0	**C^1^** 0.91 **C^1^**′ 0.95	**C^3^** −0.18 **C^3^**′ 0.03	**C^1^** 5.2 **C^1^**′ 6.5	**C^3^** 4.6 **C^3^**′ 19.5	**Ah→Bh** 60.7
10	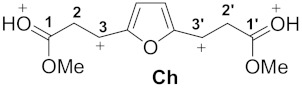	−10.62	−7.28	12.0	0.95	0.24	11.0	10.8	**Bh→Ch** 208

^a^ Global electrophilicity index ω = (E_HOMO_ + E_LUMO_) 2/8(E_LUMO_ − E_HOMO_). ^b^ Natural charges. ^c^ Contribution of atomic orbital into the molecular orbital. ^d^ Gibbs energy of protonation reactions.

## Data Availability

Not applicable.

## Supplementary Materials

The following are available online at https://www.mdpi.com/article/10.3390/molecules27144612/s1, NMR spectra of compounds and cations, study of oligomeric compounds by liquid chromatography-high-resolution mass-spectrometry, study of biological activity of compounds, Data of DFT calculations.

## Sample Availability

Samples of the compounds **1** and **2** are available from the authors.
