# Supplementary material for: Synthesis of 3-Aryl-3-(Furan-2-yl)Propanoic Acid Derivatives, and Study of Their Antimicrobial Activity
Source: Molecules. 2022 Jul 19;27(14):4612. doi: 10.3390/molecules27144612 (PMC9325161; doi:10.3390/molecules27144612)
Supplement: Supplementary file 1 [file molecules-27-04612-s001.zip › molecules-1821228-supplementary.pdf]

# Synthesis of 3-aryl-3-(furan-2-yl)propanoic acid derivatives, and study of their antimicrobial activity

Mikhail V. Kalyaev,[a] Dmitry S. Ryabukhin,[a,b] Marina A. Borisova,[a] Alexander Yu. Ivanov,[c] Irina A. Boyarskaya,[d] Kristina E. Borovkova,[e] Lia R. Nikiforova,[e] Julia V. Salmova,[e] Nikolay V. Ul'yanovskii,[f] Dmitry S. Kosyakov,[f] Aleksander V. Vasilyev*[a,d]

[a]Department of Chemistry, Saint Petersburg State Forest Technical University, Institutsky per., 5, Saint Petersburg, 194021, Russia

[b]All-Russia Research Institute for Food Additives – Branch of V.M. Gorbatov Federal Research Center for Food Systems of RAS, Liteyniy pr., 55, Saint Petersburg, 191014, Russia

[c]Center for Magnetic Resonance, Research Park, Saint Petersburg State University, Universitetskiy pr., 26, Saint Petersburg, Petrodvoretz, 198504, Russia

[d]Department of Organic Chemistry, Institute of Chemistry, Saint Petersburg State University, Universitetskaya nab., 7/9, Saint Petersburg, 199034, Russia

[e]Research and manufacturing company «Home of Pharmacy», Zavodskaya st., 3-245, Kuzmolovskiy t.s., Leningrad oblast, 188663, Russia

[f]Core Facility Center "Arktika", Northern (Arctic) Federal University, nab. Severnoy Dviny, 17, Arkhangelsk, 163002, Russia.

*Corresponding author: A.V. Vasilyev; e-mails: aleksvasil@mail.ru; a.vasilyev@spbu.ru

**Contents**

1. $^{1}$H and $^{13}$C NMR spectra of compounds **1** and **2**……………………………………………S2

2. Results of study of oligomeric compounds by liquid chromatography-high-resolution mass-spectrometry……………………………………………………………………………S27

3. Study of biological activity of compounds **1** and **2**…………………………………………S32

4. $^{1}$H and $^{13}$C NMR spectra of cations **Ae** and **Ah** in TfOH……………………………………S35

5. Data of DFT calculations of compounds **1** and species **A-C**………………………………......S38

# 1. $^{1}$H and $^{13}$C NMR spectra of compounds 1and 2

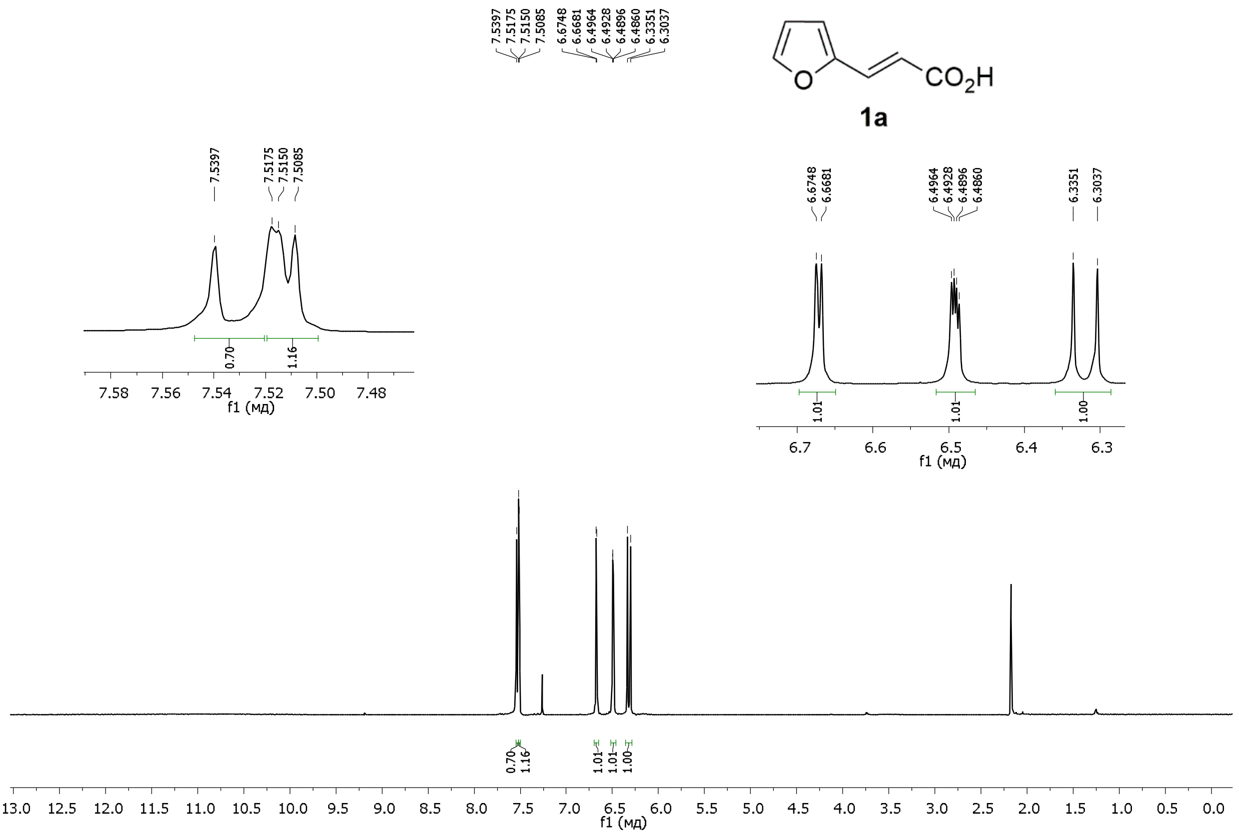


Figure S1. $^{1}$H NMR spectrum of compound **1a** (500 MHz, $CDCl_3$).

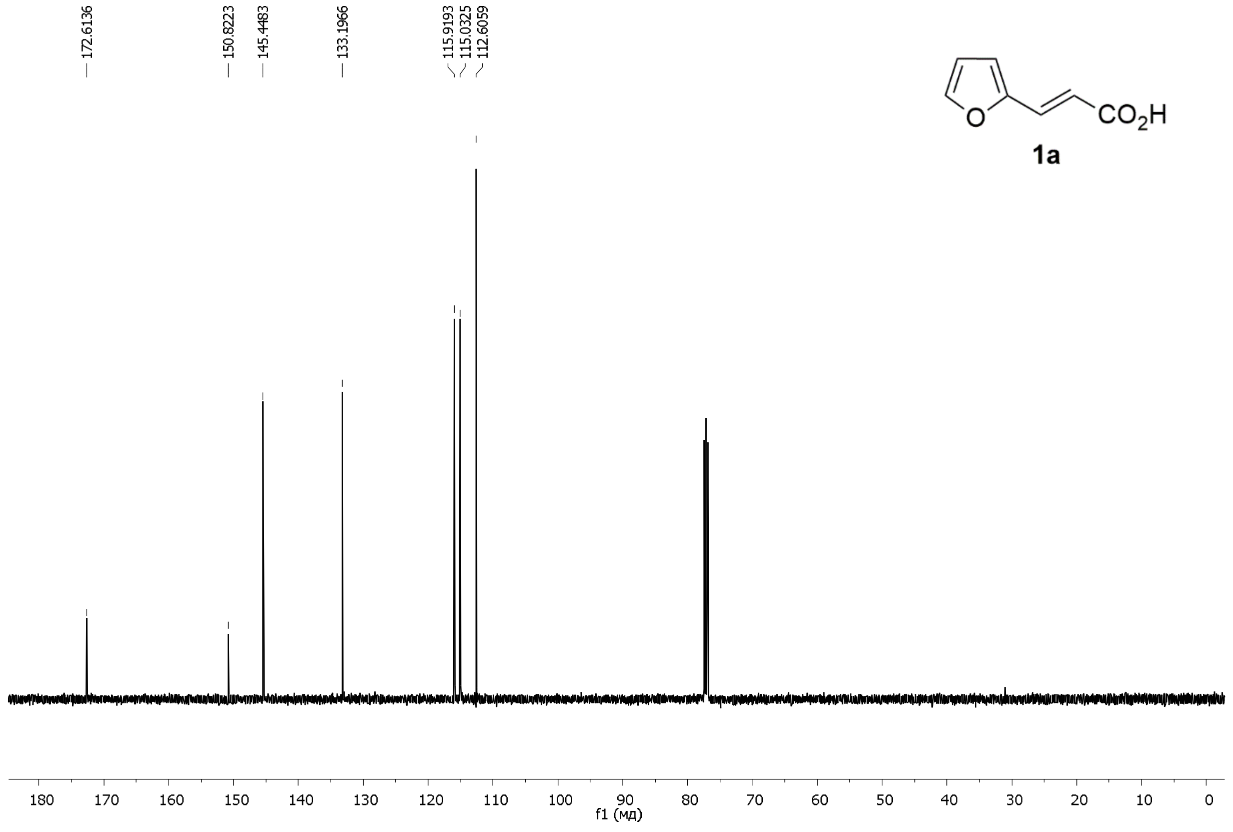


Figure S2. $^{13}$C NMR spectrum of compound **1a** (125 MHz, $CDCl_3$).

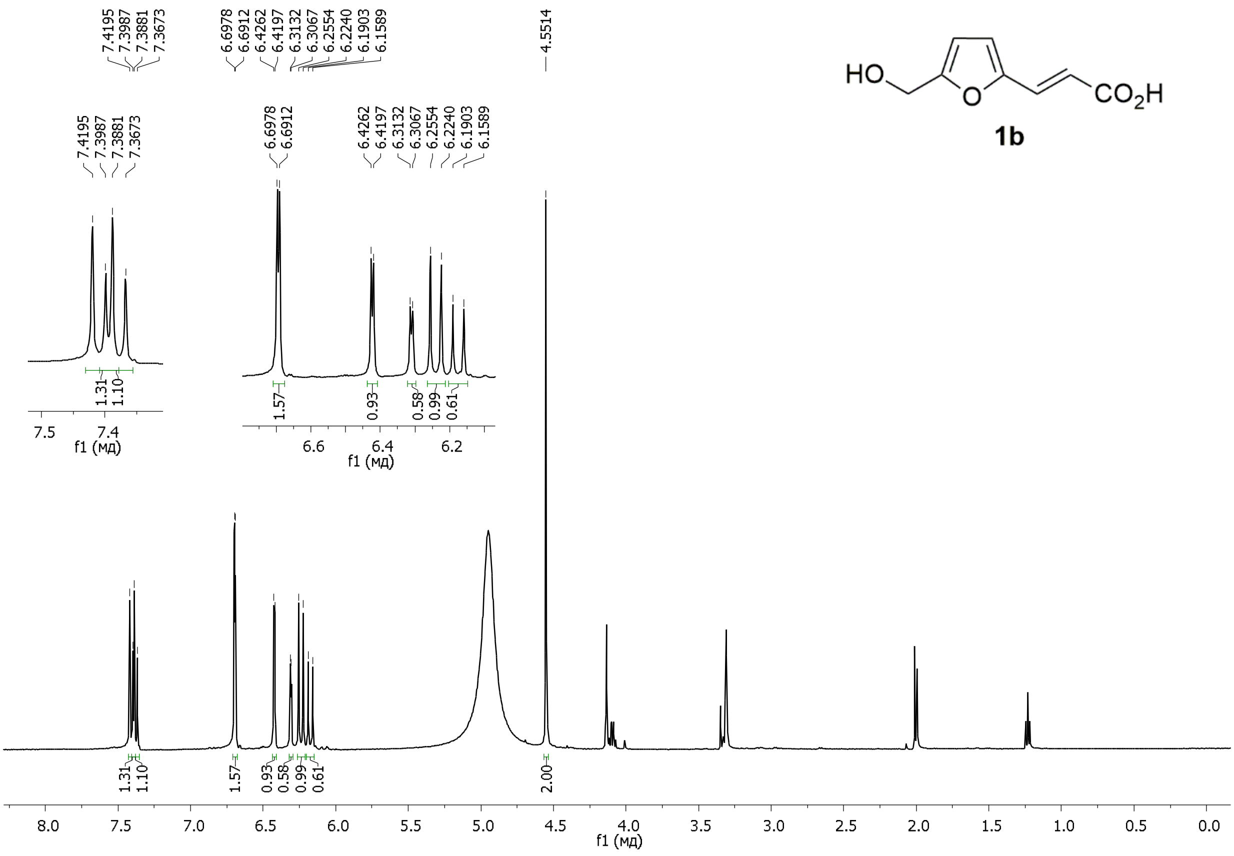


Figure S3. $^{1}$H NMR spectrum of compounds E-/Z-**1b** (500 MHz, $CD_3OD$).

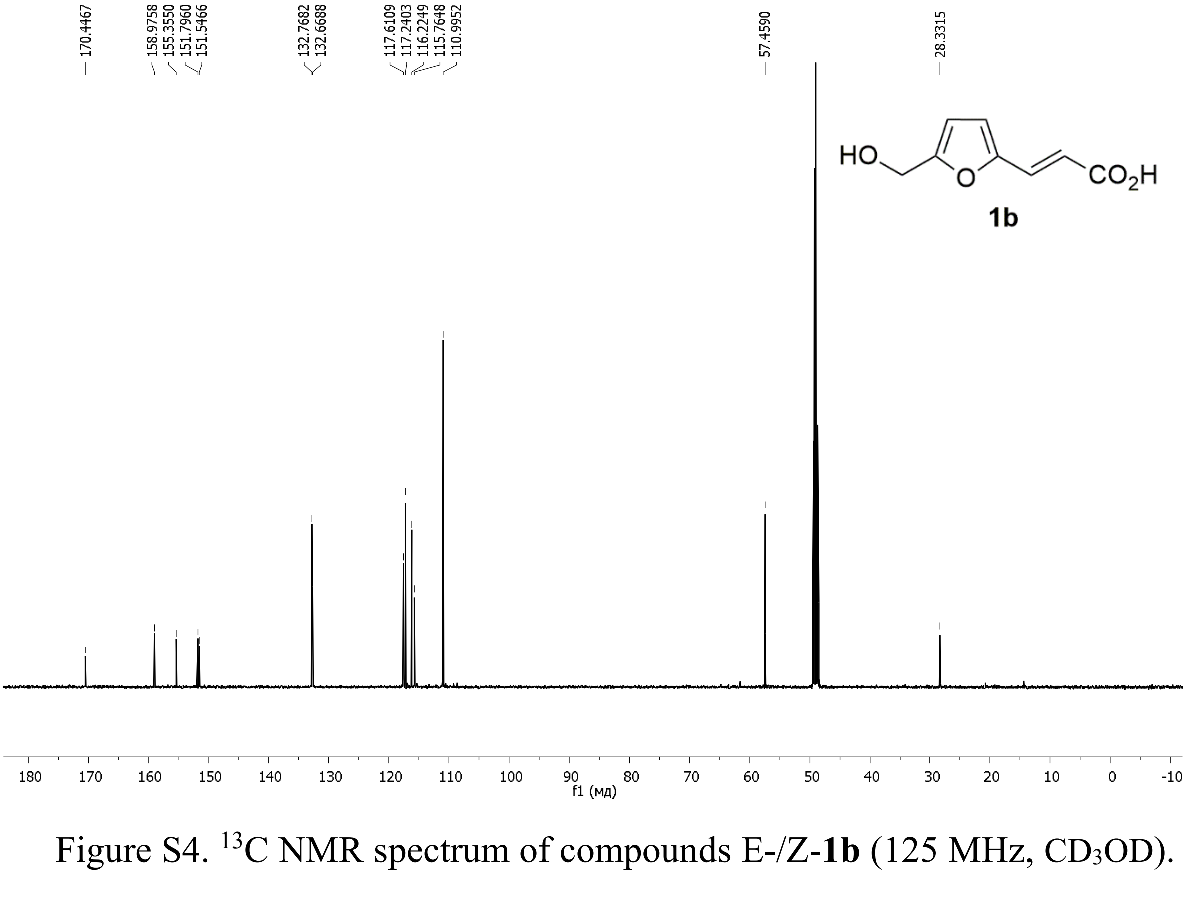


Figure S4. $^{13}$C NMR spectrum of compounds E-/Z-**1b** (125 MHz, $CD_3OD$).

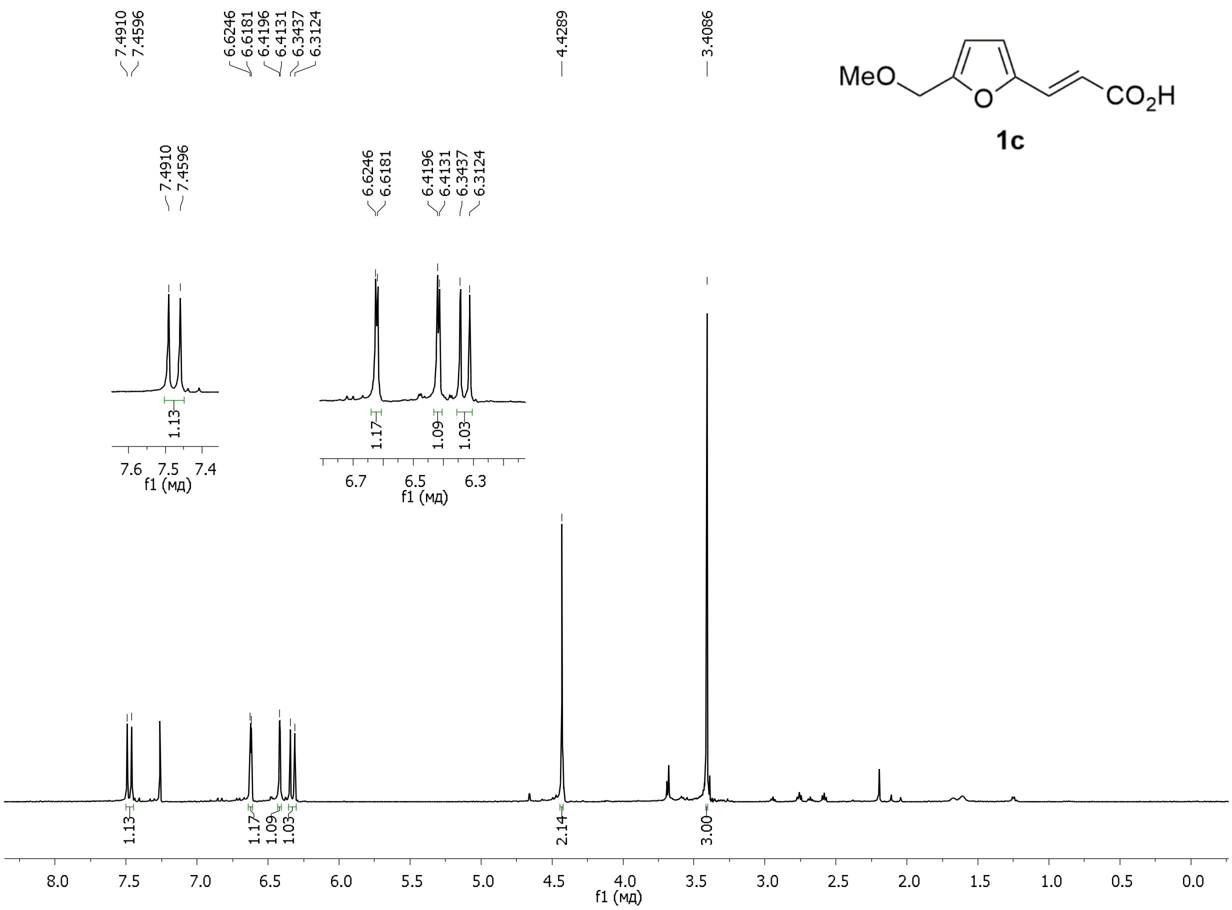


Figure S5. $^1$H NMR spectrum of compound **1c** (500 MHz, $CDCl_3$).

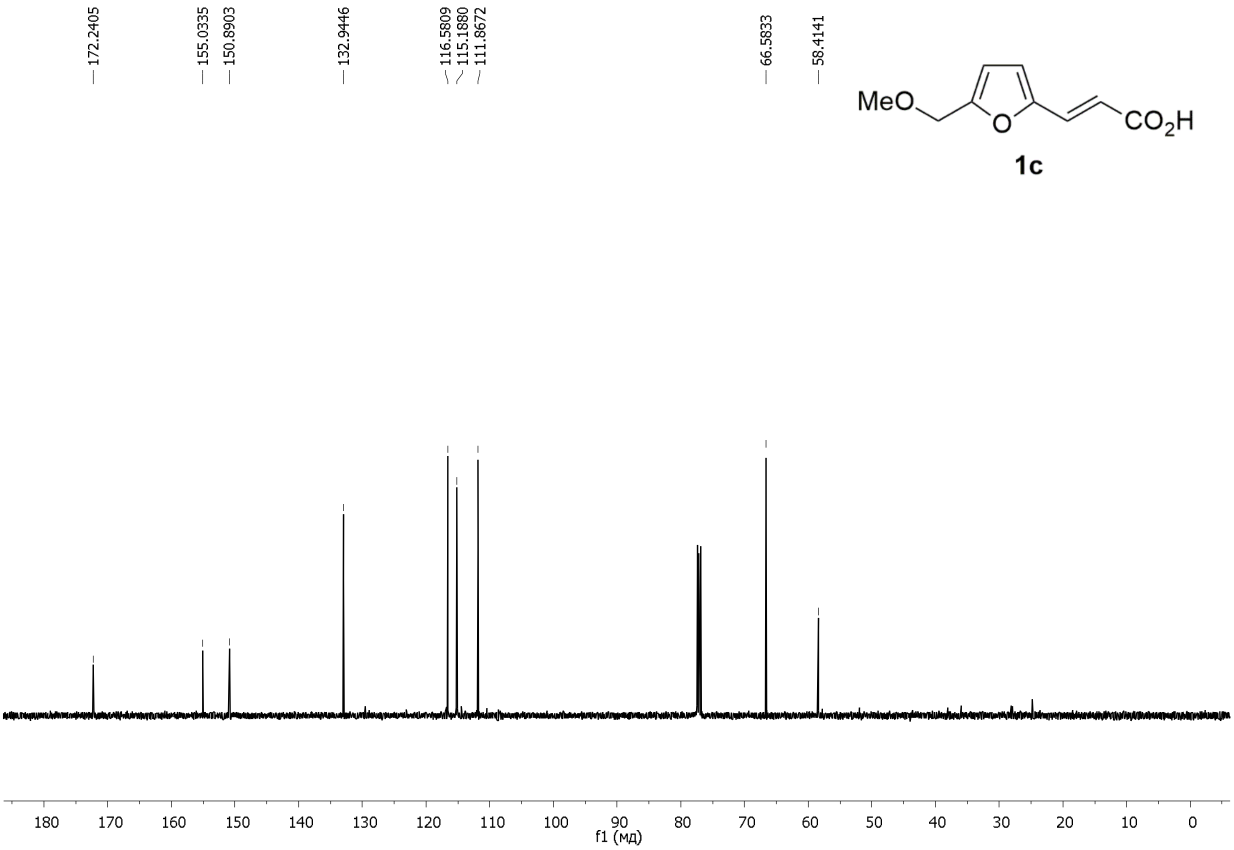


Figure S6. $^{13}$C NMR spectrum of compound **1c** (125 MHz, $CDCl_3$).

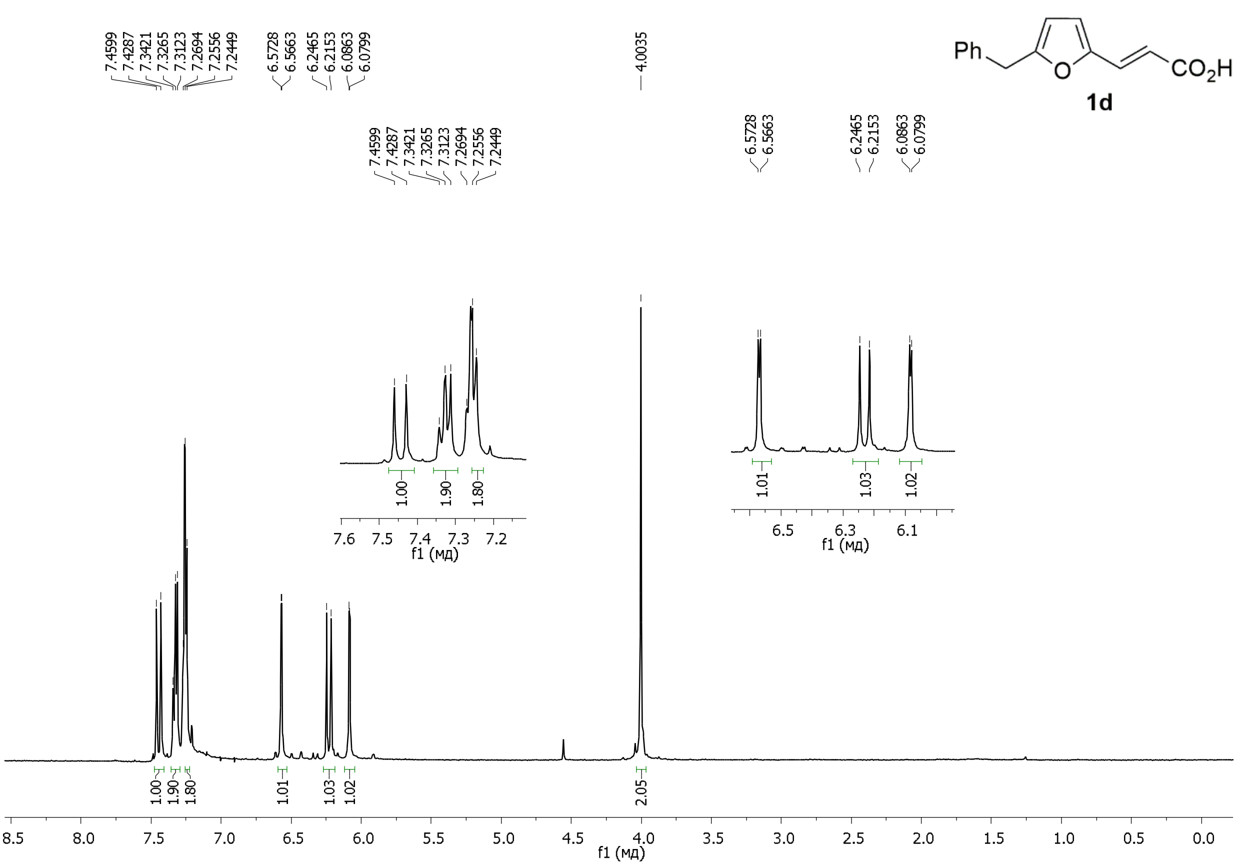


Figure S7. $^{1}H$ NMR spectrum of compound **1d** (500 MHz, $CDCl_3$).

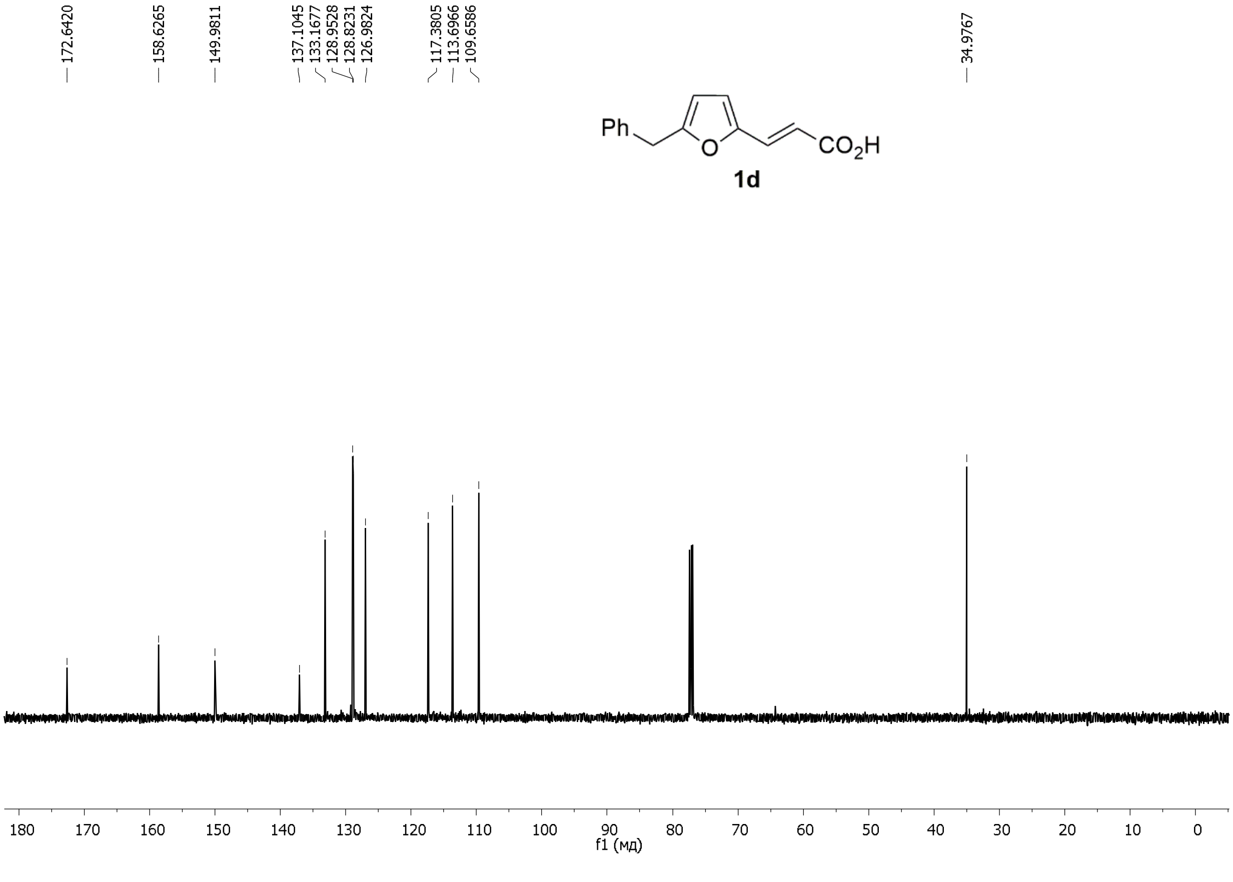


Figure S8. $^{13}C$ NMR spectrum of compound **1d** (125 MHz, $CDCl_3$).

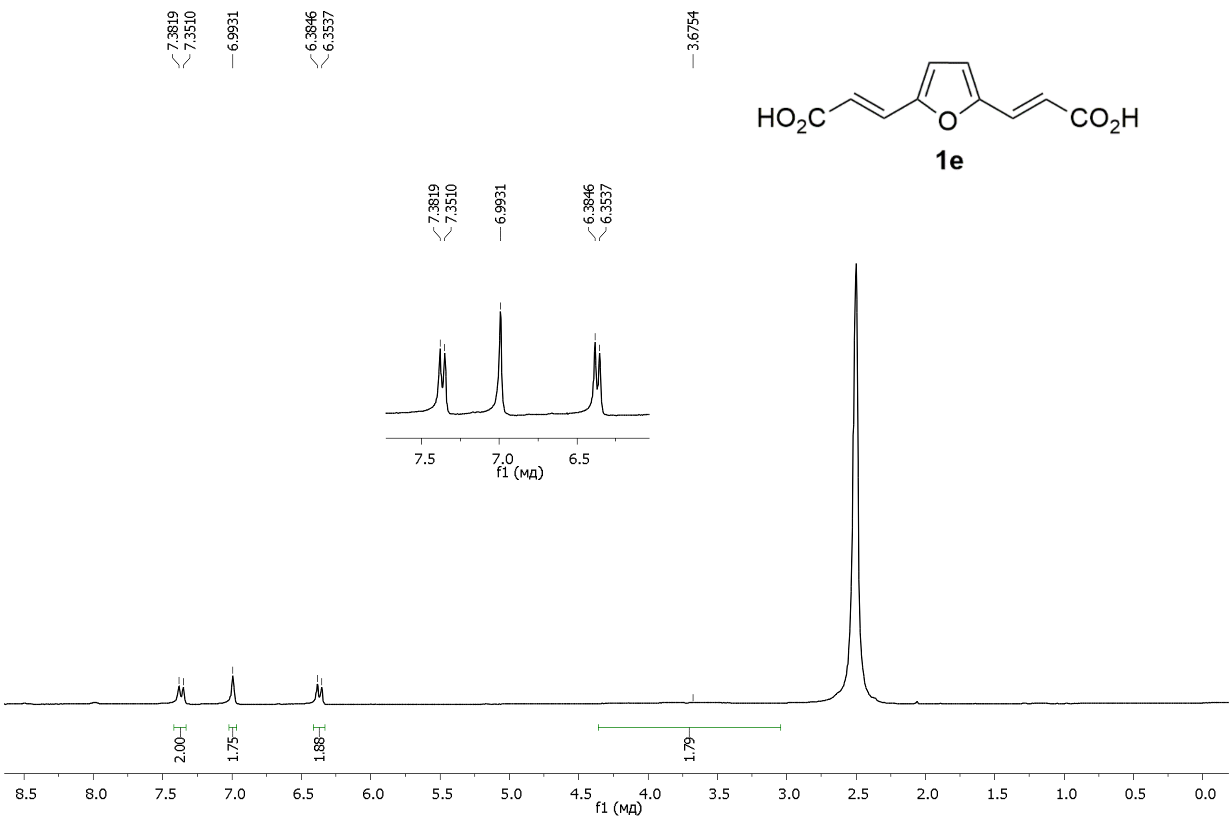


Figure S9. $^{1}H$ NMR spectrum of compound **1e** (500 Hz, $(CD_3)_2SO$)

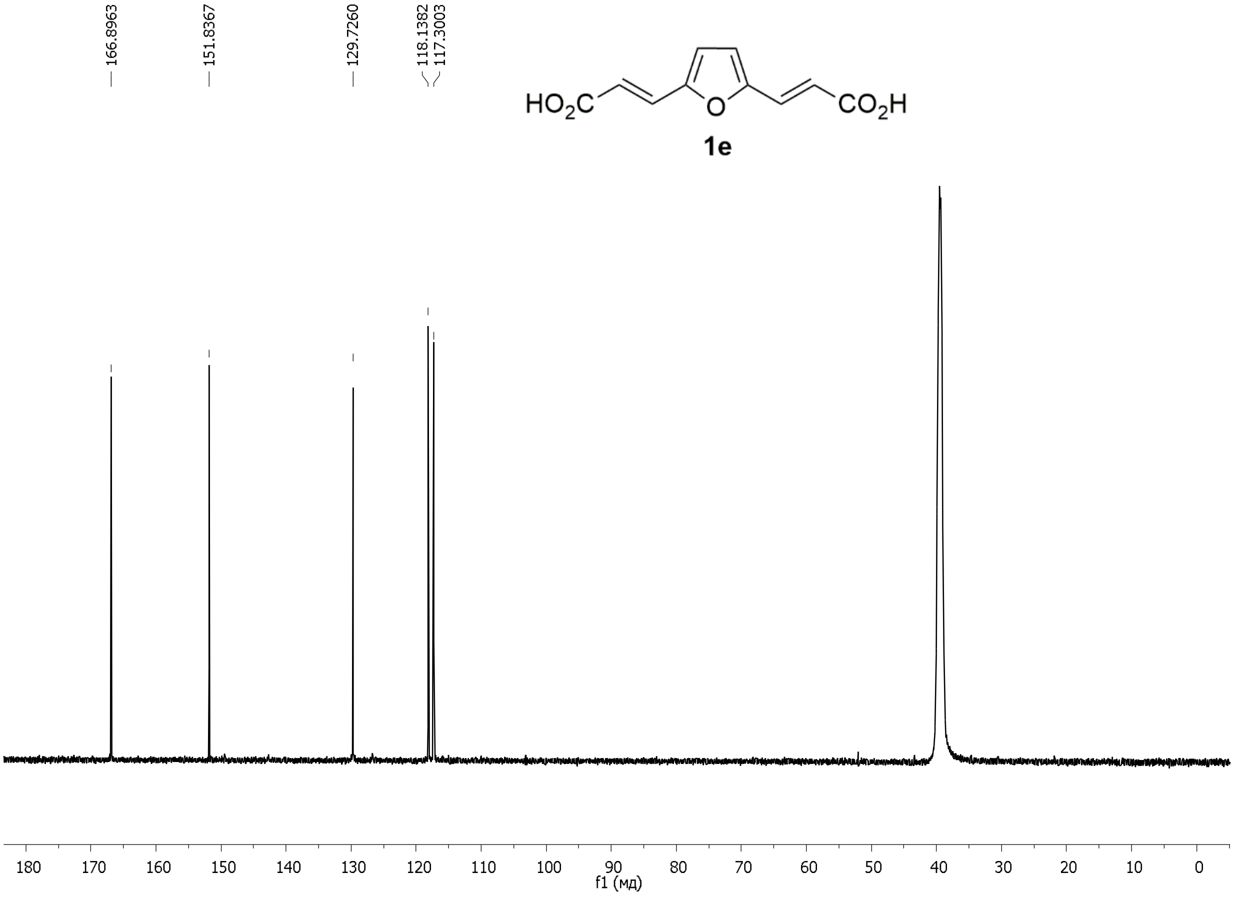


Figure S10. $^{13}C$ NMR spectrum of compound **1e** (125 Hz, $(CD_3)_2SO$)

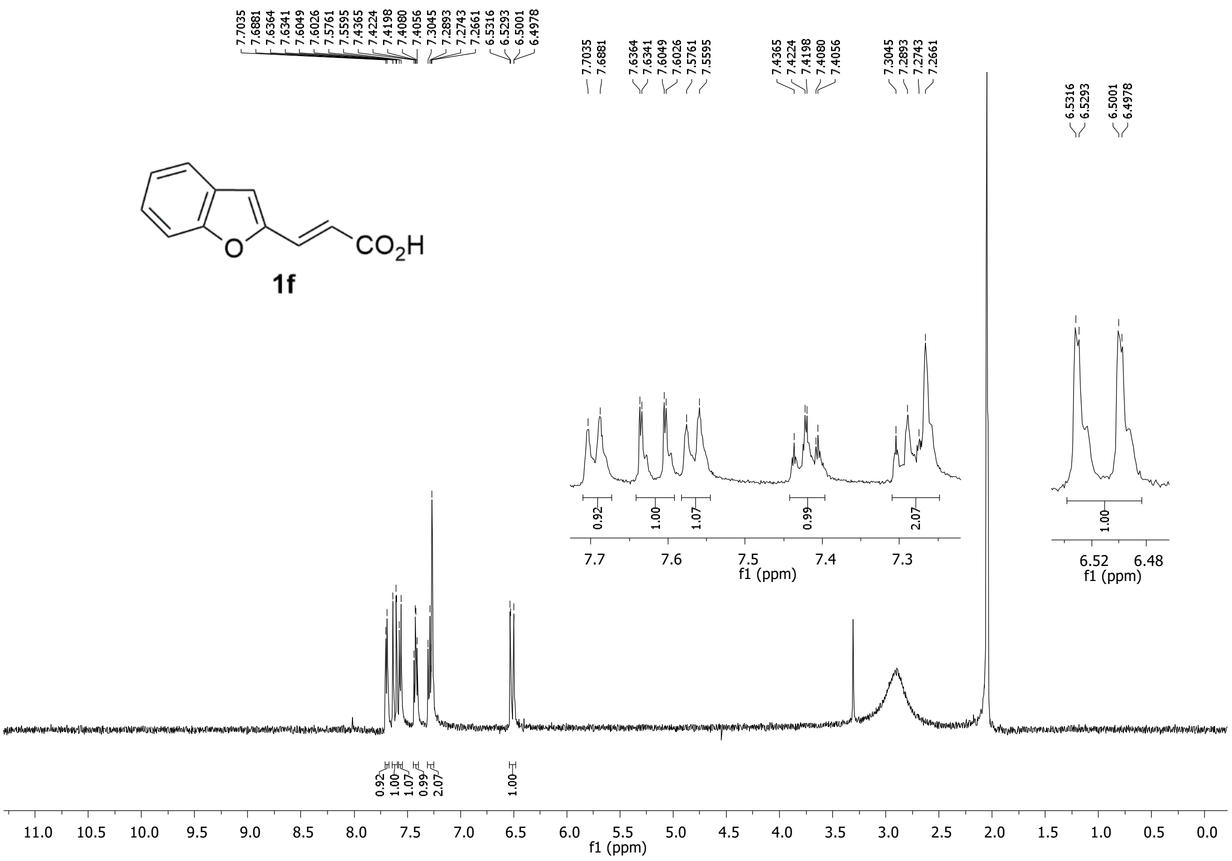

Figure S11. $^{1}$H NMR spectrum of compound **1f** (500 Hz, $(CD_3)_2CO$)

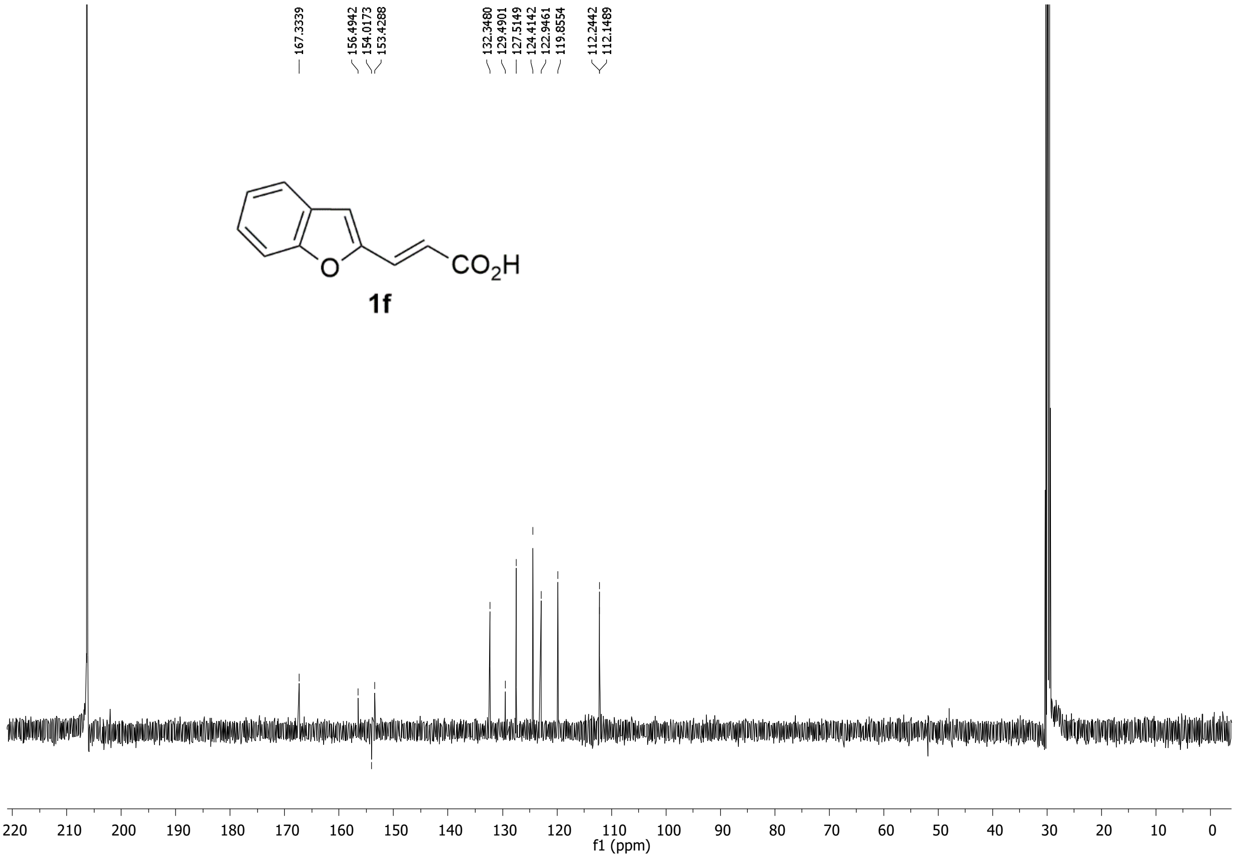

Figure S12. $^{13}$C NMR spectrum of compound **1f** (125 Hz, $(CD_3)_2SO$)

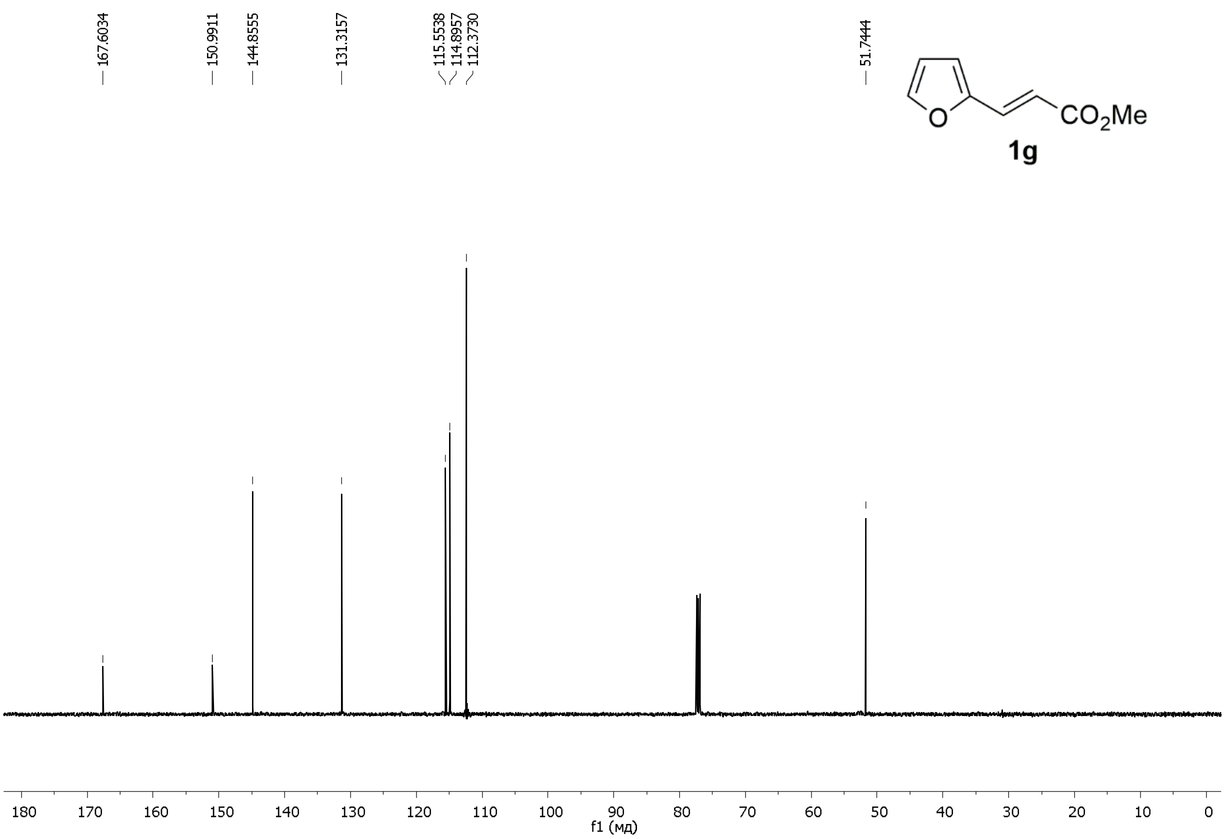


Figure S13. $^{1}$H NMR spectrum of compound **1g** (500 MHz, $CDCl_3$).

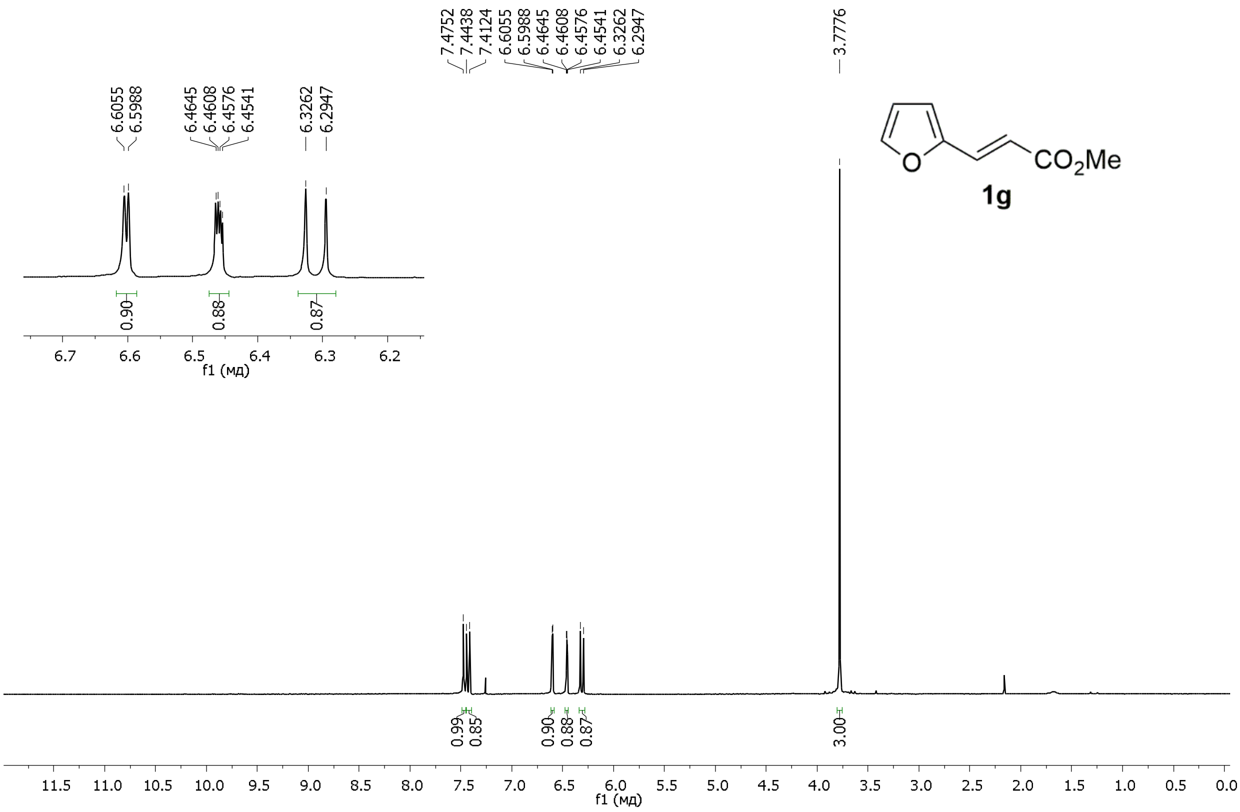


Figure S14. $^{13}$C NMR spectrum of compound **1g** (125 MHz, $CDCl_3$).

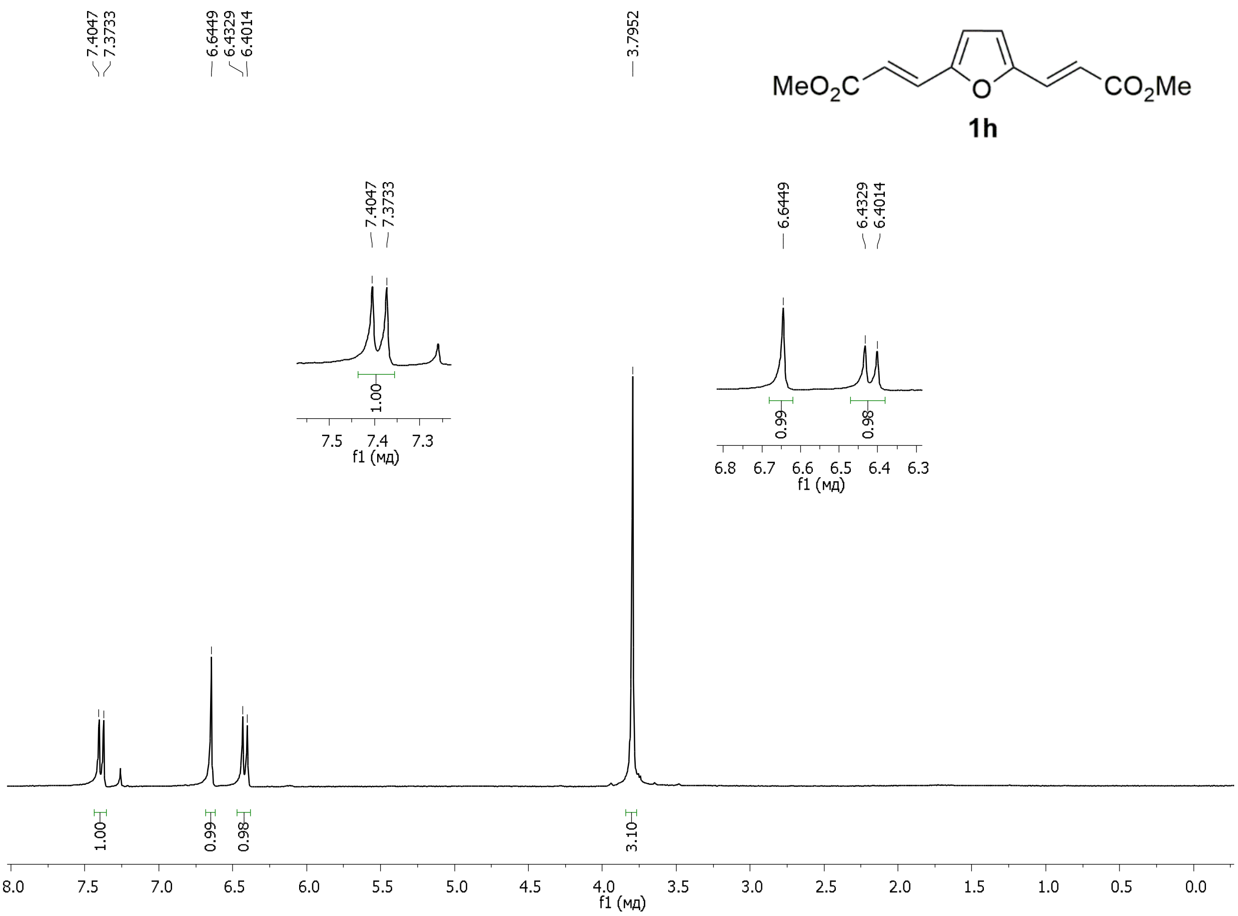


Figure S15. $^{1}$H NMR spectrum of compound **1h** (500 MHz, $CDCl_3$).

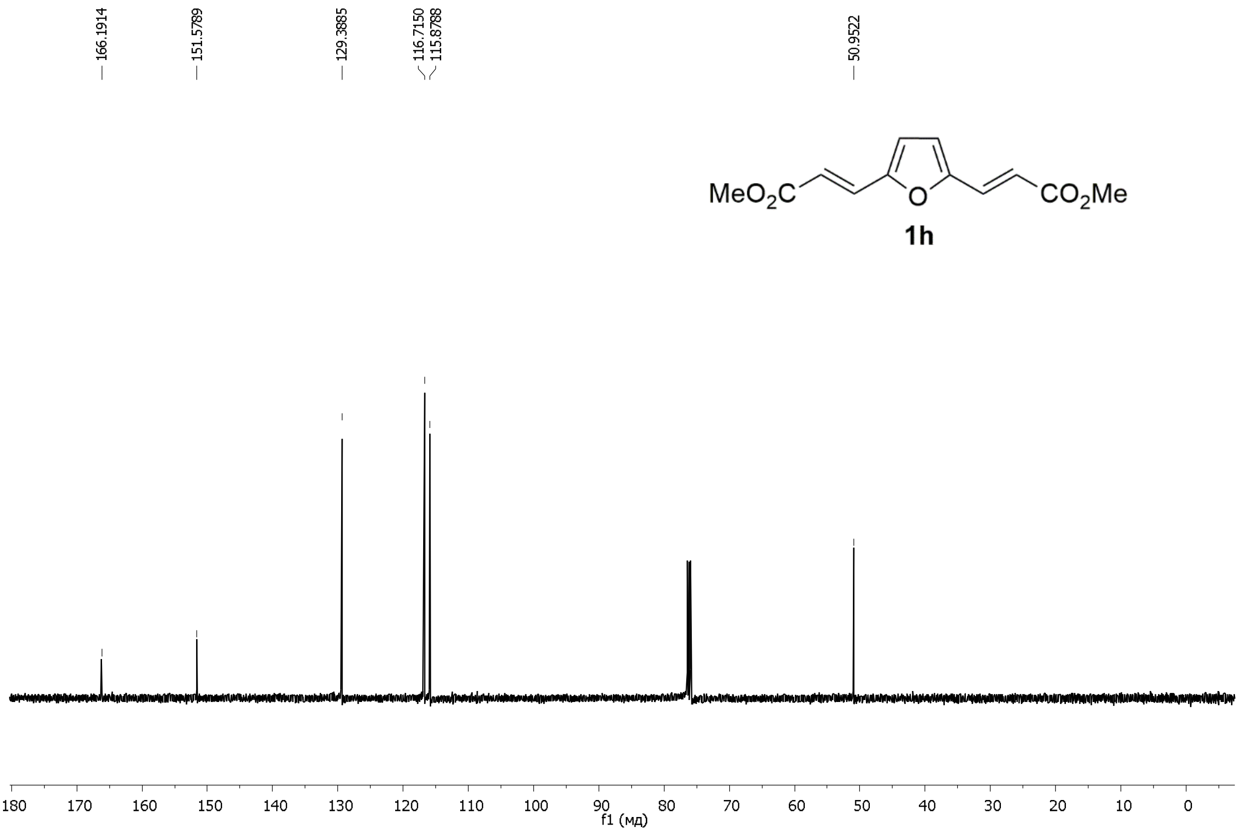


Figure S16. $^{13}$C NMR spectrum of compound **1h** (125 MHz, $CDCl_3$).

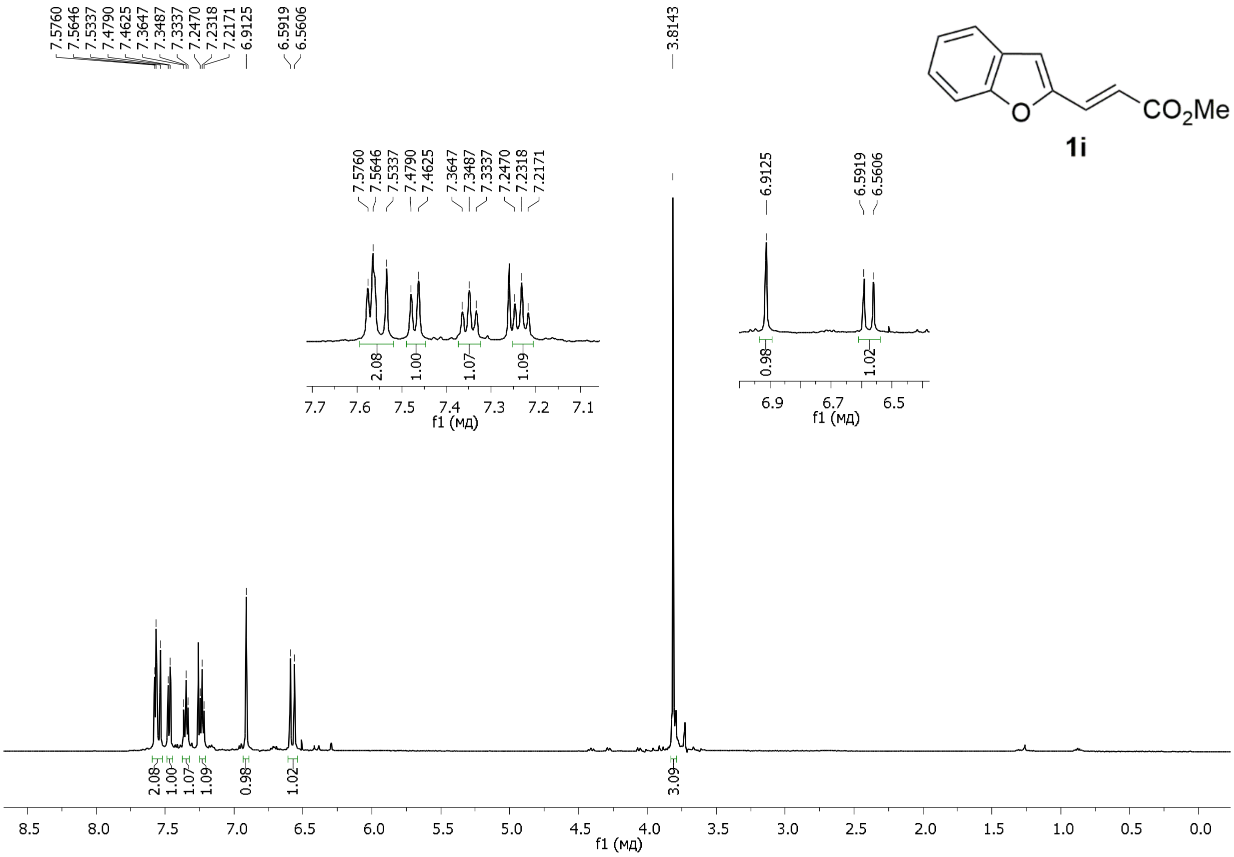


Figure S17. $^{1}$H NMR spectrum of compound **1i** (500 MHz, $CDCl_3$).

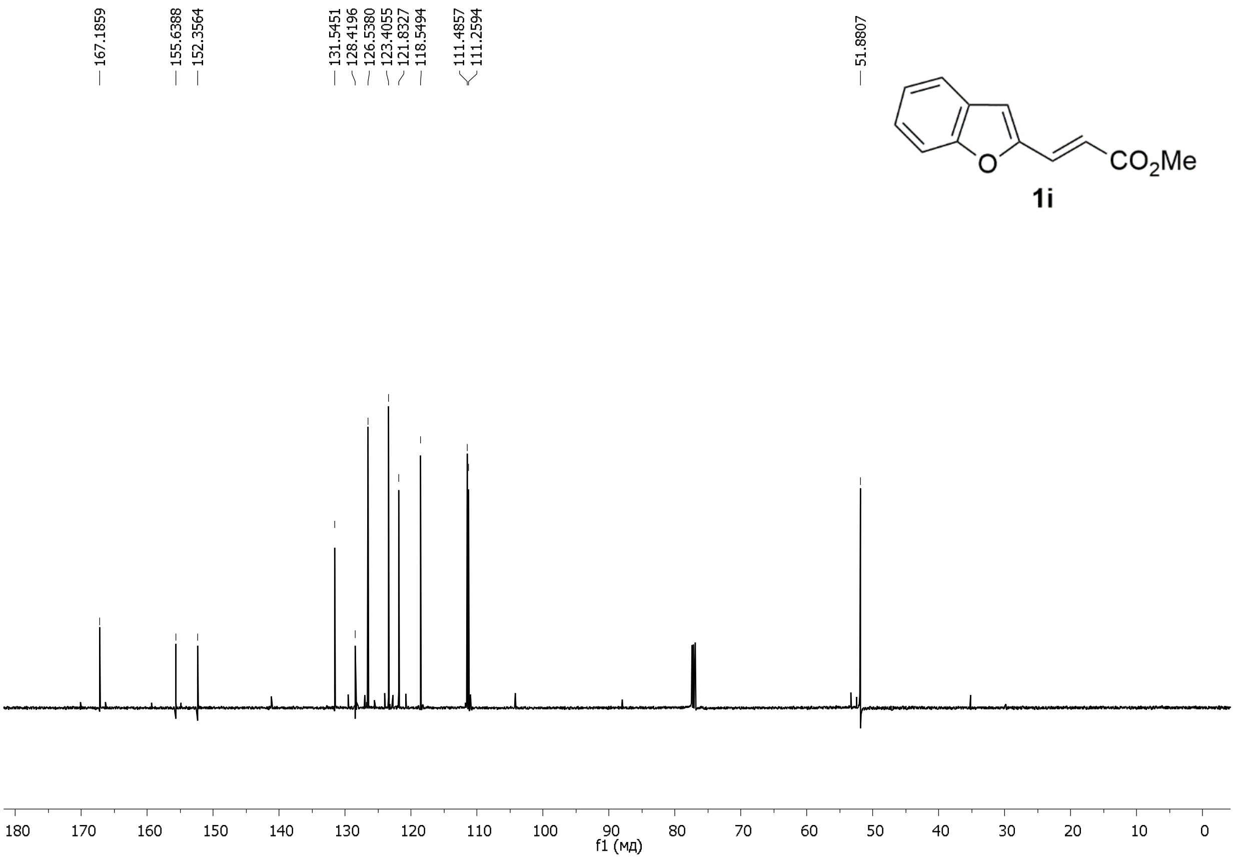


Figure S18. $^{13}$C NMR spectrum of compound **1i** (125 MHz, $CDCl_3$).

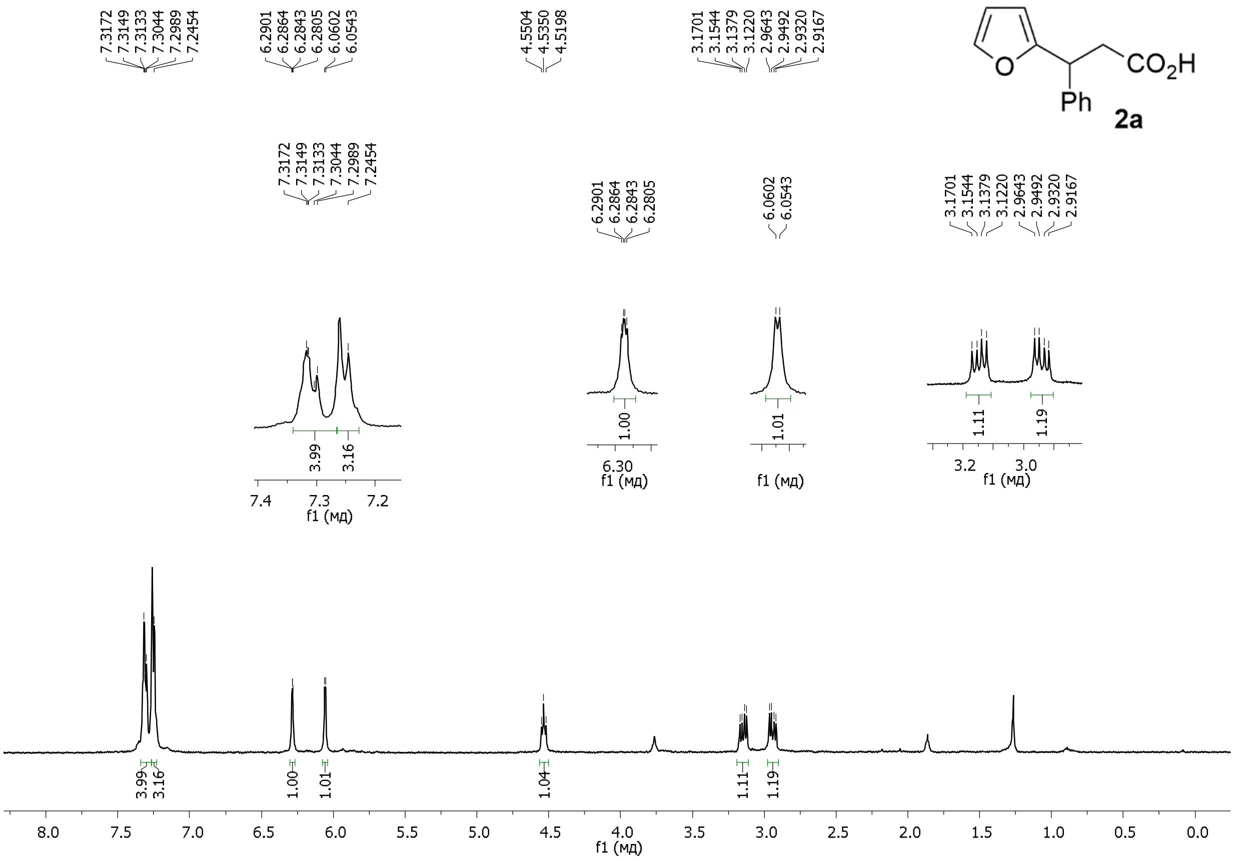


Figure S19. $^{1}$H NMR spectrum of compound **2a** (500 MHz, $CDCl_3$).

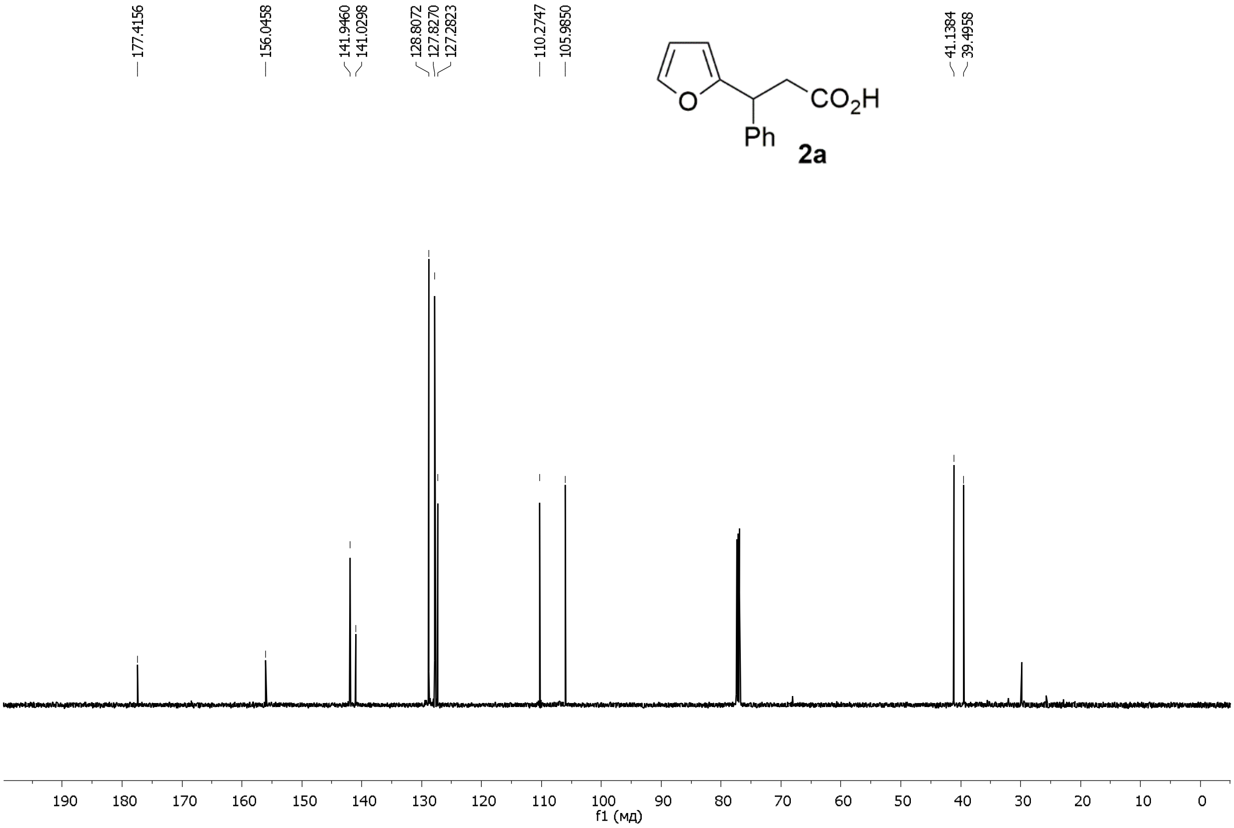


Figure S20. $^{13}$C NMR spectrum of compound **2a** (125 MHz, $CDCl_3$).

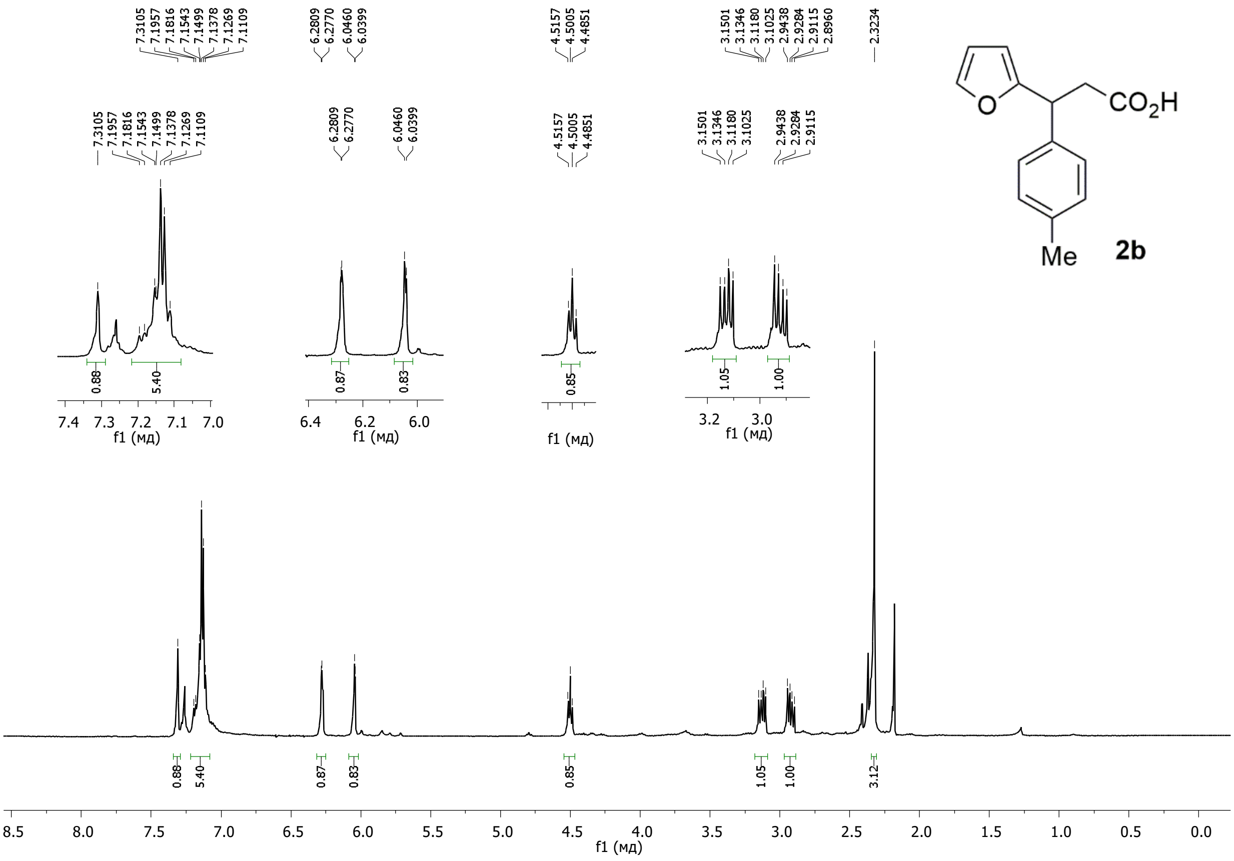

Figure S21. $^{1}$H NMR spectrum of compound **2b** (500 MHz, $CDCl_3$).

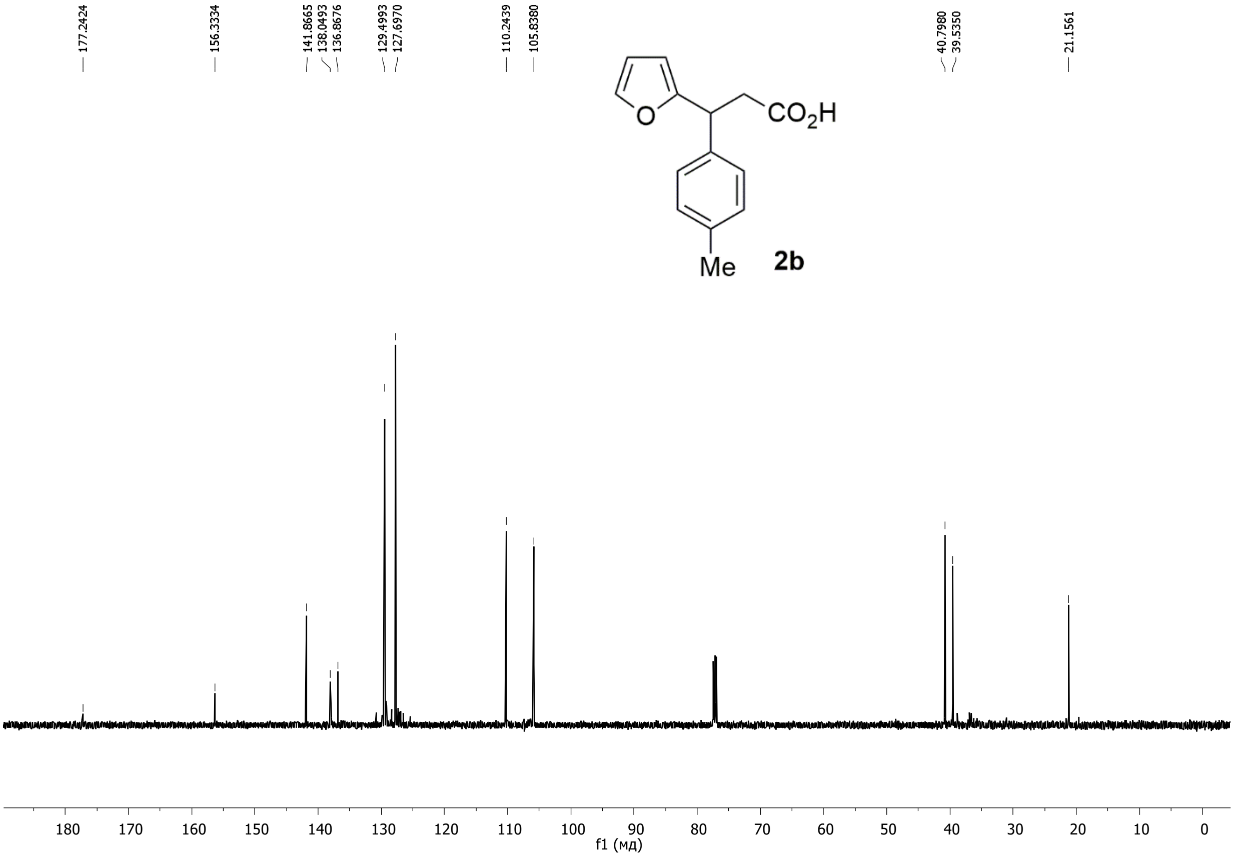

Figure S22. $^{13}$C NMR spectrum of compound **2b** (125 MHz, $CDCl_3$).

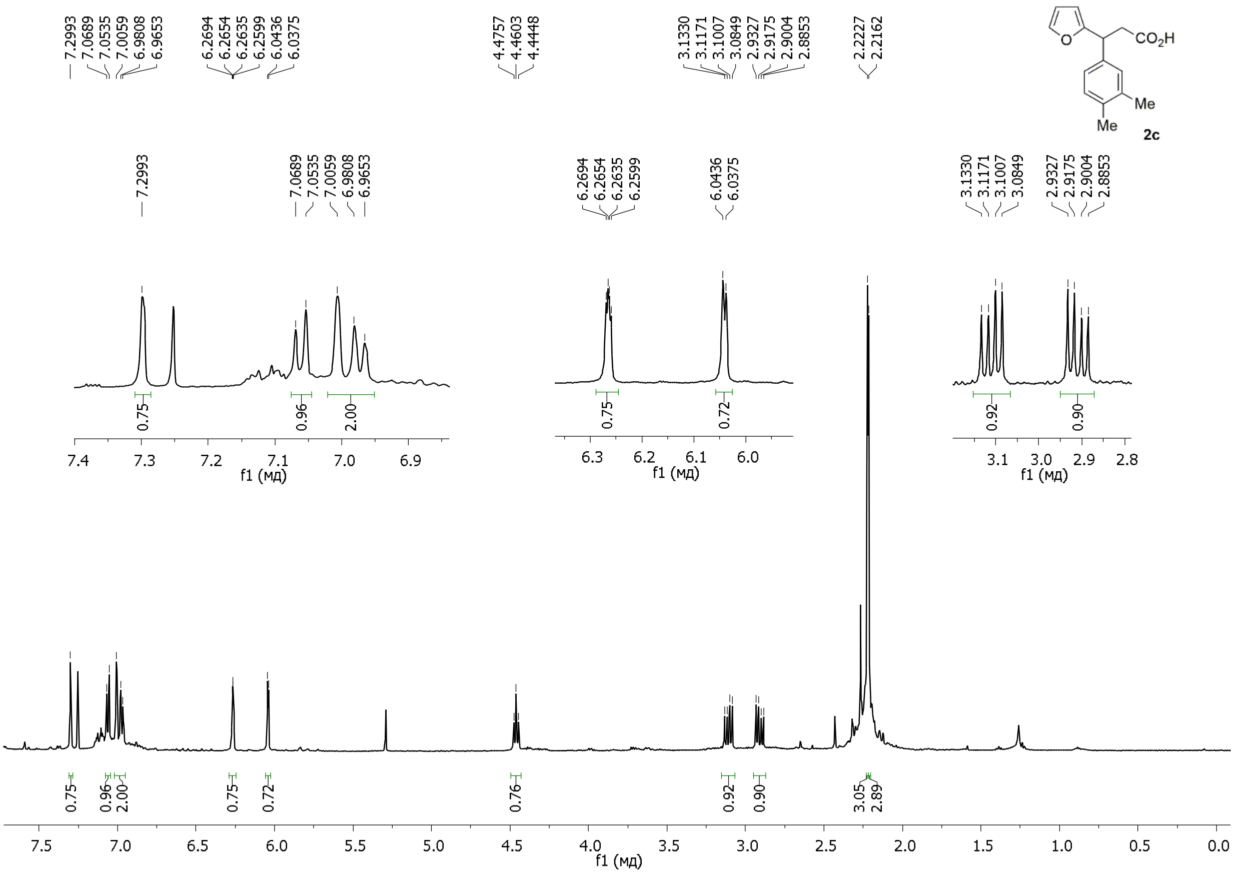


Figure S23. $^{1}$H NMR spectrum of compound **2c** (500 MHz, $CDCl_3$).

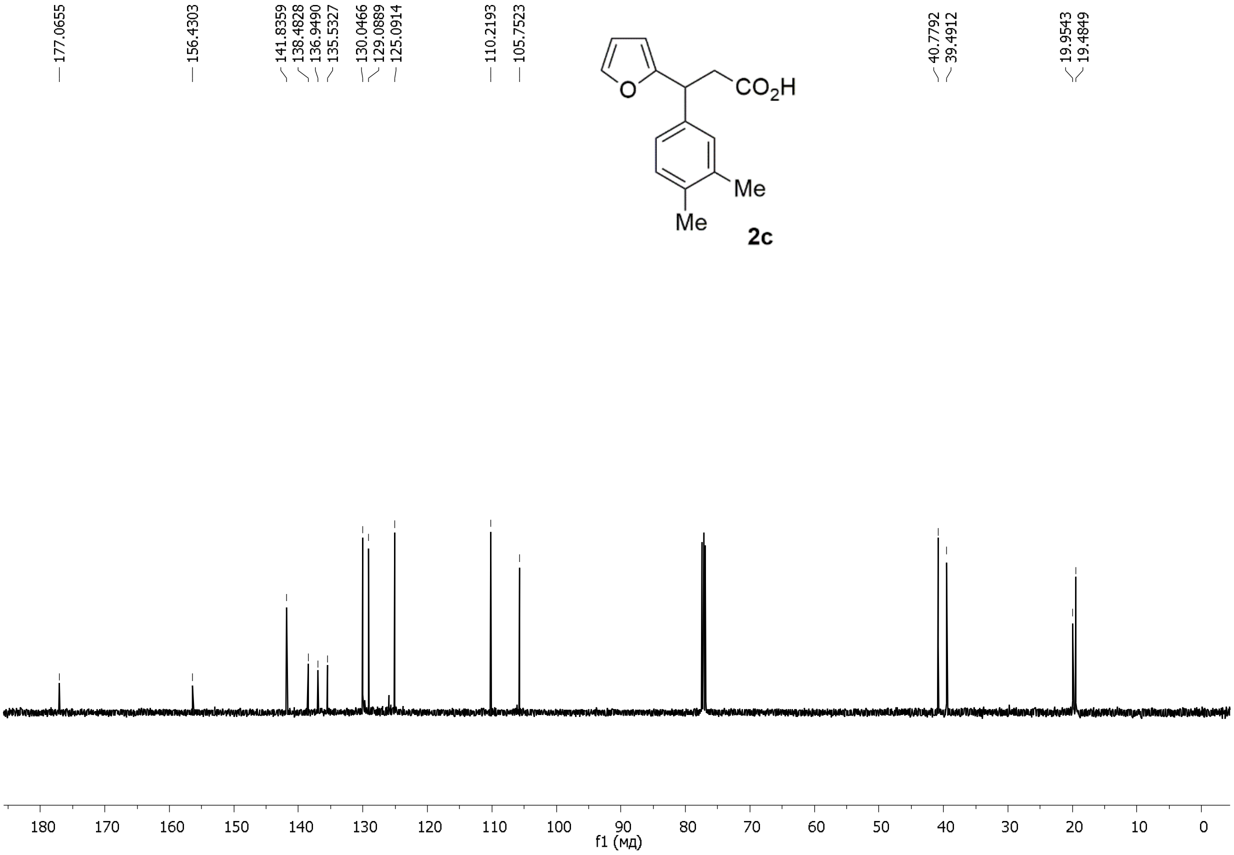

Figure S24. $^{13}C$ NMR spectrum of compound **2c** (125 MHz, $CDCl_3$).

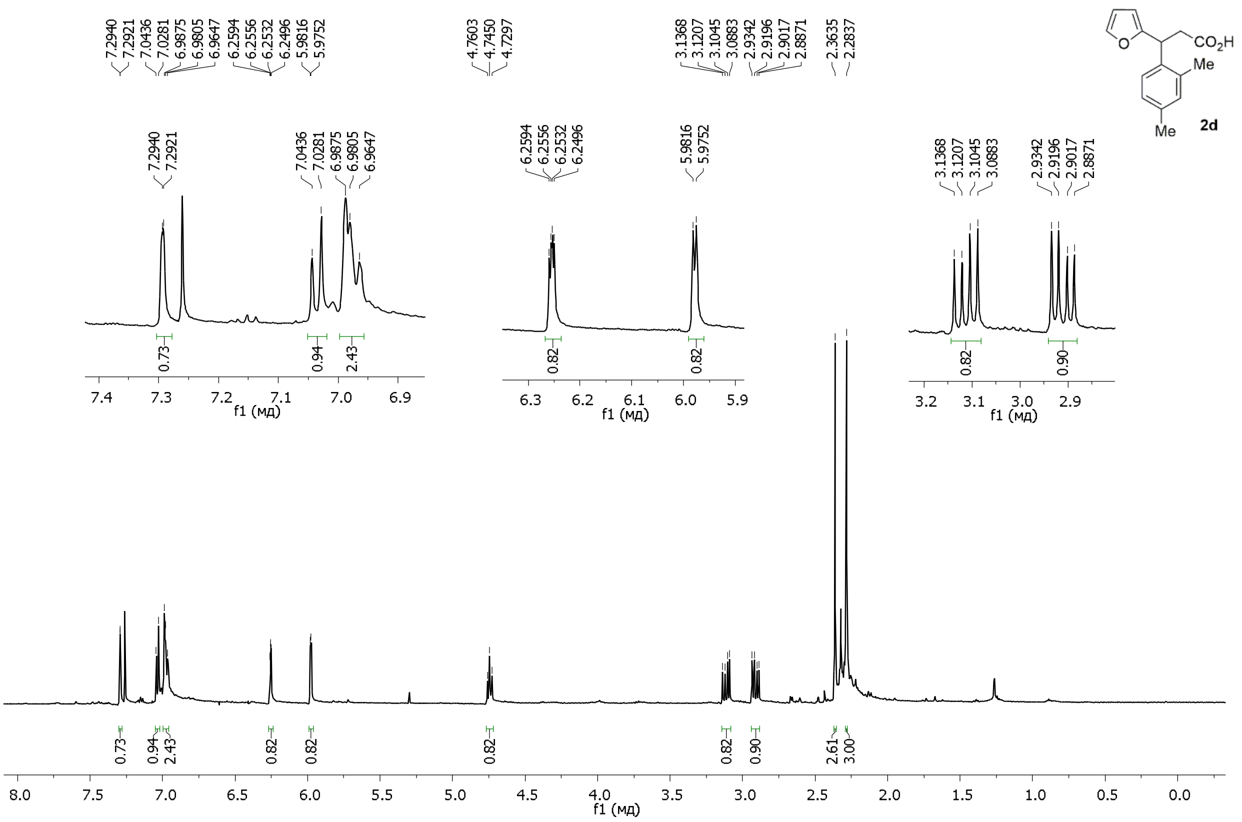


Figure S25. $^{1}H$ NMR spectrum of compound **2d** (500 MHz, $CDCl_3$).

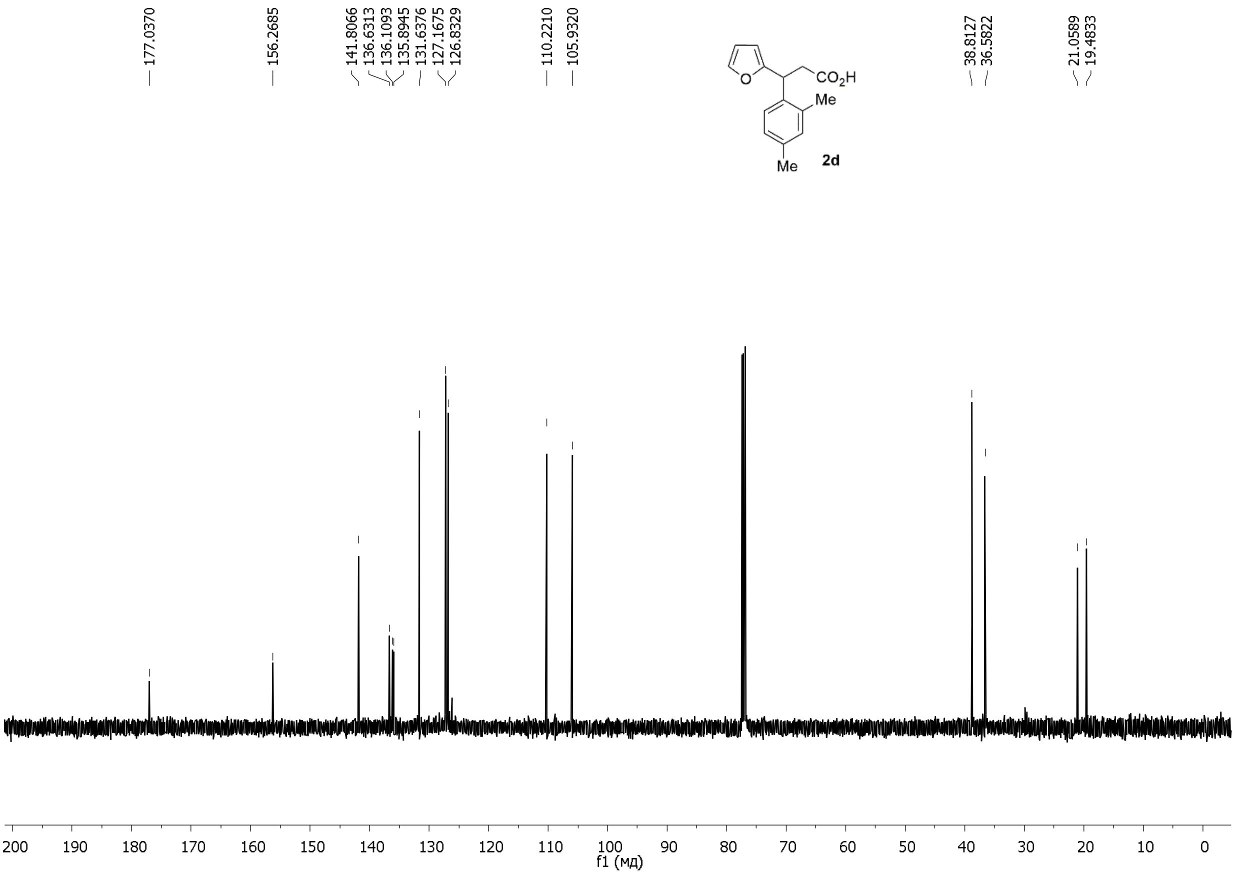


Figure S26. $^{13}C$ NMR spectrum of compound **2d** (125 MHz, $CDCl_3$).

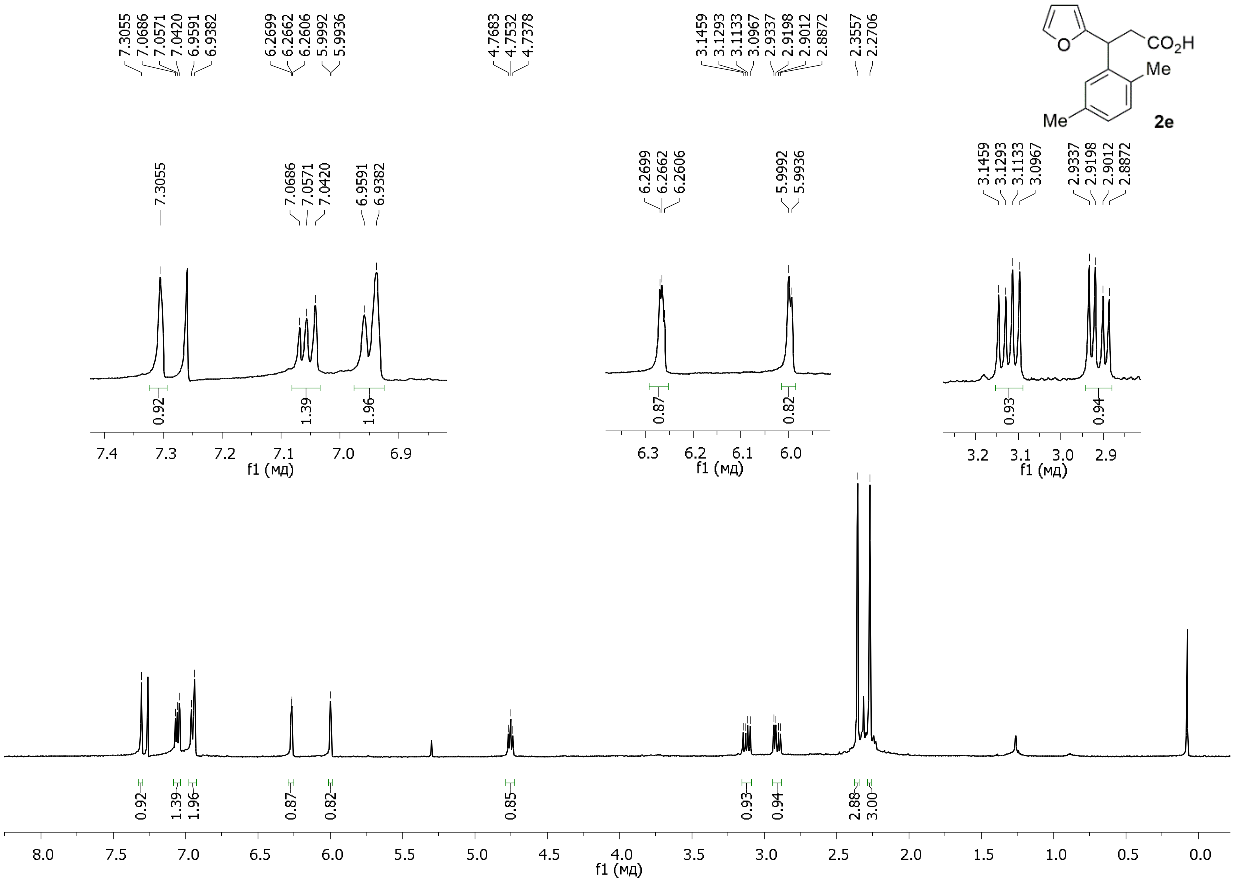


Figure S27. $^{1}H$ NMR spectrum of compound **2e** (500 MHz, $CDCl_3$).

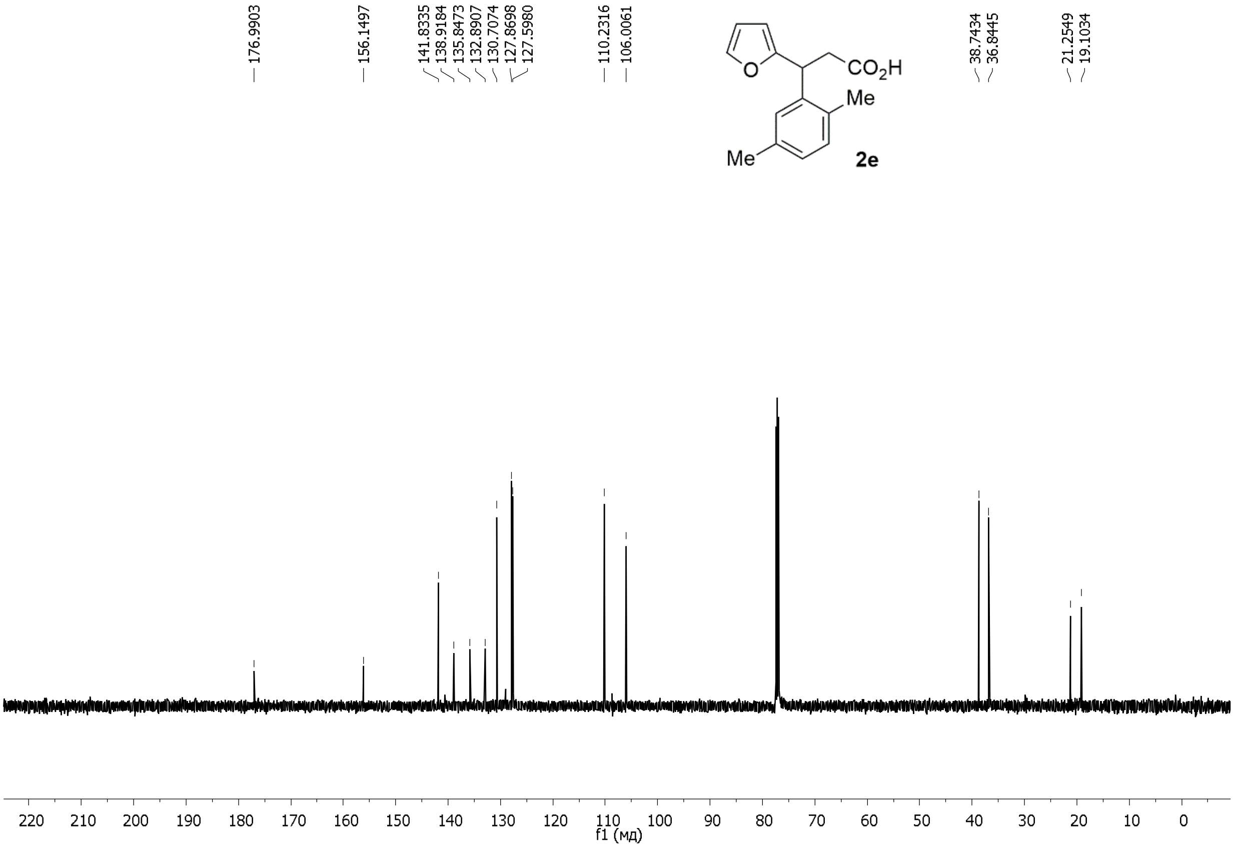


Figure S28. $^{13}C$ NMR spectrum of compound **2e** (125 MHz, $CDCl_3$).

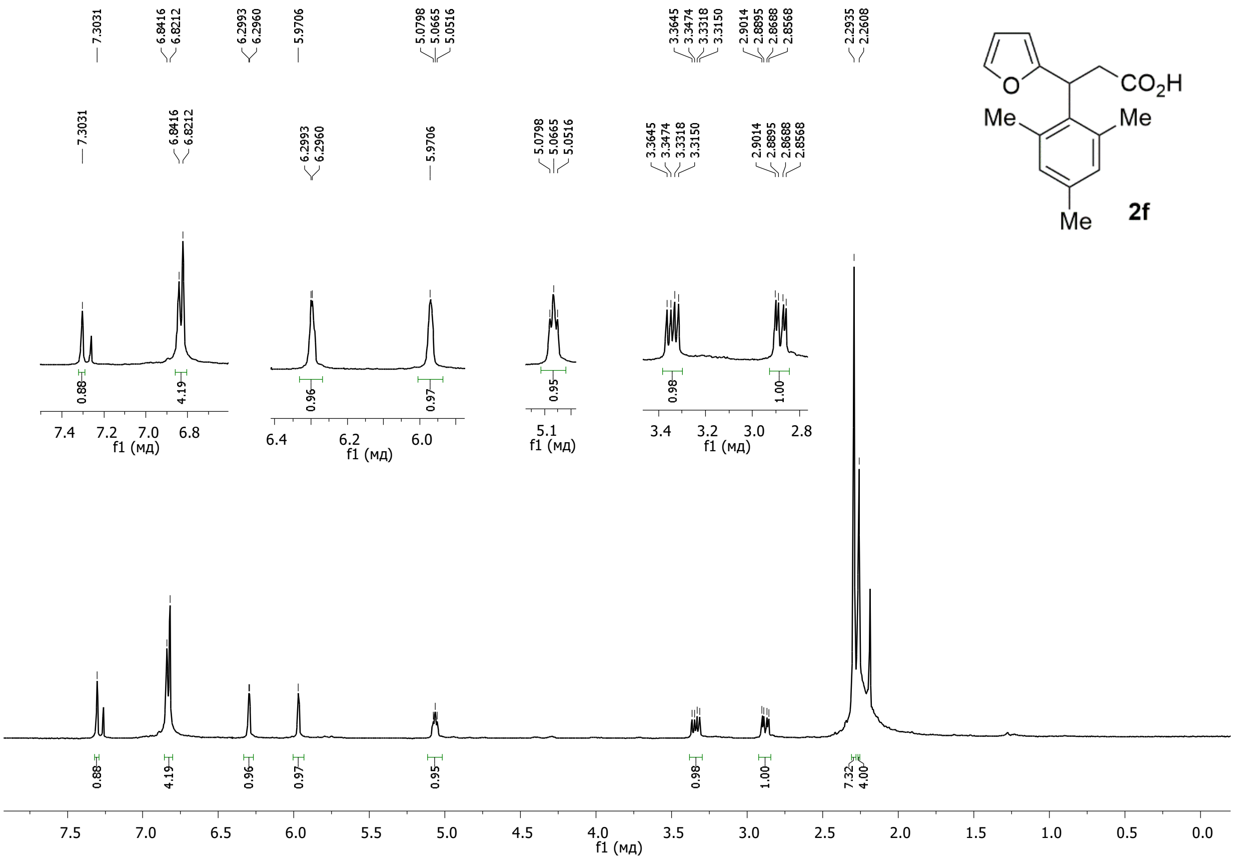


Figure S29. $^{1}$H NMR spectrum of compound **2f** (500 MHz, $CDCl_3$).

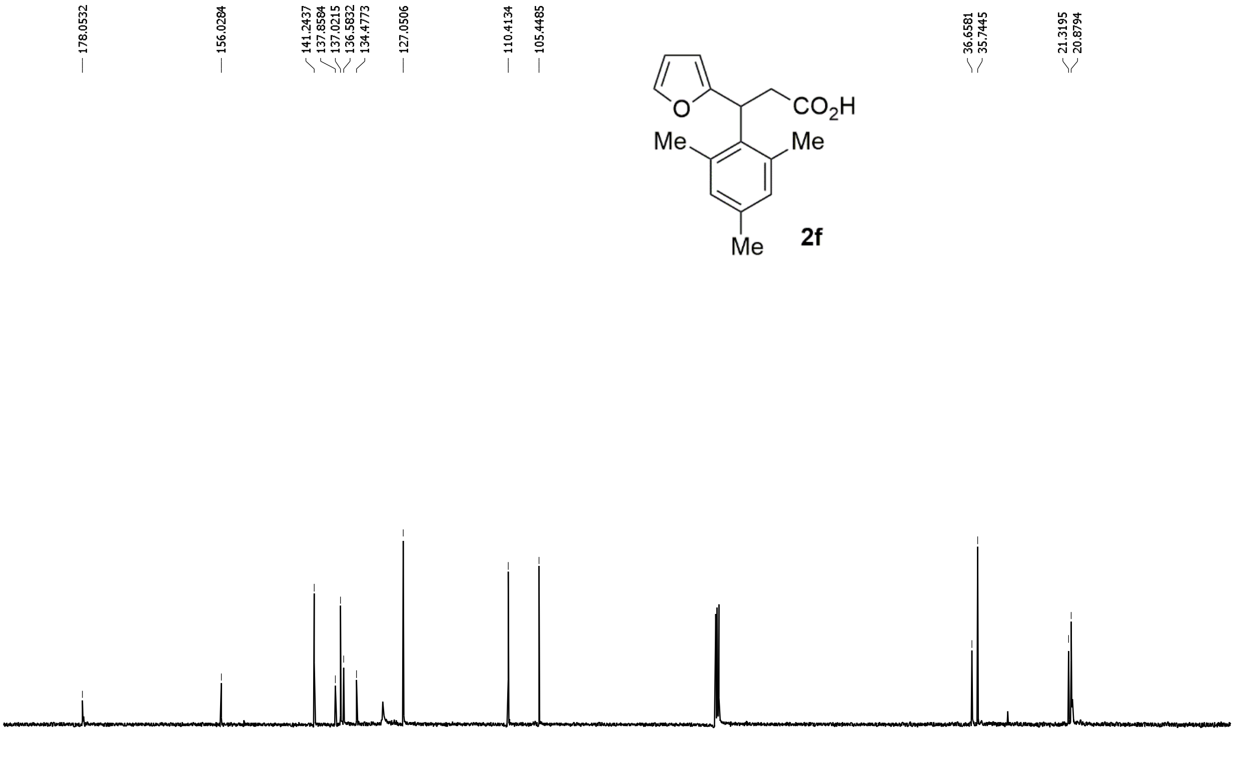


Figure S30. $^{13}$C NMR spectrum of compound **2f** (125 MHz, $CDCl_3$).

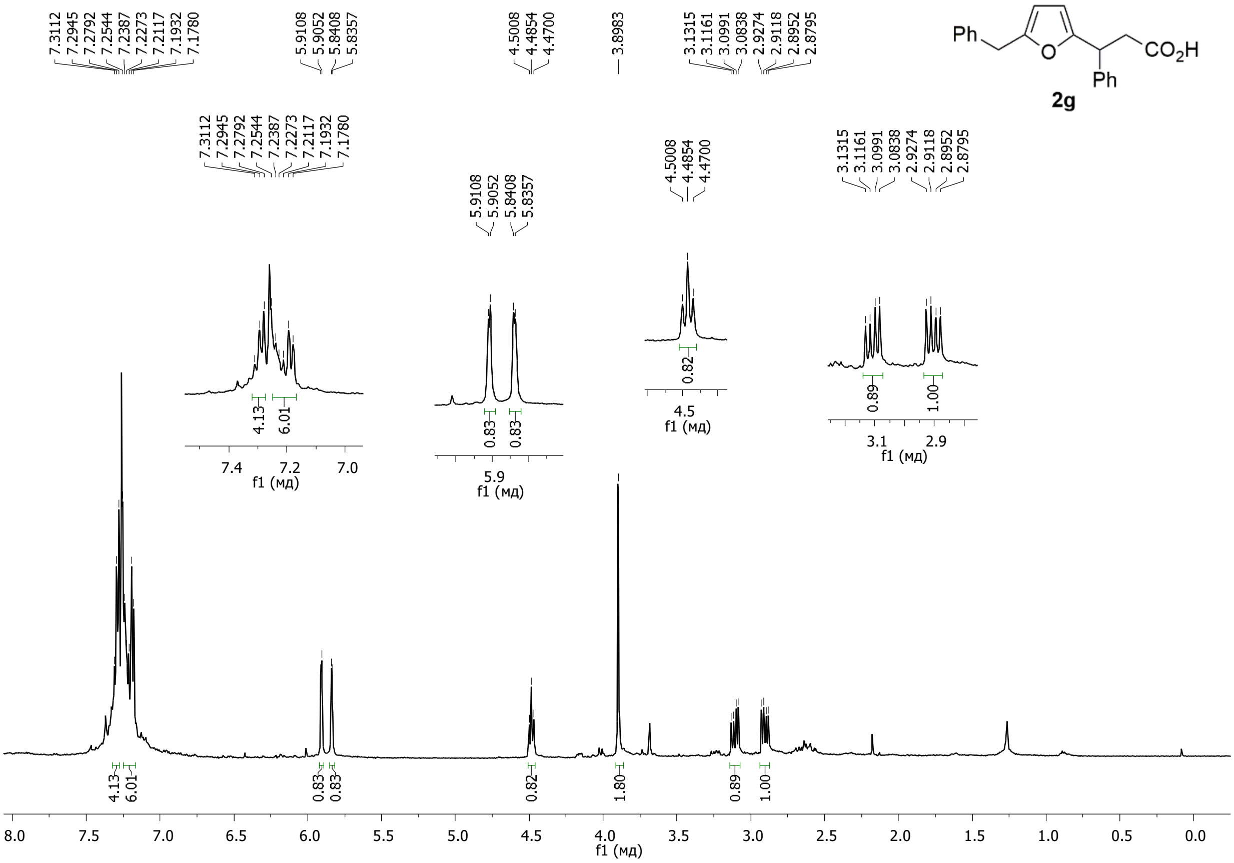

Figure S31. $^{1}H$ NMR spectrum of compound **2g** (500 MHz, $CDCl_3$).

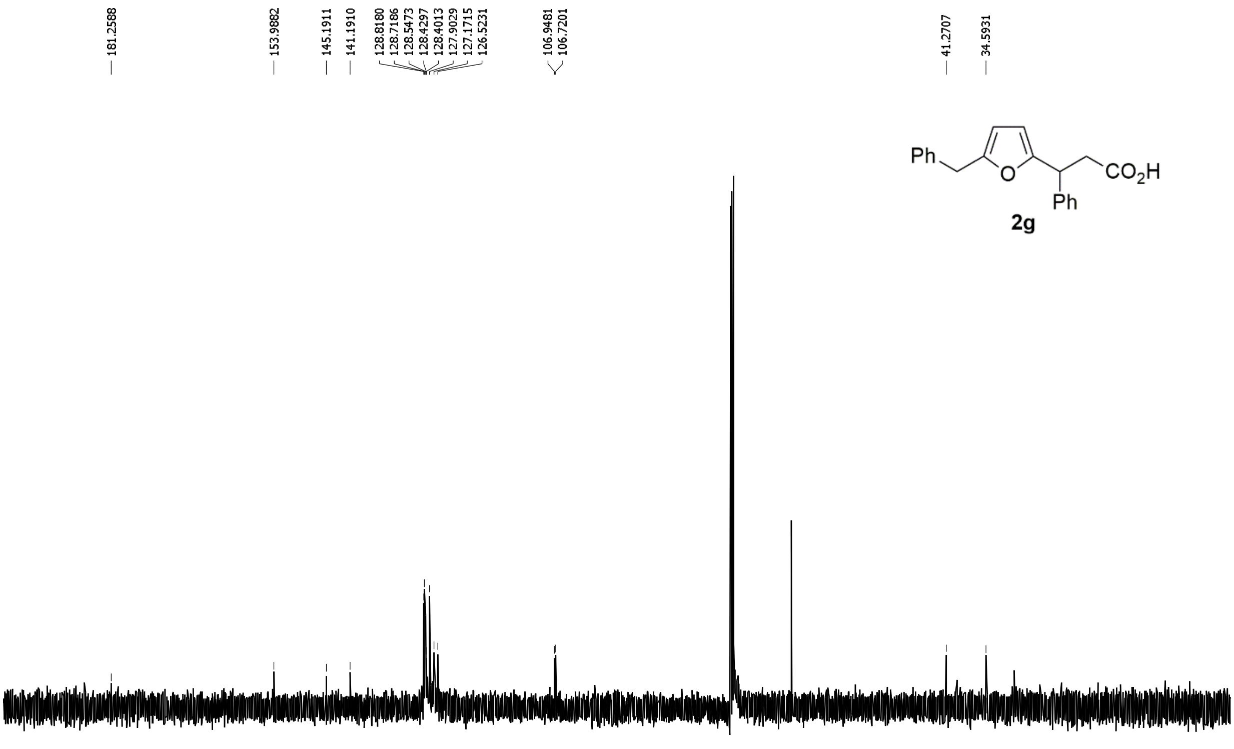

Figure S32. $^{13}C$ NMR spectrum of compound **2g** (125 MHz, $CDCl_3$).

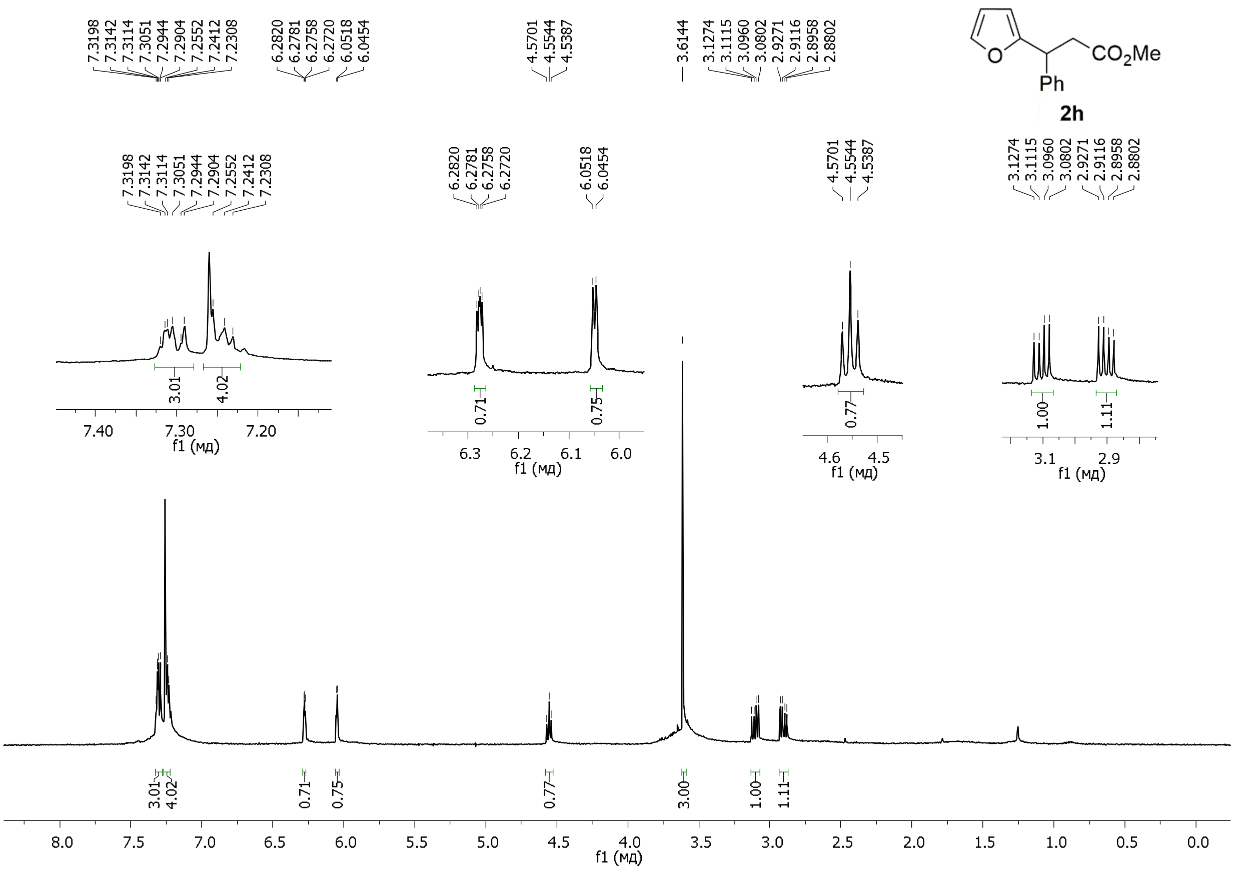


Figure S33. $^{1}$H NMR spectrum of compound **2h** (500 MHz, $CDCl_3$).

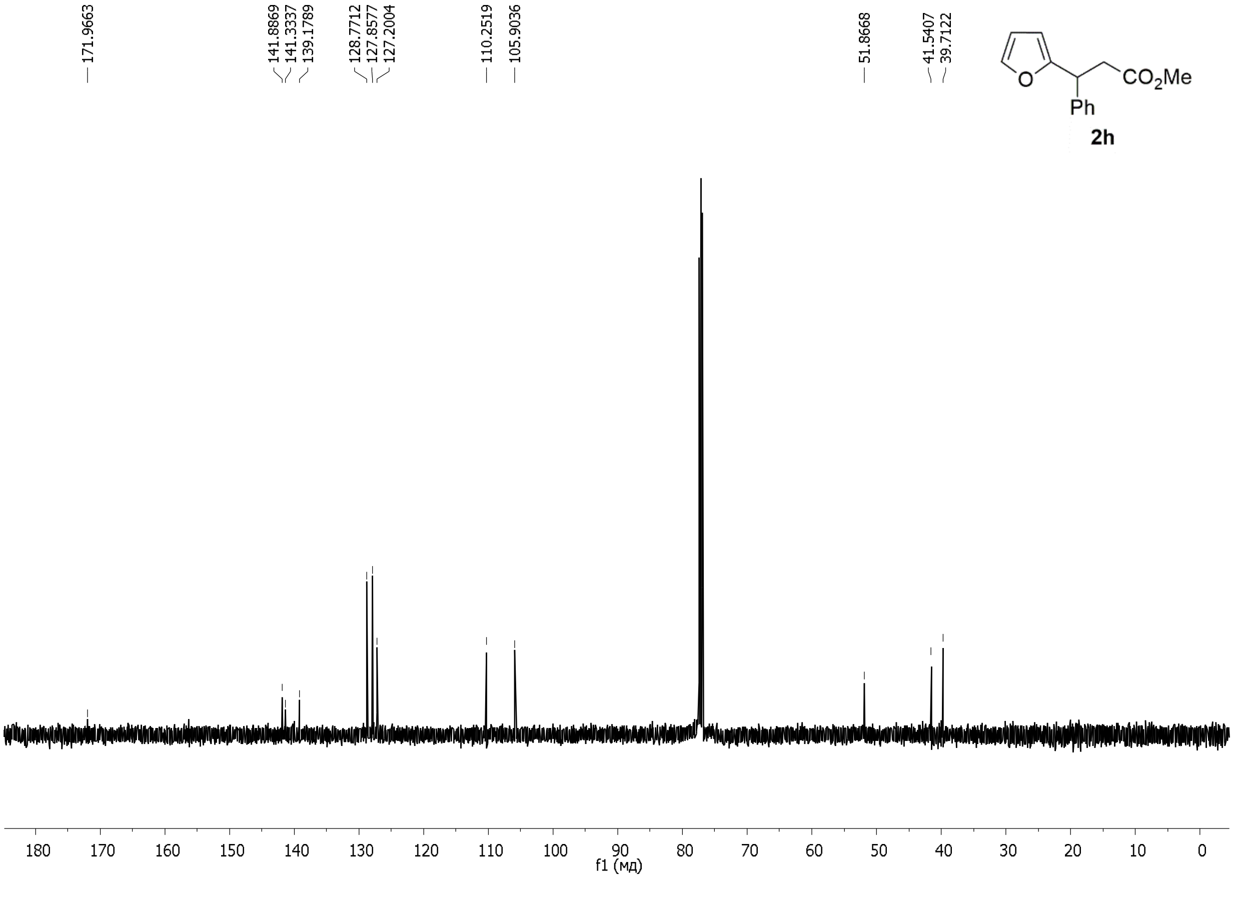


Figure S34. $^{13}$C NMR spectrum of compound **2h** (125MHz, $CDCl_3$).

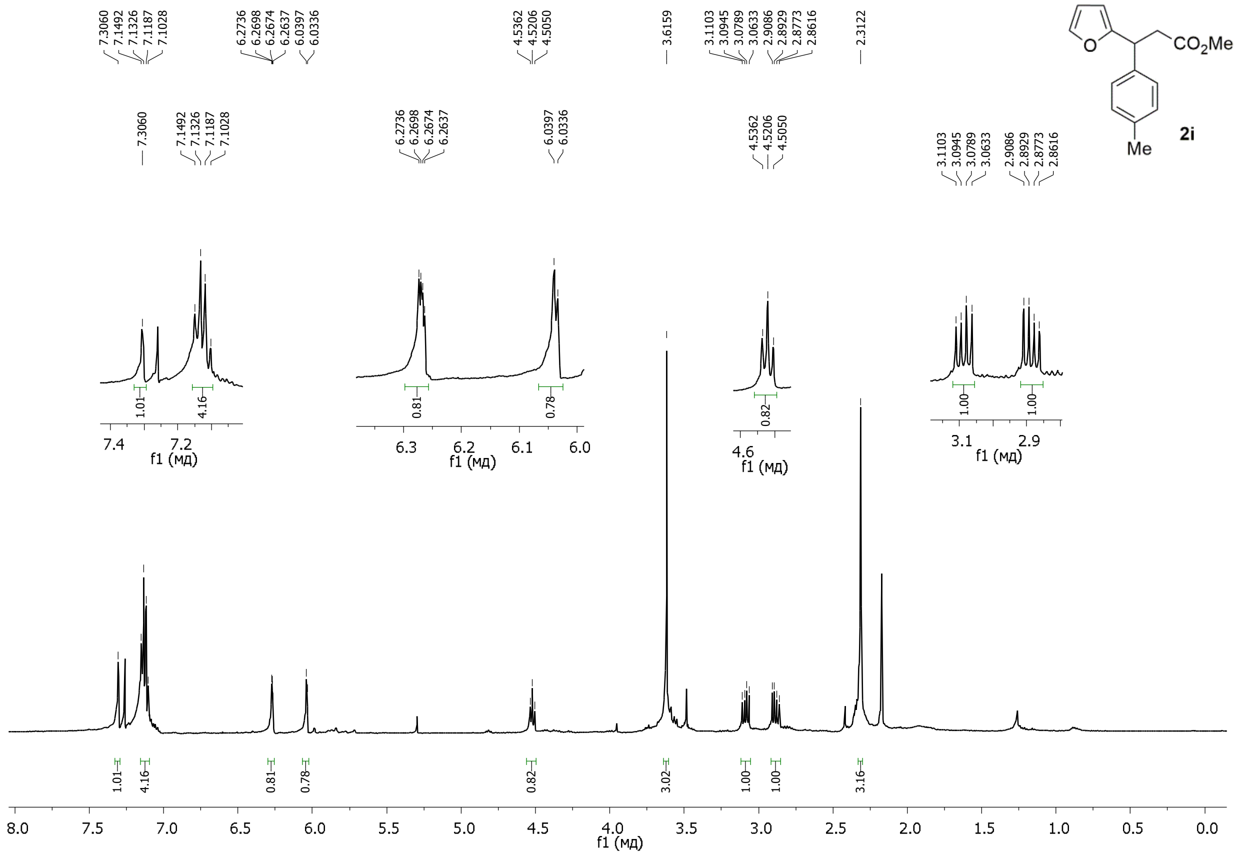

Figure S35. $^{1}H$ NMR spectrum of compound **2i** (500 MHz, $CDCl_3$).

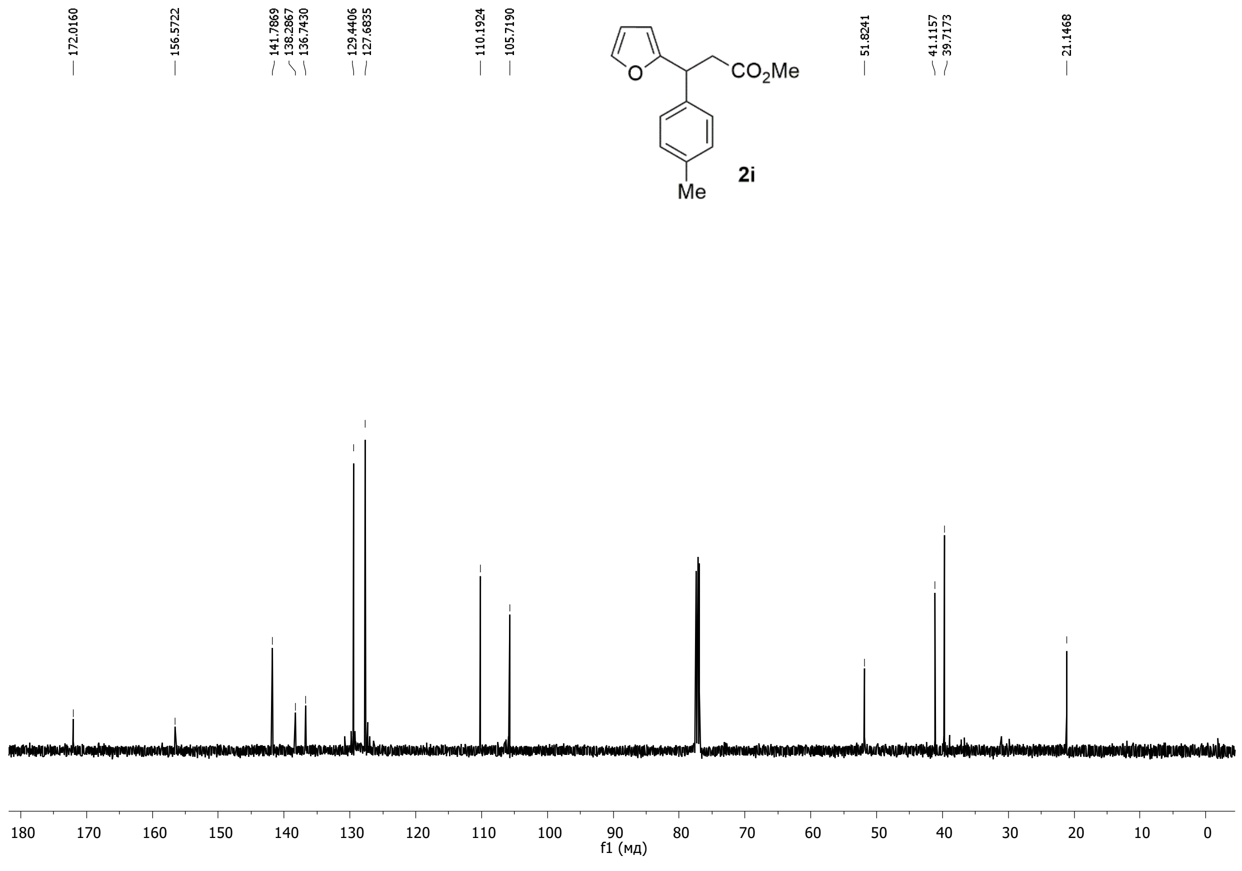

Figure S36. $^{13}C$ NMR spectrum of compound **2i** (125 MHz, $CDCl_3$).

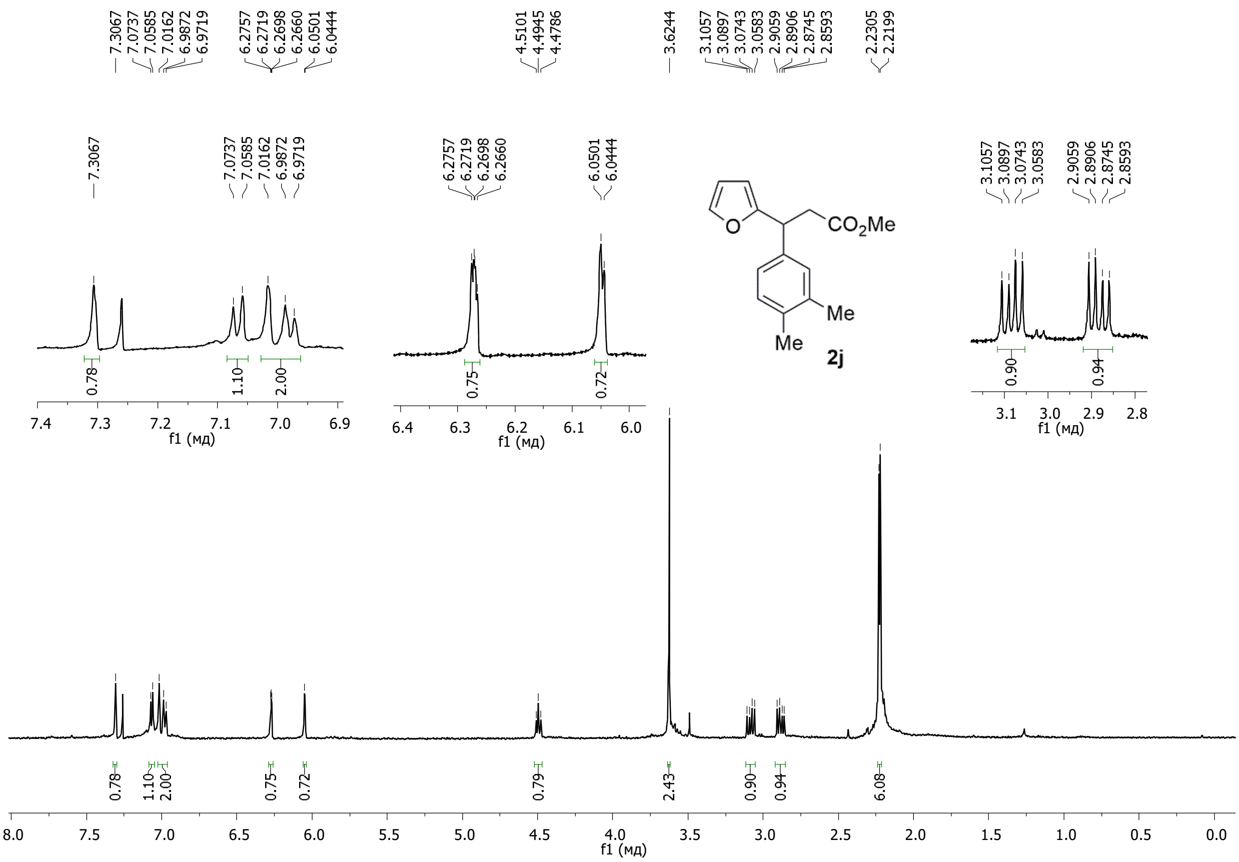

Figure S37. $^{1}$H NMR spectrum of compound **2j** (500 MHz, $CDCl_3$).

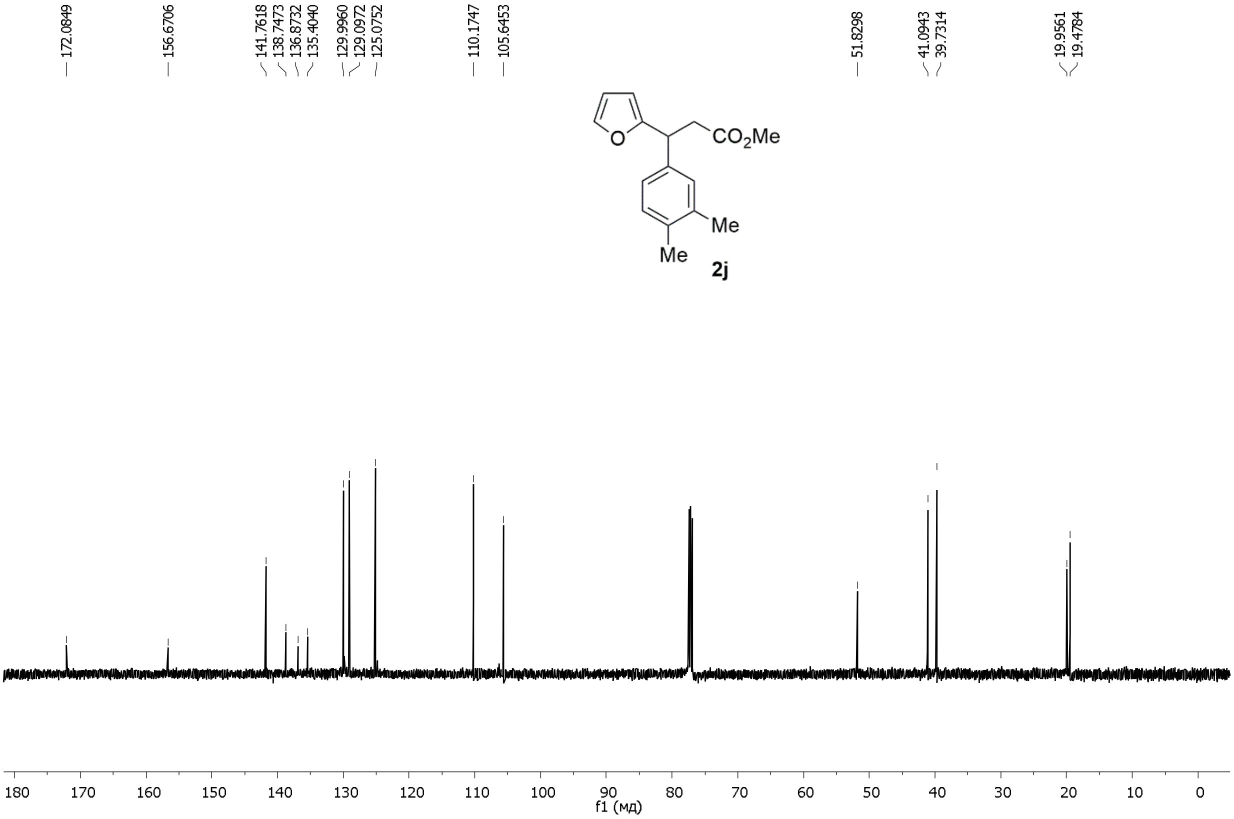

Figure S38. $^{13}$C NMR spectrum of compound **2j** (125 MHz, $CDCl_3$).

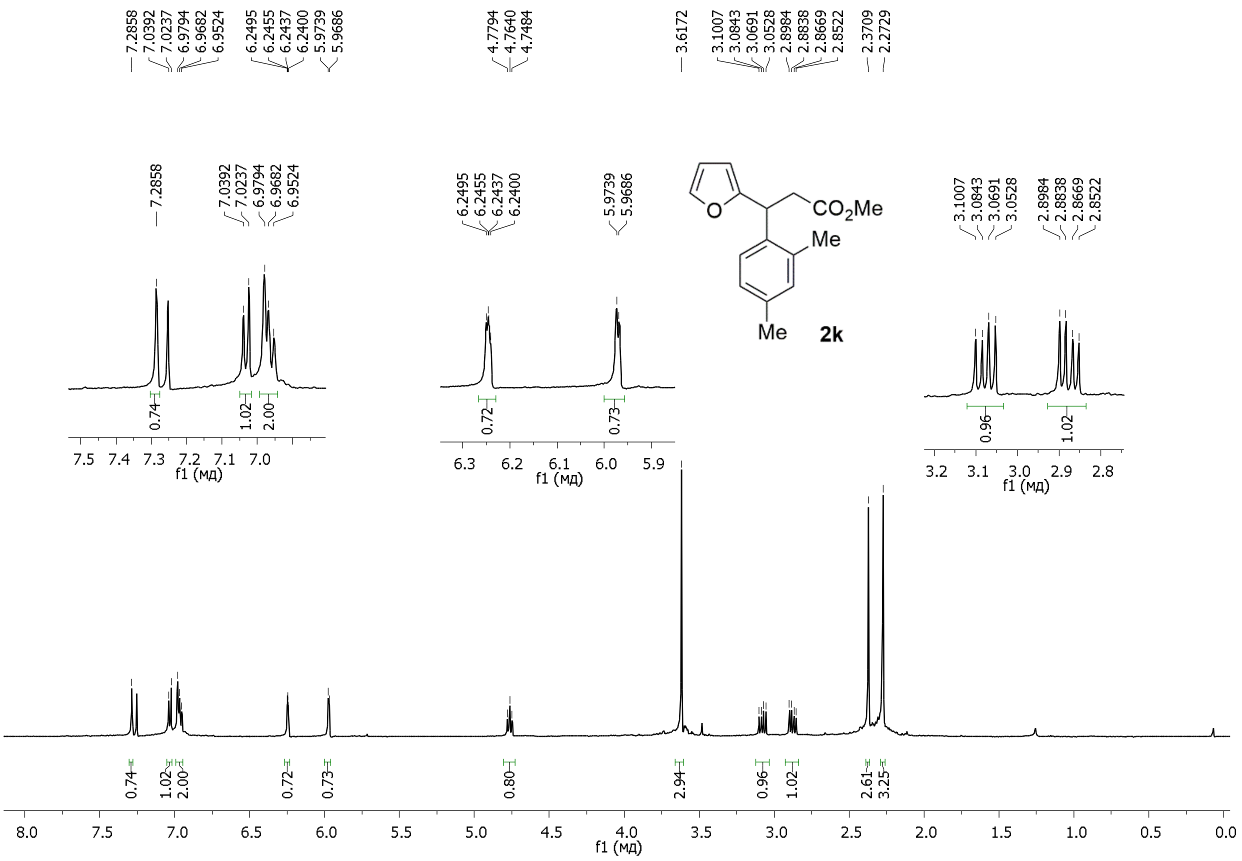


Figure S39. $^1$H NMR spectrum of compound **2k** (500 MHz, $CDCl_3$).

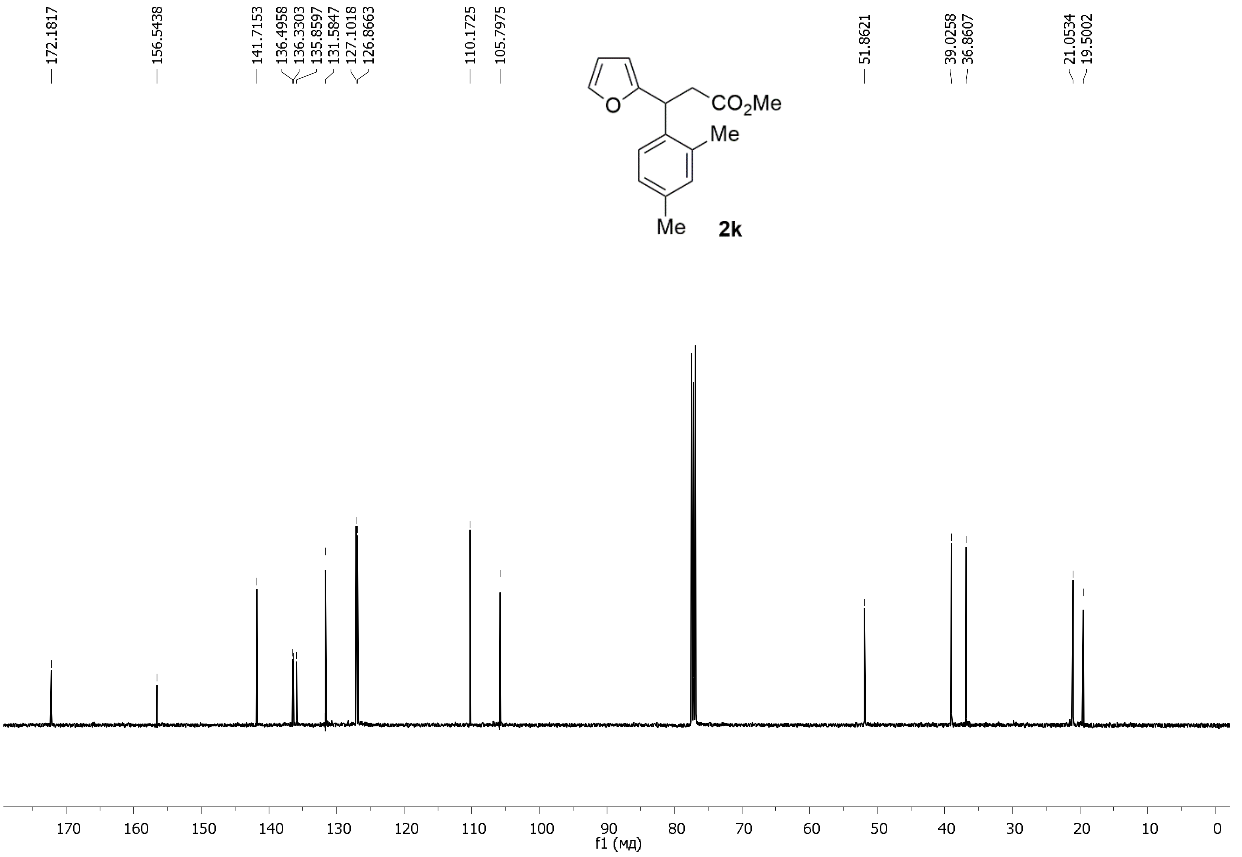


Figure S40. $^{13}$C NMR spectrum of compound **2k** (125 MHz, $CDCl_3$).

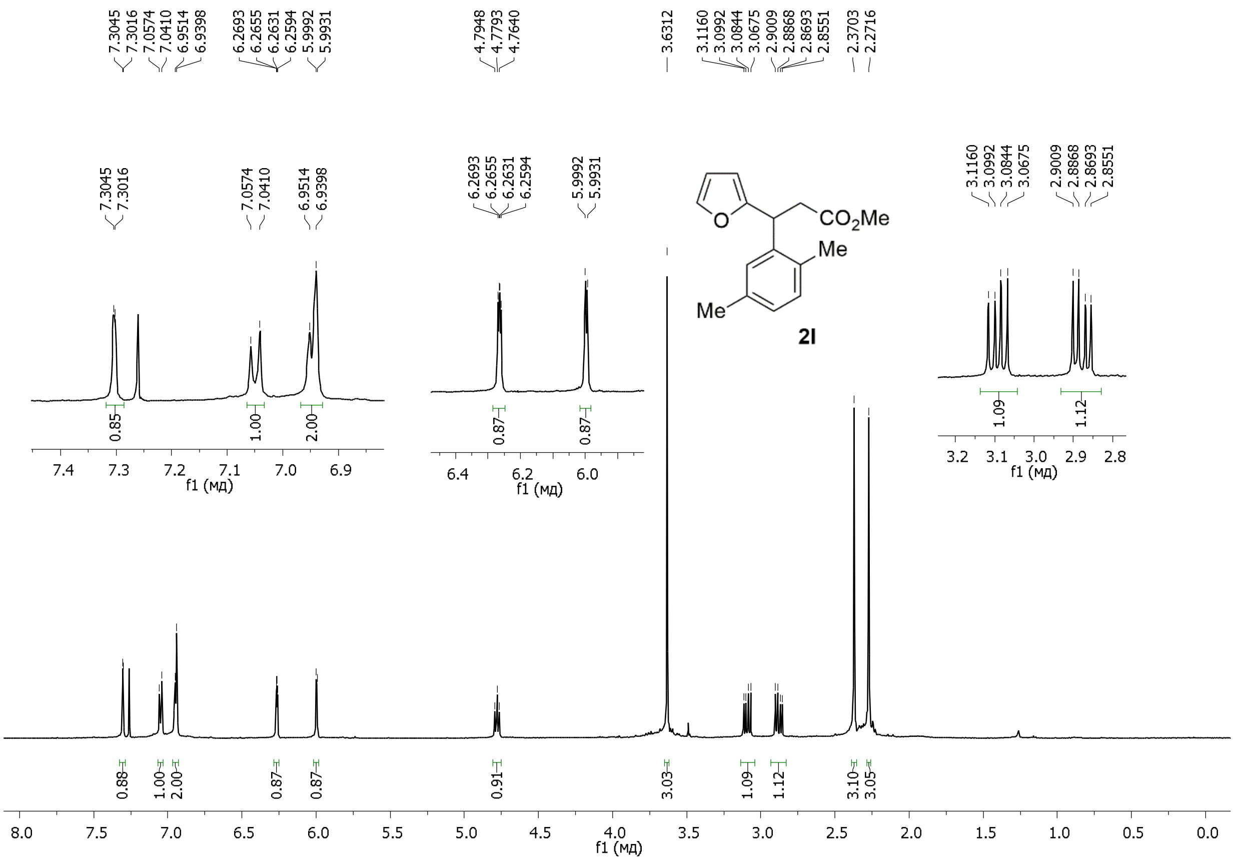


Figure S41. $^1$H NMR spectrum of compound **2l** (500 MHz, $CDCl_3$).

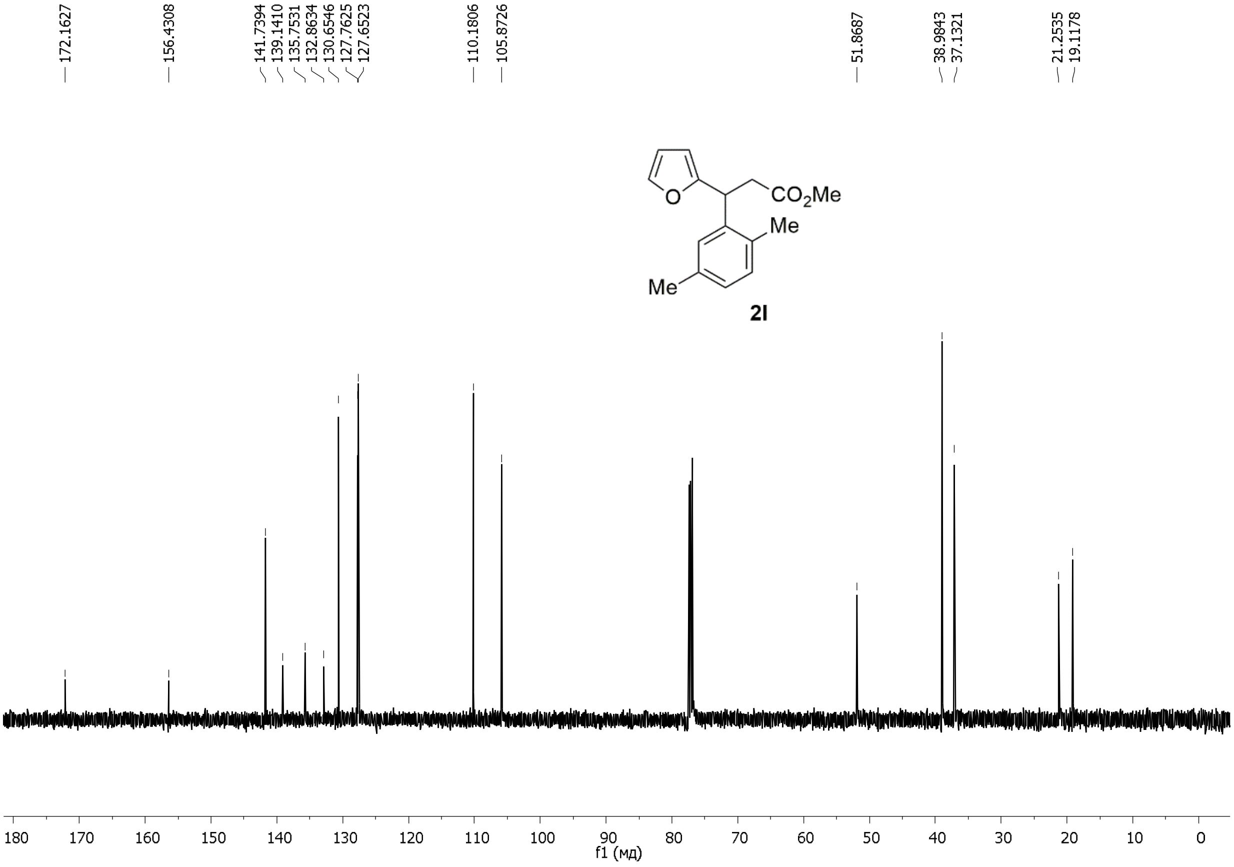


Figure S42. $^{13}$C NMR spectrum of compound **2l** (125 MHz, $CDCl_3$).

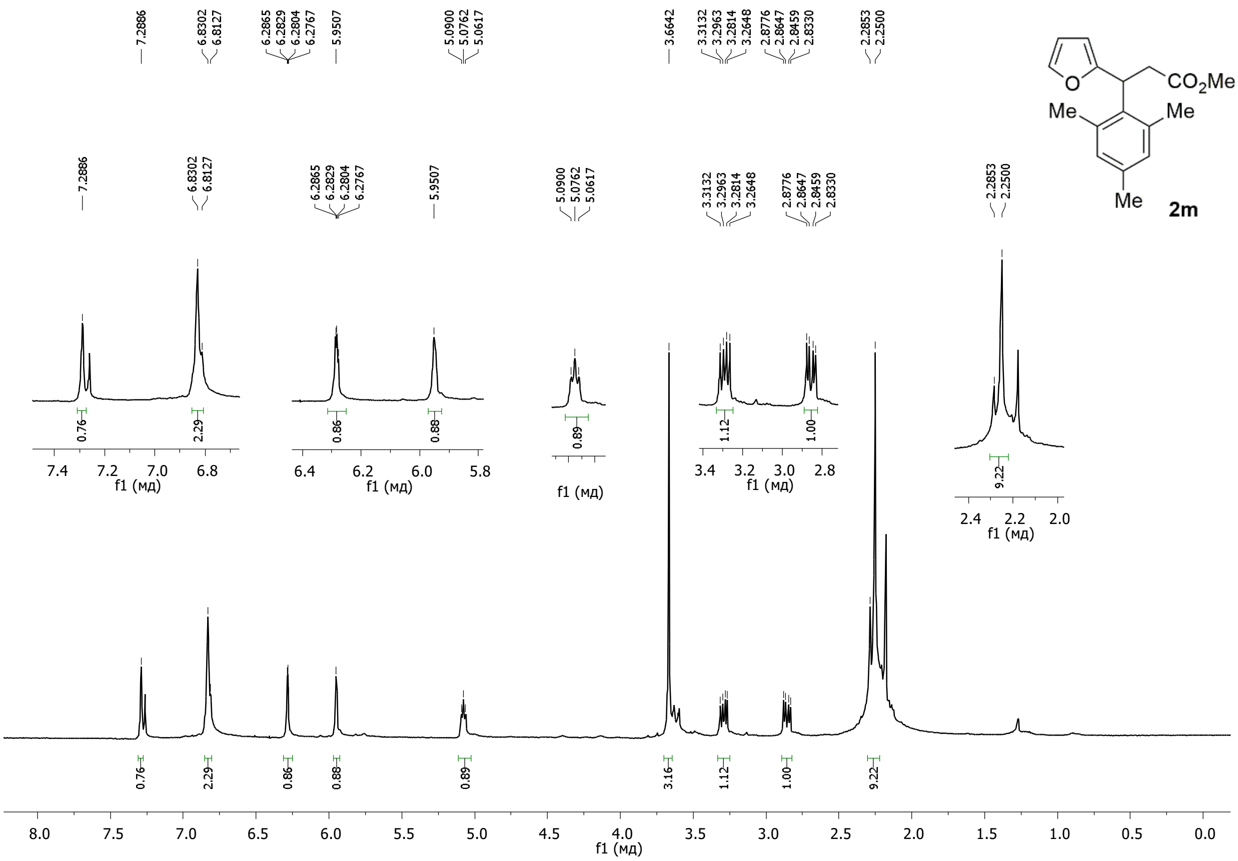

Figure S43. $^{1}$H NMR spectrum of compound **2m** (500 MHz, $CDCl_3$).

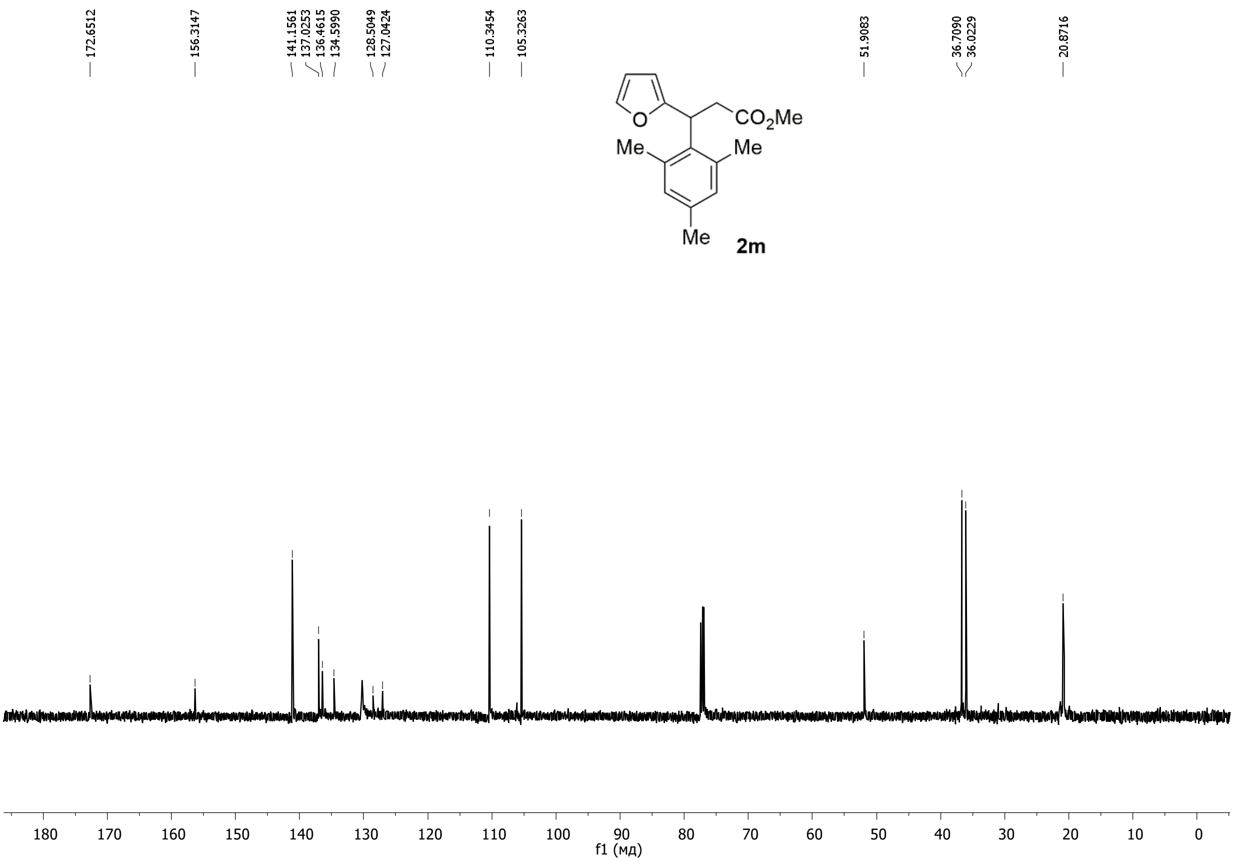

Figure S44. $^{13}$C NMR spectrum of compound **2m** (125 MHz, $CDCl_3$).

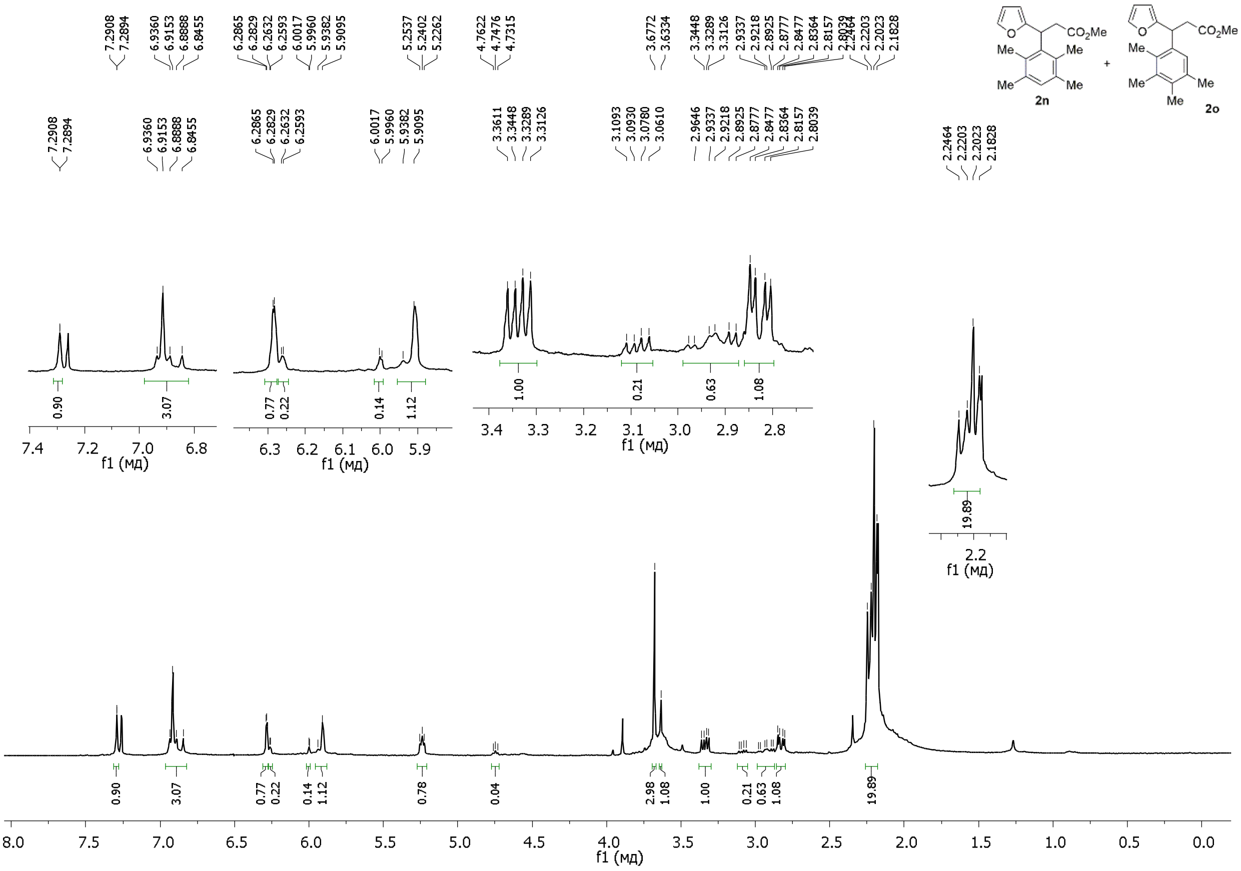


Figure S45. $^{1}$H NMR spectrum of compound **2n** and **2o** (500 MHz, $CDCl_3$).

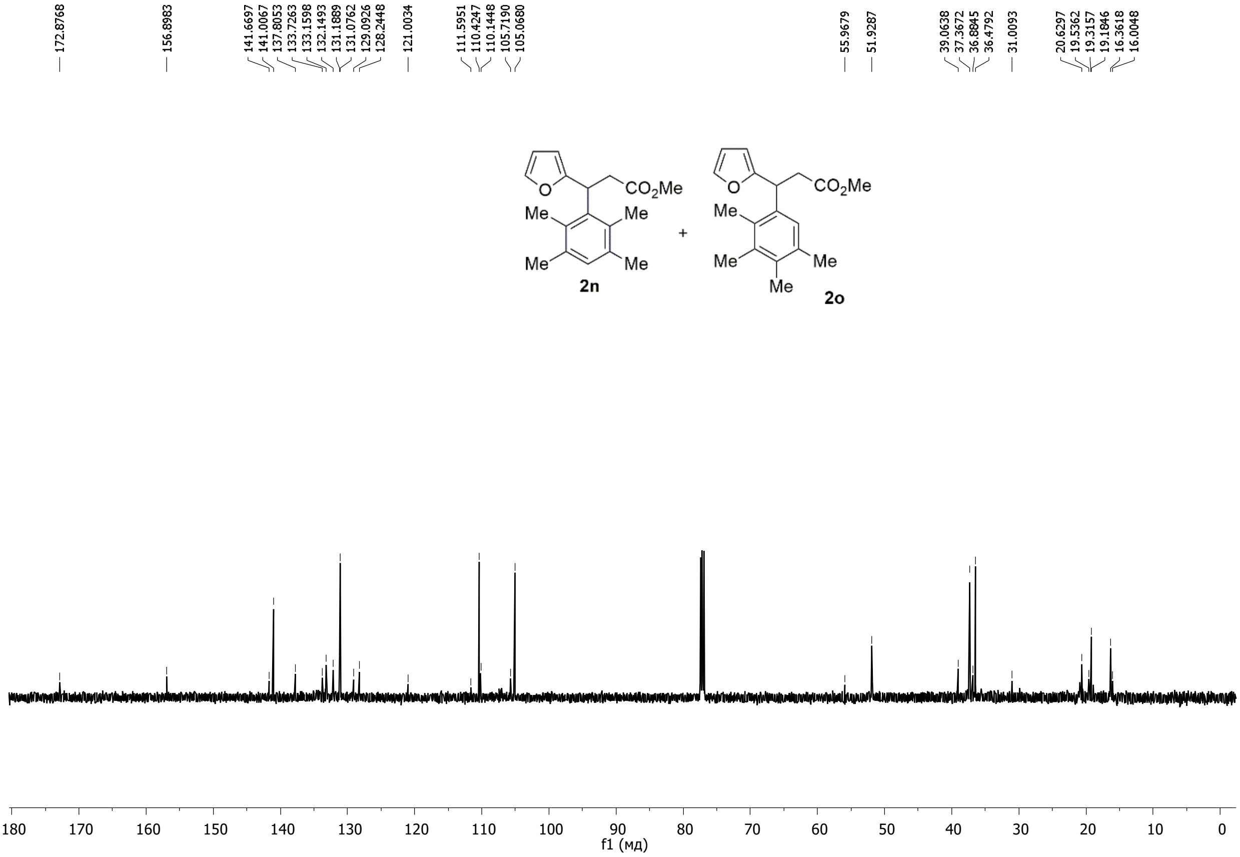


Figure S46. $^{13}$C NMR spectrum of compounds **2n** and **2o** (125 MHz, $CDCl_3$).

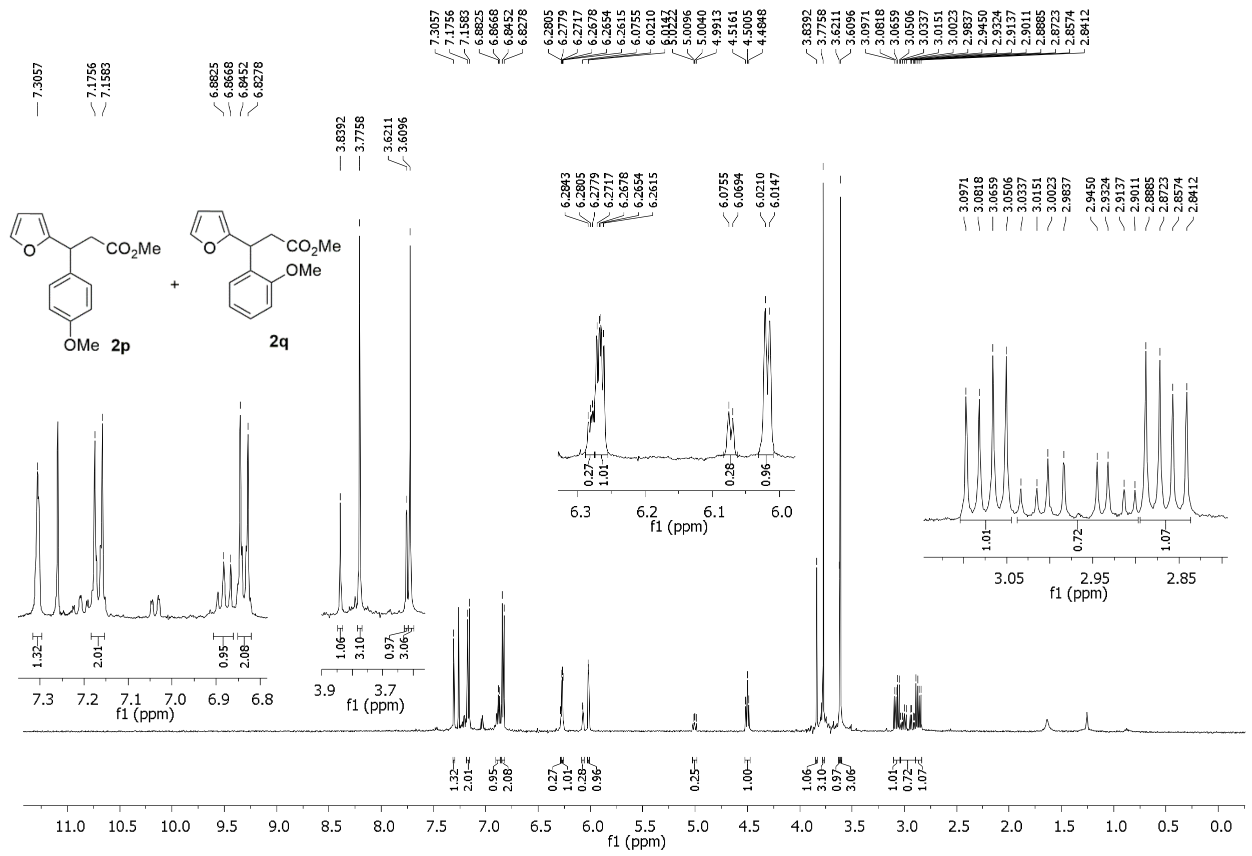

Figure S47. $^{1}$H NMR spectrum of compounds **2p** and **2q** (500 MHz, $CDCl_3$).

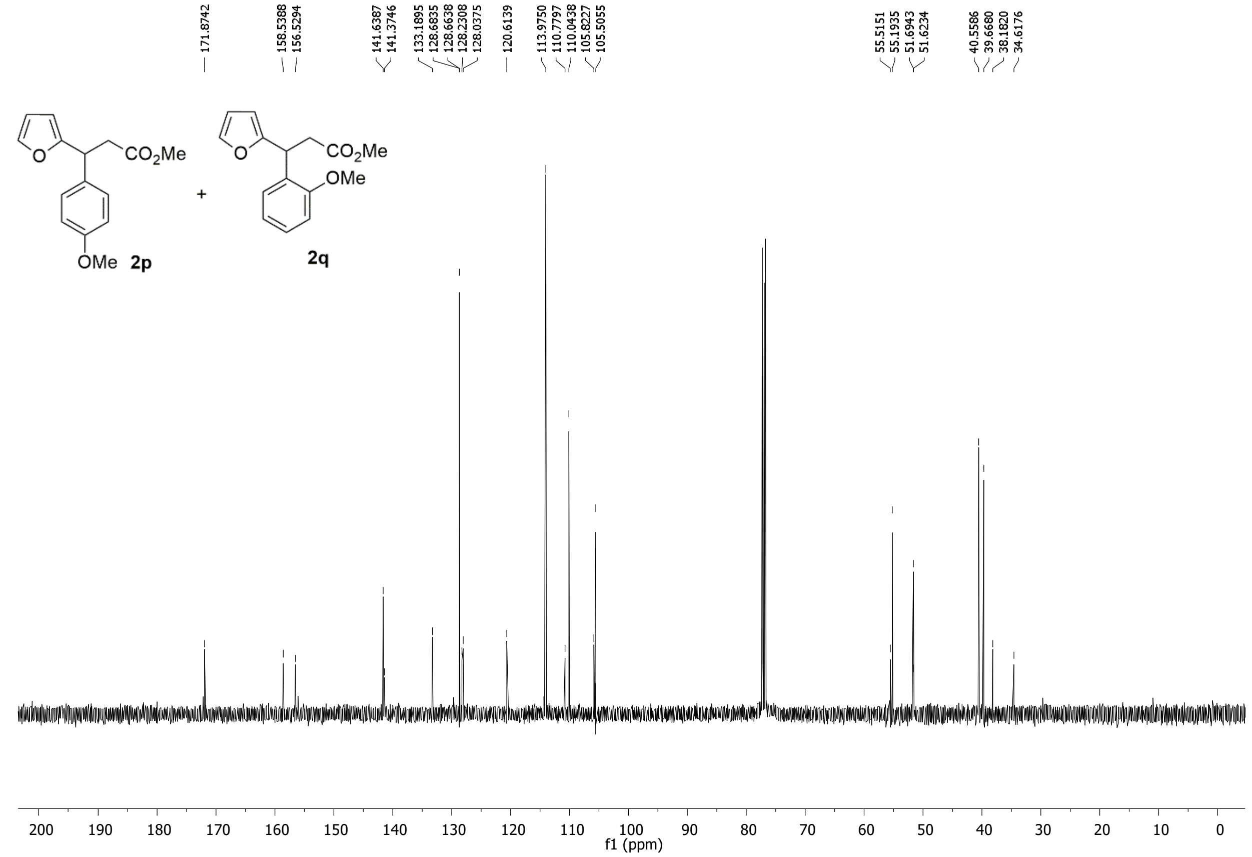

Figure S48. $^{13}$C NMR spectrum of compounds **2p** and **2q** (125 MHz, $CDCl_3$).

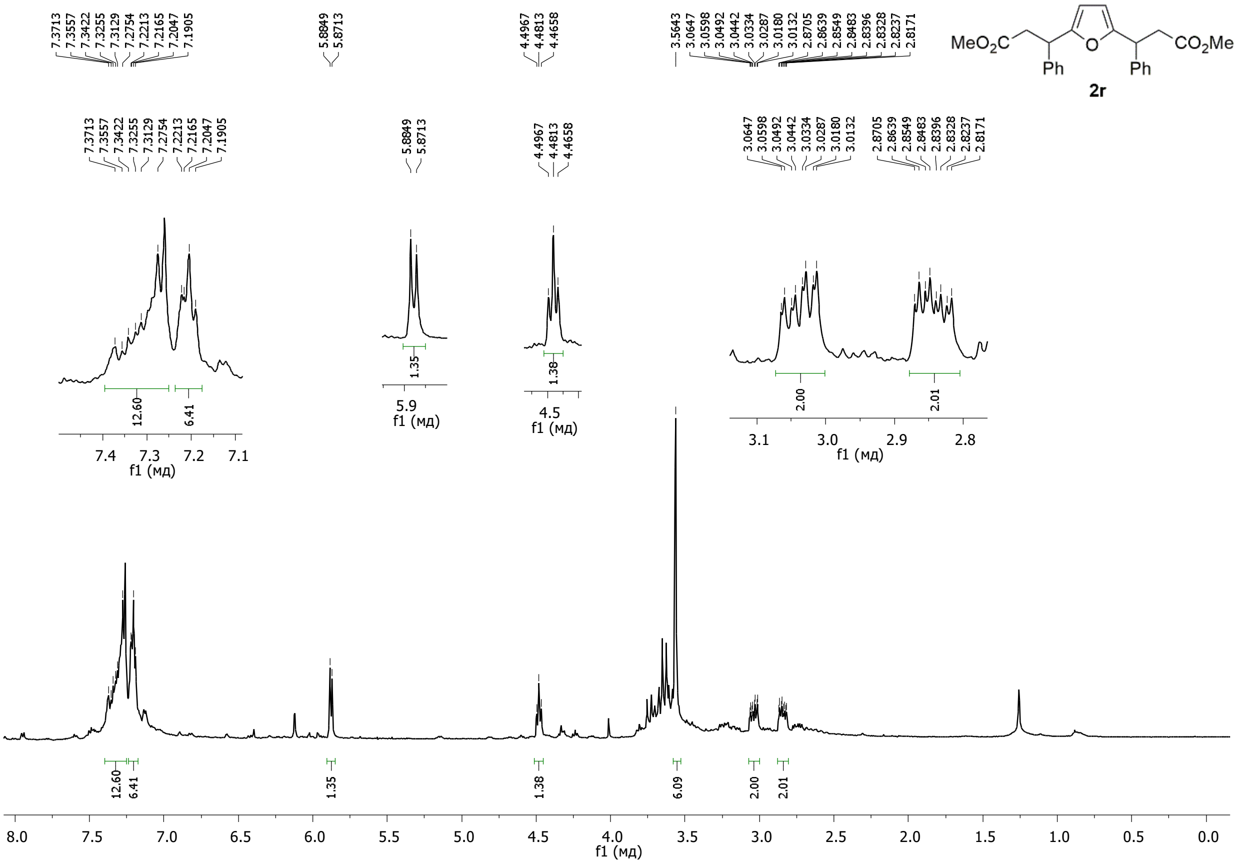


Figure S49. $^{1}$H NMR spectrum of equimolar mixture of diastereomers of compound **2r** (500 MHz, $CDCl_3$).

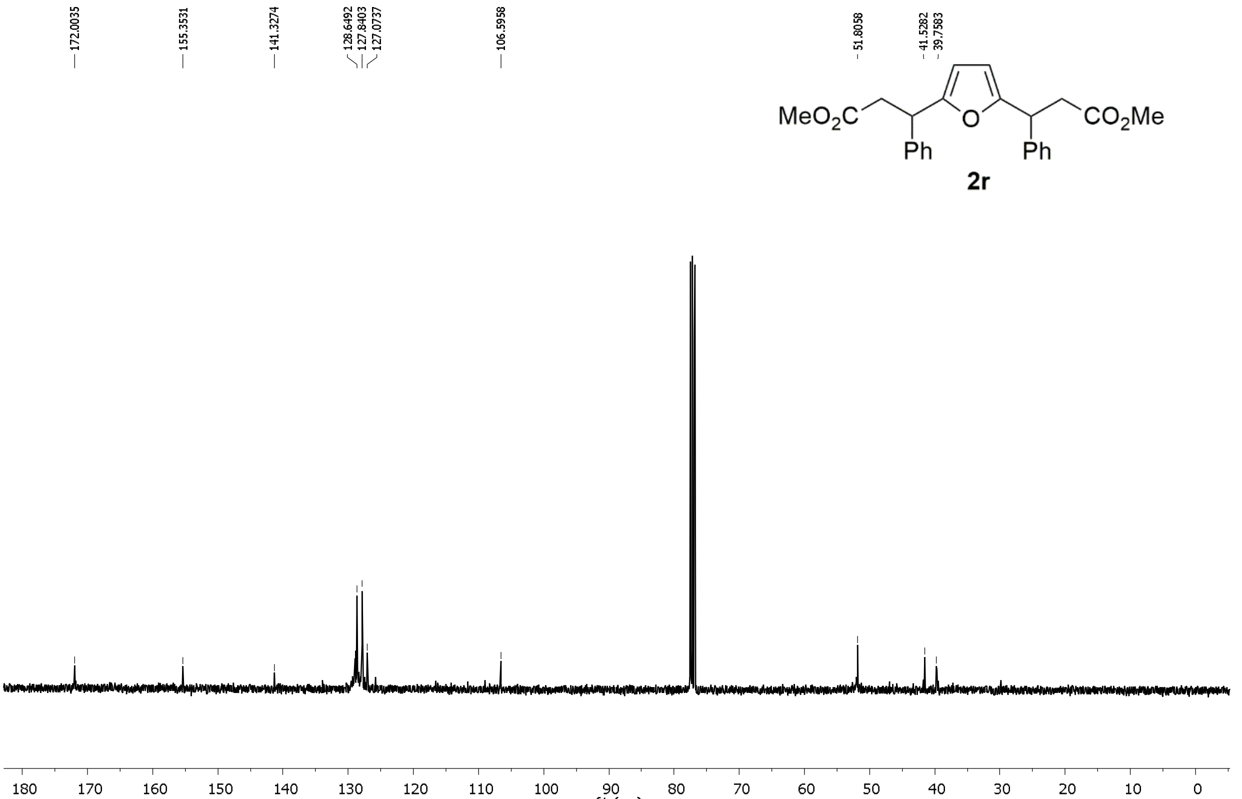


Figure S50. $^{13}$C NMR spectrum of equimolar mixture of diastereomers of compound **2r** (125MHz, $CDCl_3$).

## 2. Results of study of oligomeric compounds by liquid chromatography-high-resolution mass-spectrometry

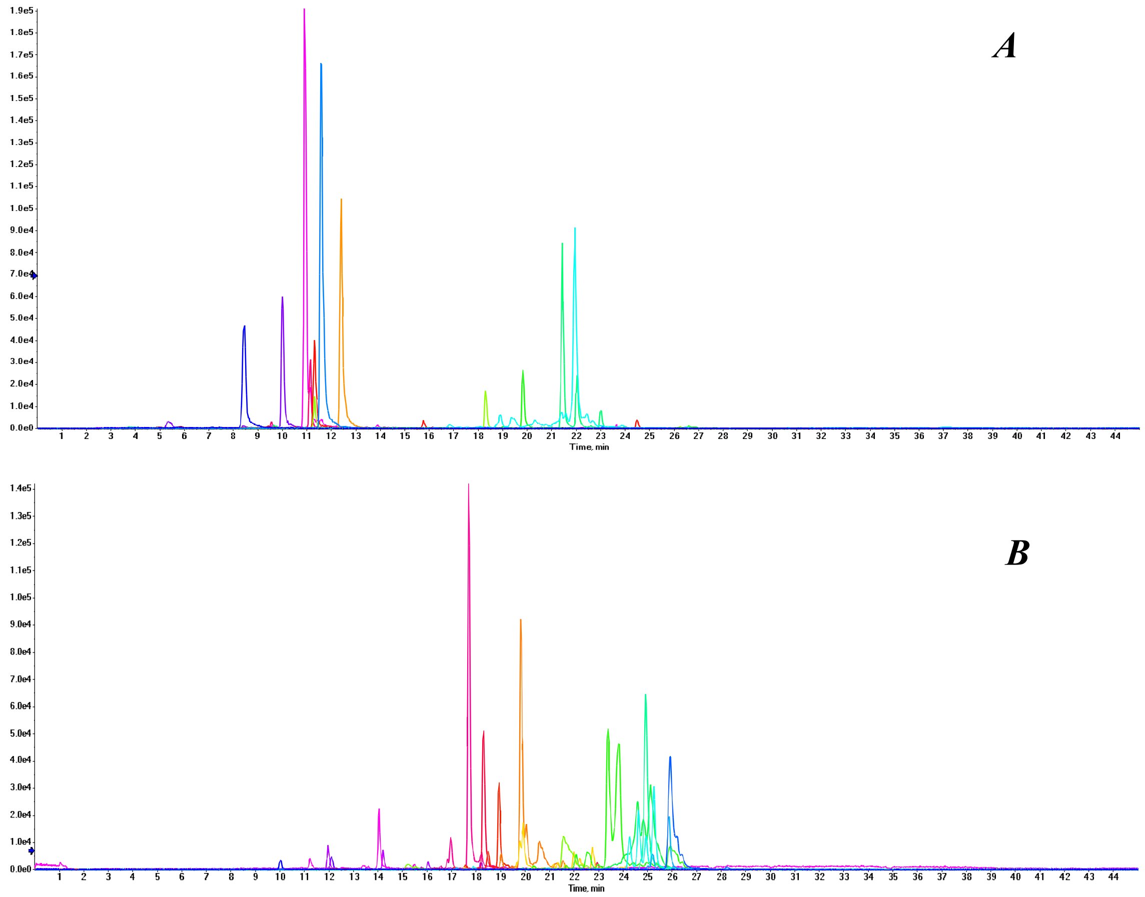


**Figure S51**.Reconstructed extracted ion chromatograms (XIC) of the products of **1a**(A) and **1b**(B) reactions with benzene in $H_2SO_4$

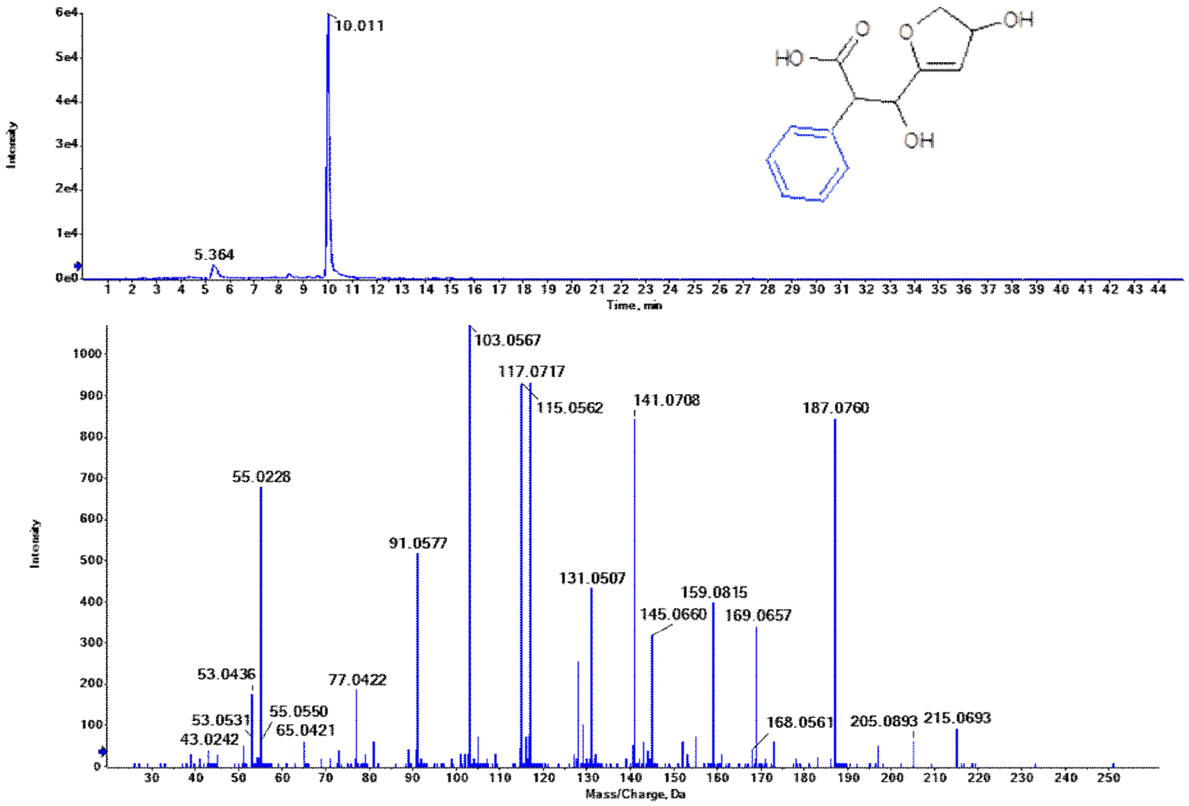


**Figure S52.**Accurate mass XIC chromatogram, tentative structural formula and MS/MS spectrum of the product of **1a** interaction with benzene in sulfuric acid medium with elemental composition $C_{13}H_{14}O_5$

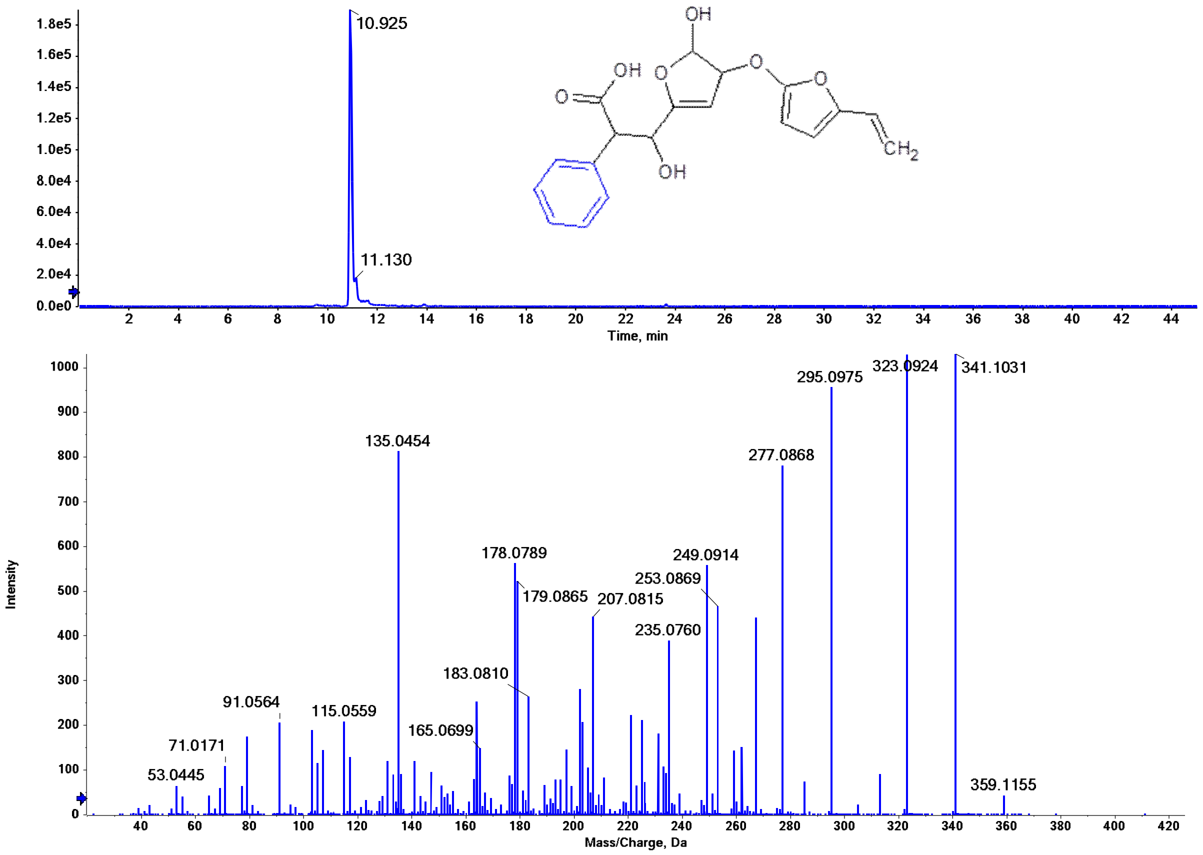


**Figure S53.**Accurate mass XIC chromatogram, tentative structural formula and MS/MS spectrum of the product of **1a** interaction with benzene in sulfuric acid medium with elemental composition $C_{19}H_{18}O_7$

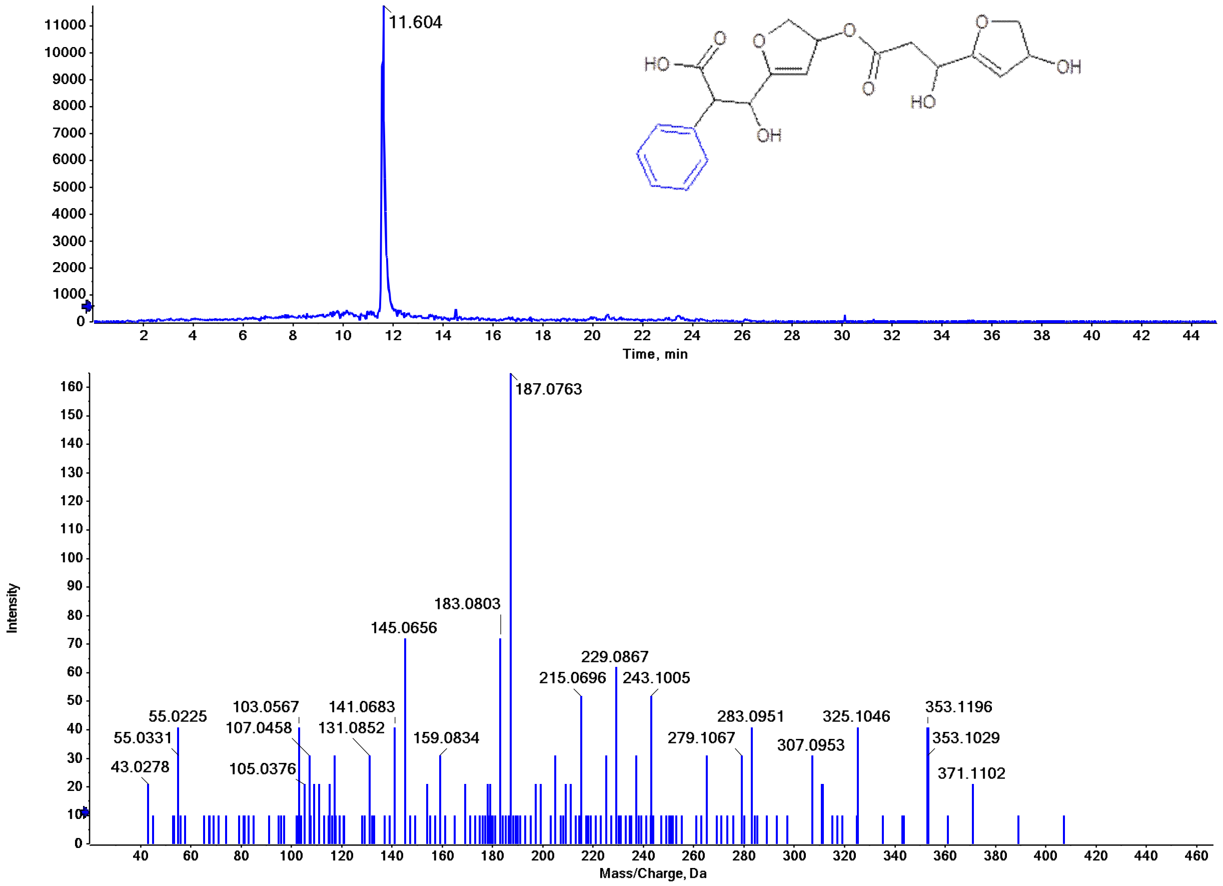


**Figure S54.**Accurate mass XIC chromatogram, tentative structural formula and MS/MS spectrum of the product of **1a** interaction with benzene in sulfuric acid medium with elemental composition $C_{20}H_{22}O_9$

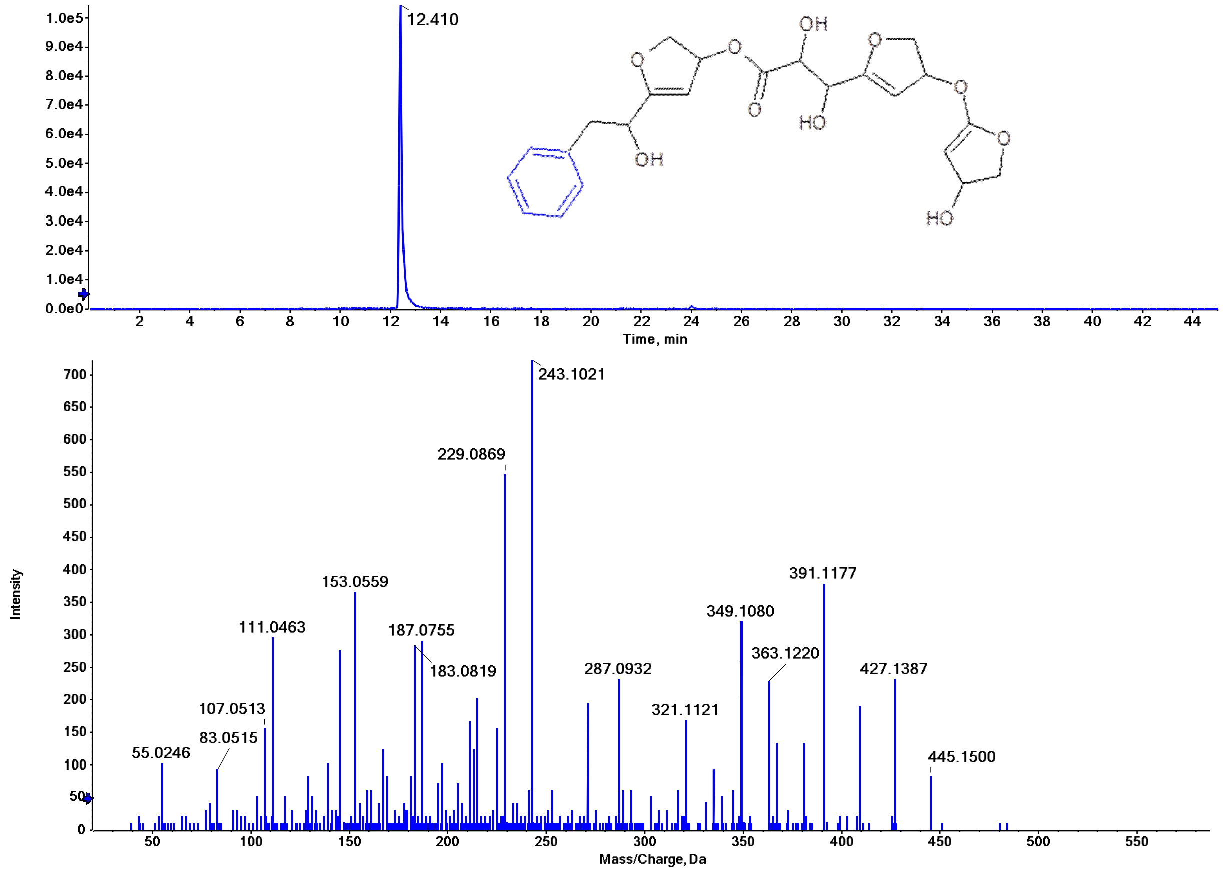


**Figure S55.**Accurate mass XIC chromatogram, tentative structural formula and MS/MS spectrum of the product of **1a** interaction with benzene in sulfuric acid medium with elemental composition $C_{23}H_{26}O_{10}$

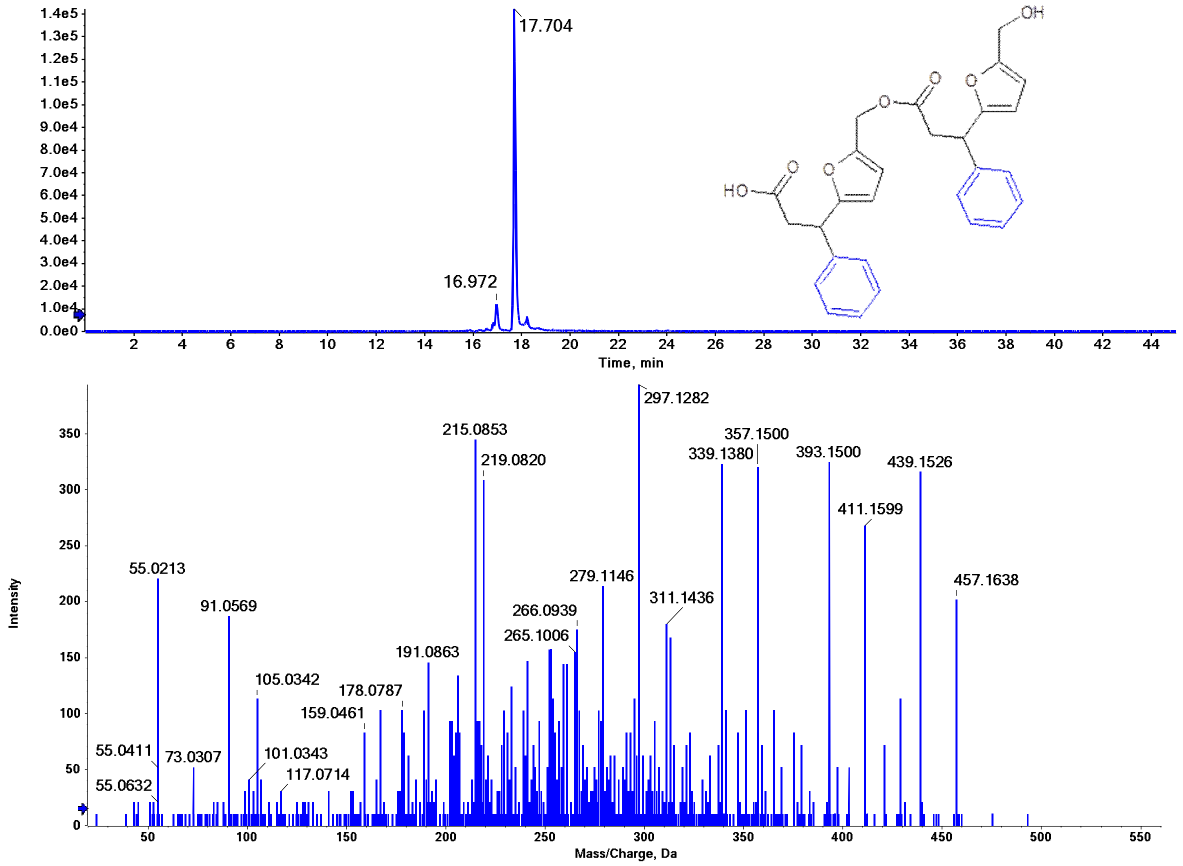


**Figure S56.** Accurate mass XIC chromatogram, tentative structural formula and MS/MS spectrum of the product of **1b** interaction with benzene in sulfuric acid medium with elemental composition $C_{28}H_{26}O_7$

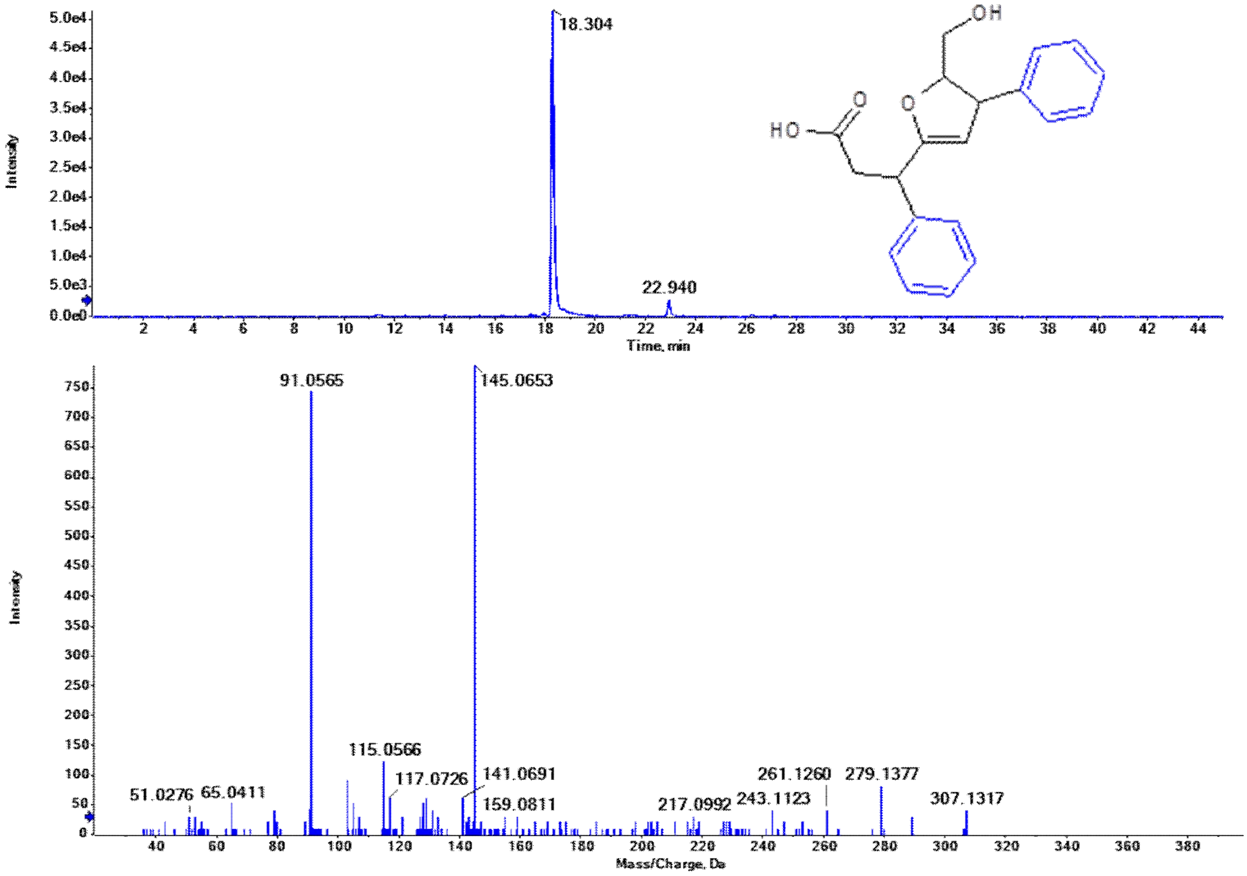


**Figure S57.** Accurate mass XIC chromatogram, tentative structural formula and MS/MS spectrum of the product of **1b** interaction with benzene in sulfuric acid medium with elemental composition $C_{20}H_{20}O_4$

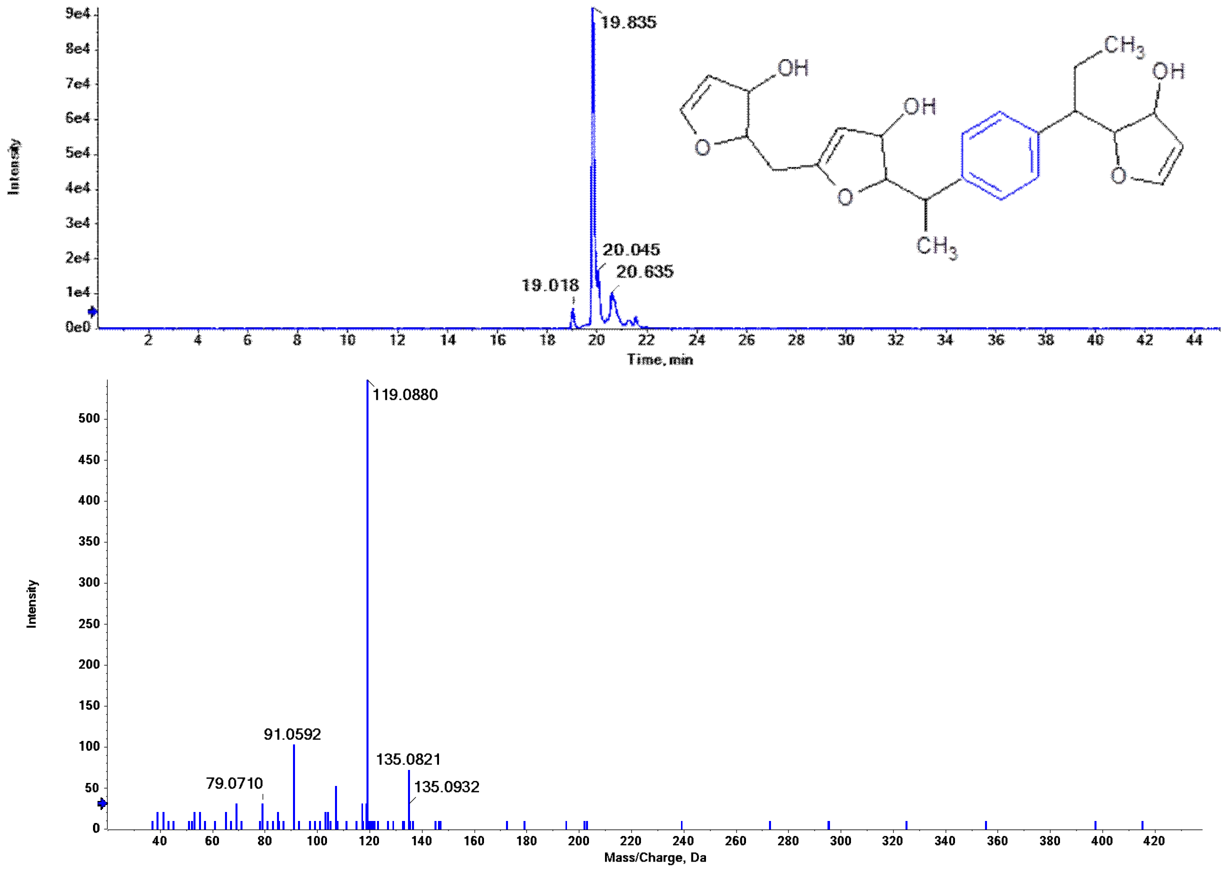


**Figure S58.**Accurate mass XIC chromatogram, tentative structural formula and MS/MS spectrum of the product of **1b** interaction with benzene in sulfuric acid medium with elemental composition $C_{24}H_{30}O_6$

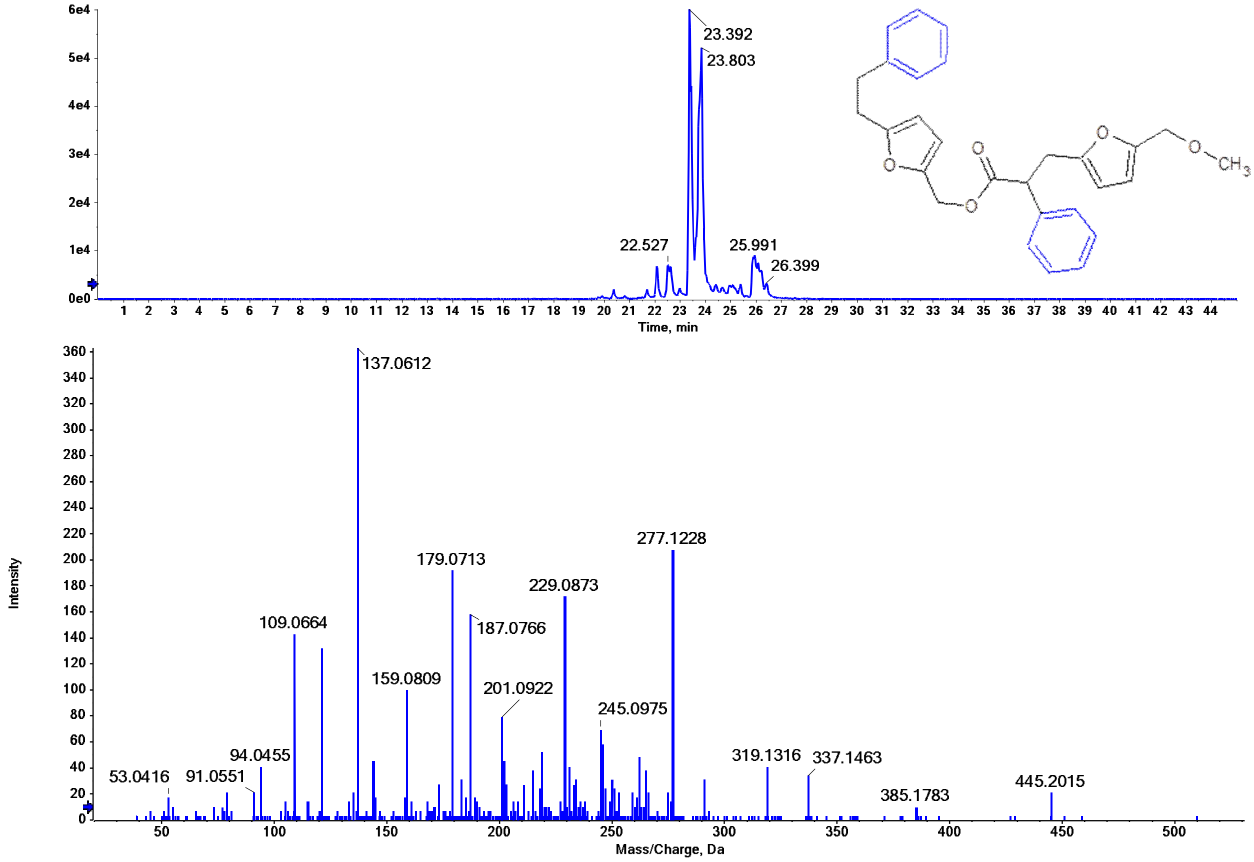


**Figure S59.**Accurate mass XIC chromatogram, tentative structural formula and MS/MS spectrum of the product of **1b** interaction with benzene in sulfuric acid medium with elemental composition $C_{28}H_{28}O_5$

## 3. Study of biological activity of compounds 1 and 2

MICs of furan compounds against *Escherichia coli* ATCC 25922, *Staphylococcus aureus* ATCC 29213, *Candida albicans* ATCC 10231 were determined using broth microdilution as described in ISO 20776-1:2019 and ISO 16256:2021. Stock solutions of furan compounds in neat (pure) DMSO were prepared in sterile tubes and used on the same day. Two-fold dilutions of the furan compounds in the appropriate culture medium were added to the wells of a 96-well plate. The final concentrations of the test substances (after inoculation) were 256, 128, 64, 32, 16, 8, 4, 2, 1 µg/ml. Solutions of furan compounds were added to the wells of the plates, 50 µl per well for *S. aureus* and *E. coli*, 100 µl for *C. albicans*.

RPMI-1640 medium, buffered with MOPS [3-(N-morpholino)propanesulfonic acid] containing l-glutamine and lacking sodium bicarbonate was used for *C. albicans*. The medium for *E. coli* and *S. aureus* was Mueller-Hinton broth. The fungus inoculum was prepared in the test medium and adjusted to match the turbidity of a 0.5 McFarland standard. A 1:100 dilution followed by a 1:20 dilution was performed for the yeast strain to obtain a final inoculum ranging from 0.5 to 2.5 x $10^3$ CFU/ml. Then, 100 µl of fungal inoculum was added to each well containing furan compounds.

Bacterial inoculums were prepared in sterile sodium chloride solution and adjusted to 0.5 McFarland standard. A volume of 50 µl of this suspension was diluted in 10 ml of Mueller-Hinton broth until a concentration of approximately $5 \times 10^5$ CFU/ml was reached. Of this suspension, 50 µl was inoculated into each furan compounds-containing wells.

To ensure that the inoculum contained the required number of cells, the viability of the inoculum suspensions was counted. 100 µl of the inoculum was taken from the growth control tube immediately after inoculation and diluted in 9.9 ml of sodium chloride solution. 100 µl of this dilution was applied to the surface of a suitable agar plate (Sabouraud dextrose agar plate for *C. albicans*, Trypticase soy agar plate for *S. aureus*, *E. coli*) which were then incubated overnight.

After inoculation, the plates were incubated at 37 °C for 18 h for bacterial strains, 22 h for *C. albicans*. The susceptibility to furans was assessed on the basis of visual observation of growth the strains in the culture media. The minimal inhibitory concentration (MIC) is the lowest concentration of an antimicrobial that inhibits visible growth of a bacterial culture under a defined set of experimental conditions.

After incubation in the thermostat of agar plates with dose control inoculum, the number of cells contained in the wells of the plates was counted, taking into account the dilution with the test objects.

The number of cells, contained in the wells, was counted after incubation (with control of inoculum dose) taking into account the dilution with test objects. The number of culturable bacteria present per 1 ml of culture (CFU/ml) was 5.1×$10^5$ for *E. coli*, 4.9×$10^5$ for *S. aureus*, and 1.0×$10^3$ for *C.albicans*. Thus, control of the inoculum dose confirmed the required number of cells inoculated into the wells of the bacterial plate, ranging from 2x$10^5$ CFU/ml to 8x$10^5$ CFU/ml. For yeast-like microorganisms ranging from 0.5×$10^3$ to 2.5×$10^3$ CFU/ml.

The results were read in the presence of sufficient growth of the test organism (i.e. turbidity in the positive growth control) and the absence of growth in the uninoculated control. The amount of growth in each well was compared with the amount of growth in the positive growth control and the MIC was recorded as the lowest concentration of agent that completely inhibited growth according to Figure S60.

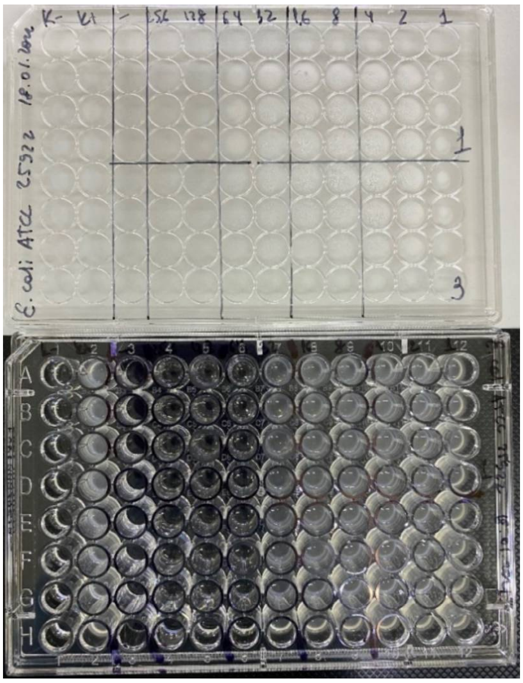

**Figure S60.** Plate inoculated with *E. coli* in the presence of test objects №1 and №3.

The results of the evaluation of the antimicrobial activity of the test objects are shown in Table S1.

**Table S1.** Evaluation of the antimicrobial activity of the test objects

| Compounds | MIC, μg/ml | | |
|---|---|---|---|
| | *E. coli* | *S. aureus* | *C. albicans* |
| **2d** | 64 | 64 | 64 |
| **1a** | 64 | 128 | 64 |
| **3h** | 64 | 128 | 64 |
| **1g** | 64 | 128 | 64 |
| **1b** | 64 | 128 | 64 |
| **2e** | 128 | 128 | 64 |
| **2a** | 128 | 128 | 64 |
| **2k** | 128 | 128 | 64 |

| | | | |
|---|---|---|---|
| **2l** | 64 | 128 | 64 |
| **2p+2q** | 64 | 128 | 64 |
| **2j** | 128 | 128 | 64 |
| **2g** | 64 | 128 | 64 |
| **1e** | 64 | 128 | 64 |
| **1h** | 64 | 128 | 64 |
| **1d** | 128 | 128 | 64 |
| **1c** | 64 | 128 | 64 |
| **1f** | 64 | 128 | 64 |
| **1i** | 64 | 128 | 64 |
| **2i** | 64 | >256 | 64 |
| **2m** | 64 | >256 | 64 |
| **2n+2o** | 128 | 128 | 64 |
| **2b** | 128 | 128 | 64 |
| **2f** | 64 | 128 | 64 |
| **2r** | >256 | >256 | 64 |

## 4. $^{1}$H and $^{13}$C NMR spectra of cations Ae and Ah in TfOH

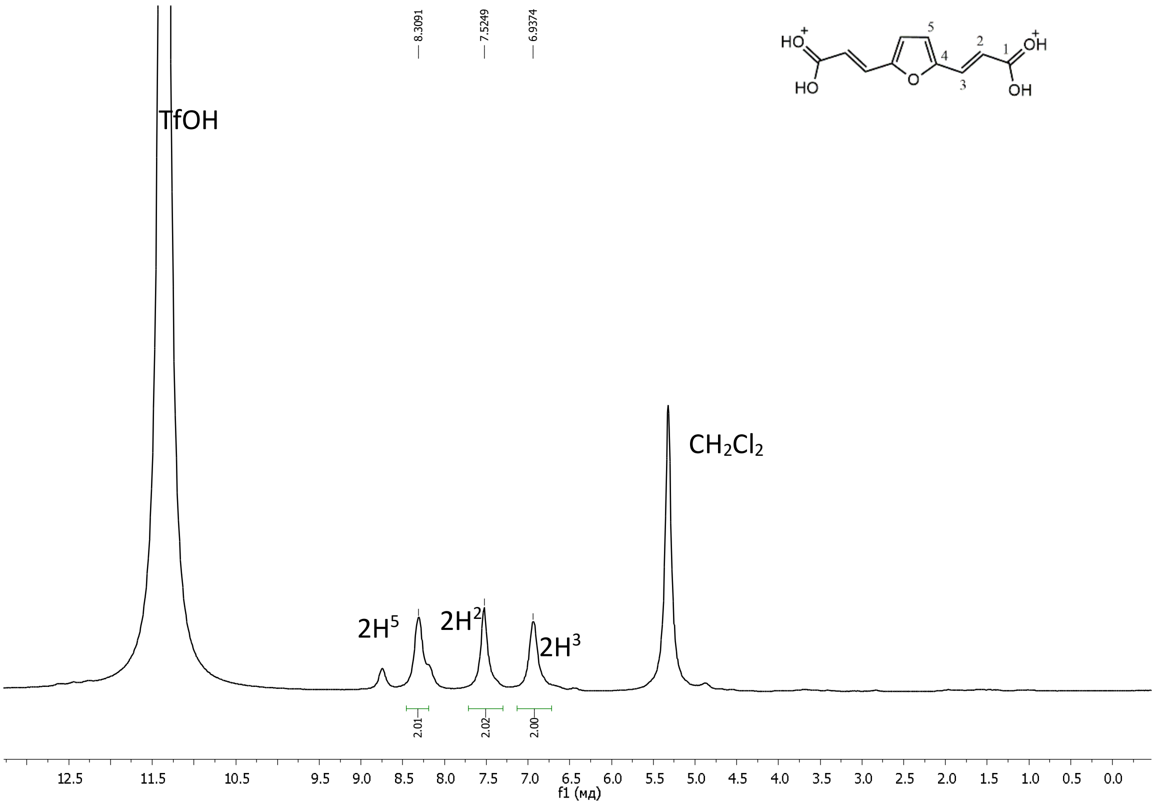


Figure S61. $^{1}$H NMR spectrum of cation **Aa** in TFOH at room temperature ($CH_2Cl_2$ as internal standard, 400 MHz).

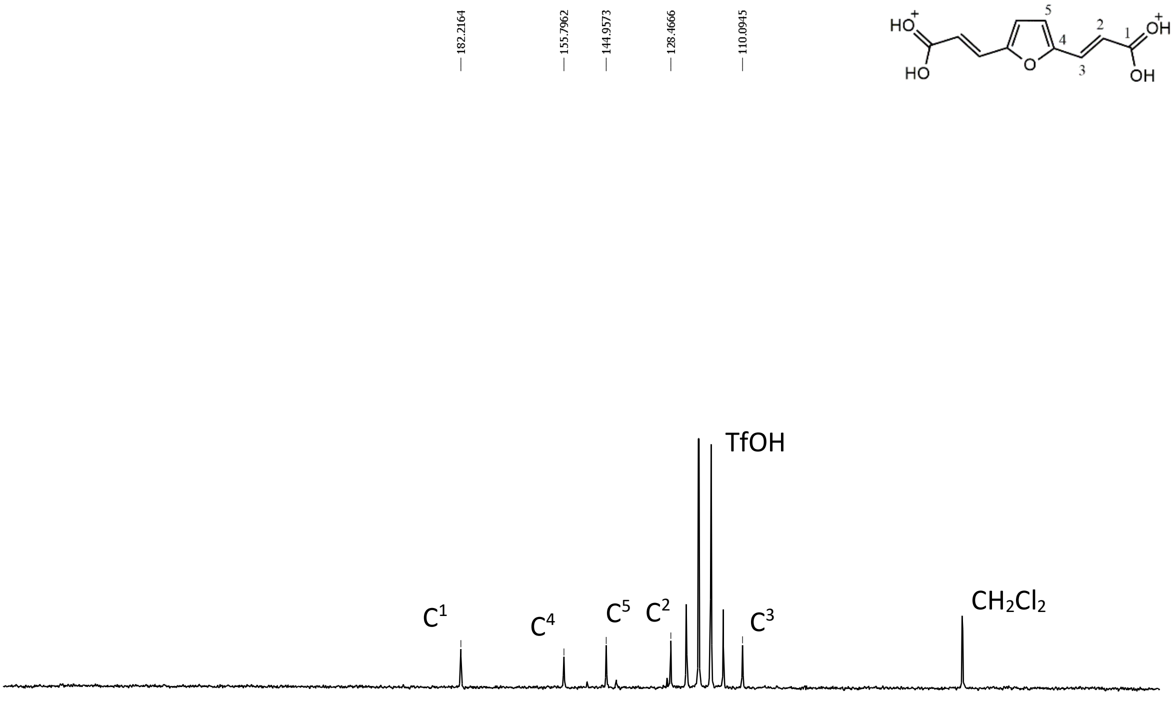


Figure S62. $^{13}$C NMR spectrum of cation **Aa** in TfOH at room temperature ($CH_2Cl_2$ as internal standard, 100 MHz).

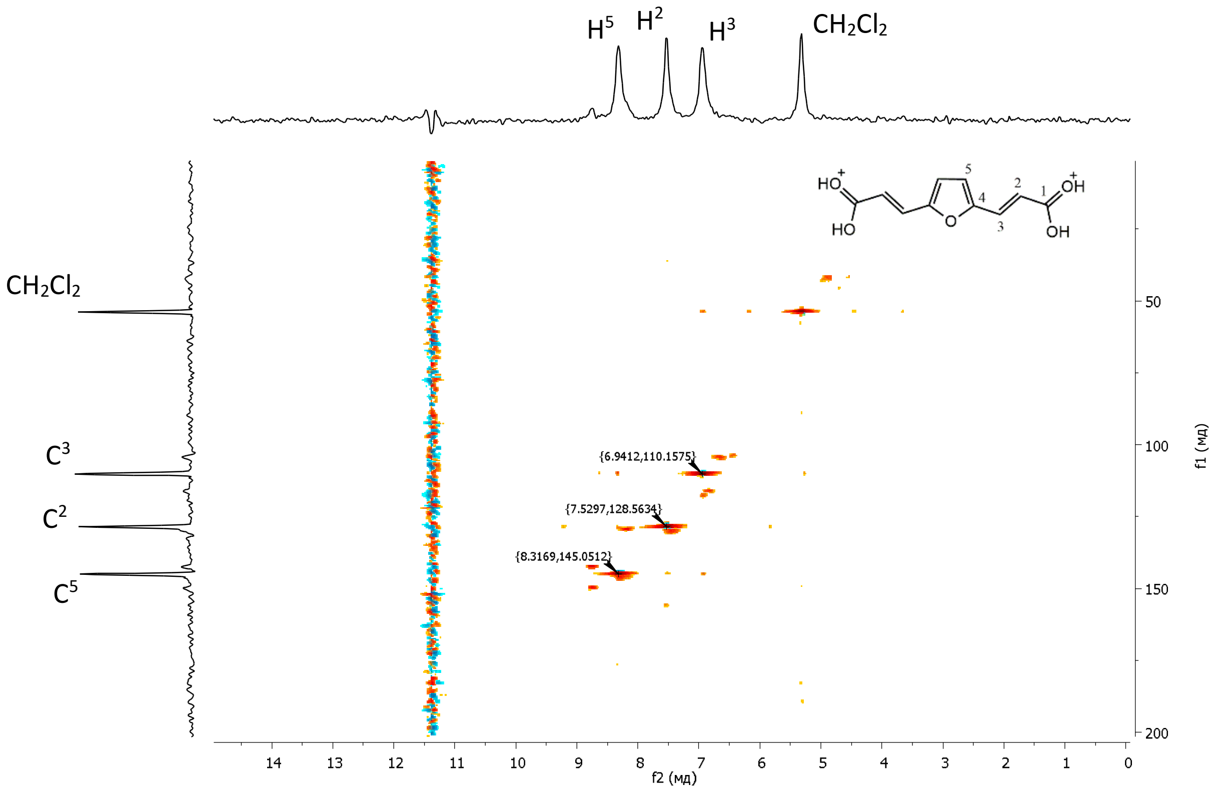


Figure S63. $^1$H–$^{13}$C HSQC spectrum of cation **Aa** in TFOH at room temperature ($CH_2Cl_2$ as internal standard).

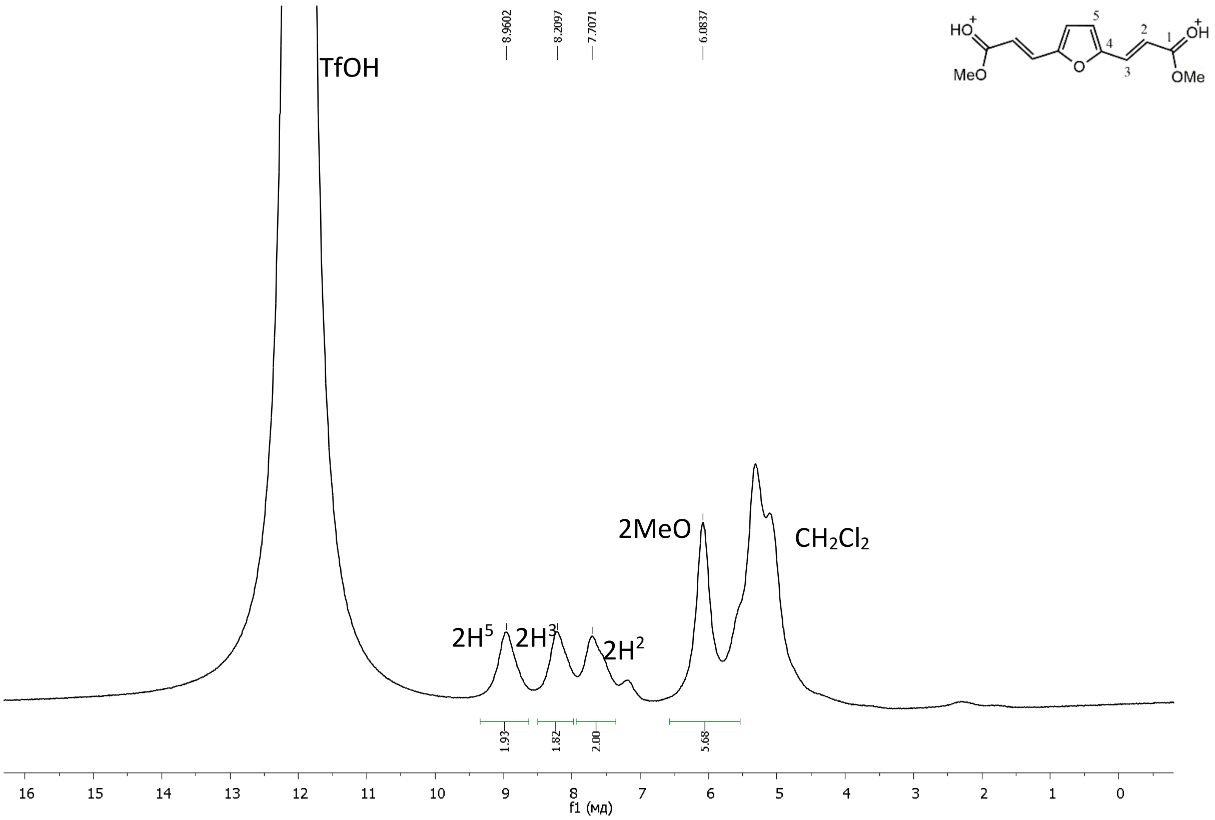


Figure S64. $^1$H NMR spectrum of cation **Ah** in TFOH at room temperature ($CH_2Cl_2$ as internal standard, 400 MHz).

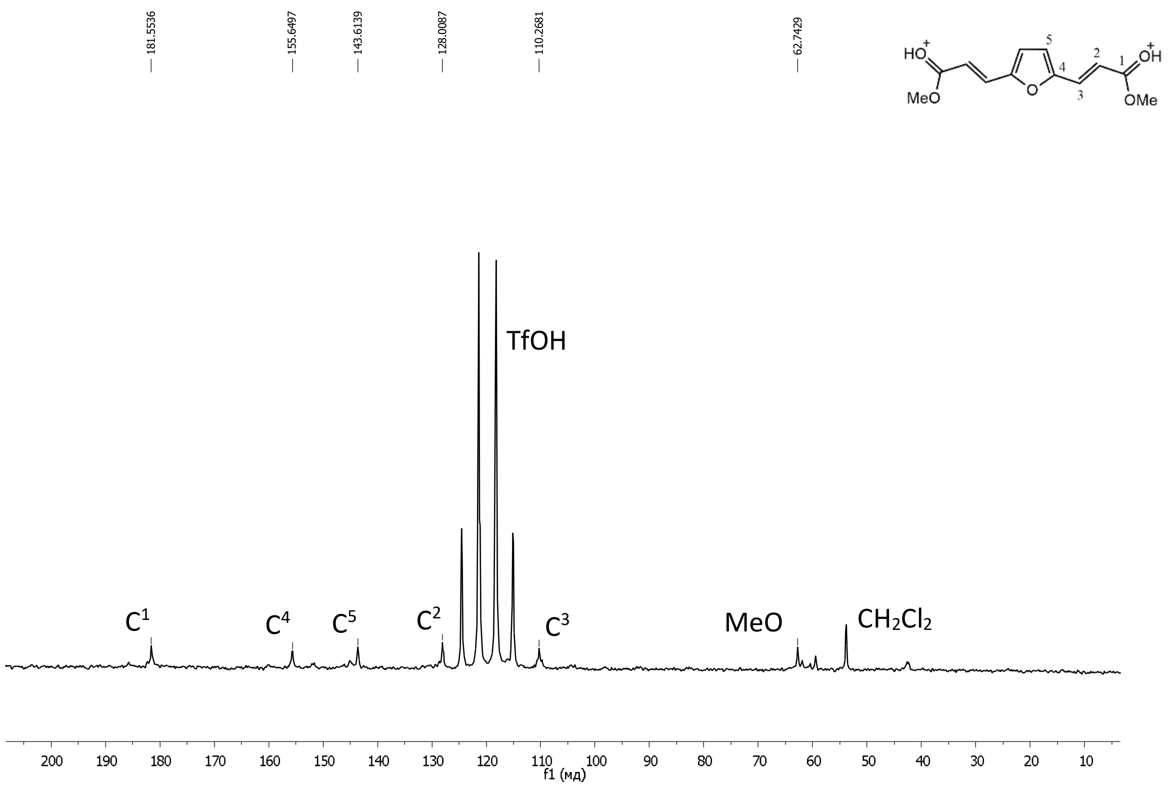


Figure S65. $^{13}C$ NMR spectrum of cation **Aa** in TfOH at room temperature ($CH_2Cl_2$ as internal standard, 100 MHz).

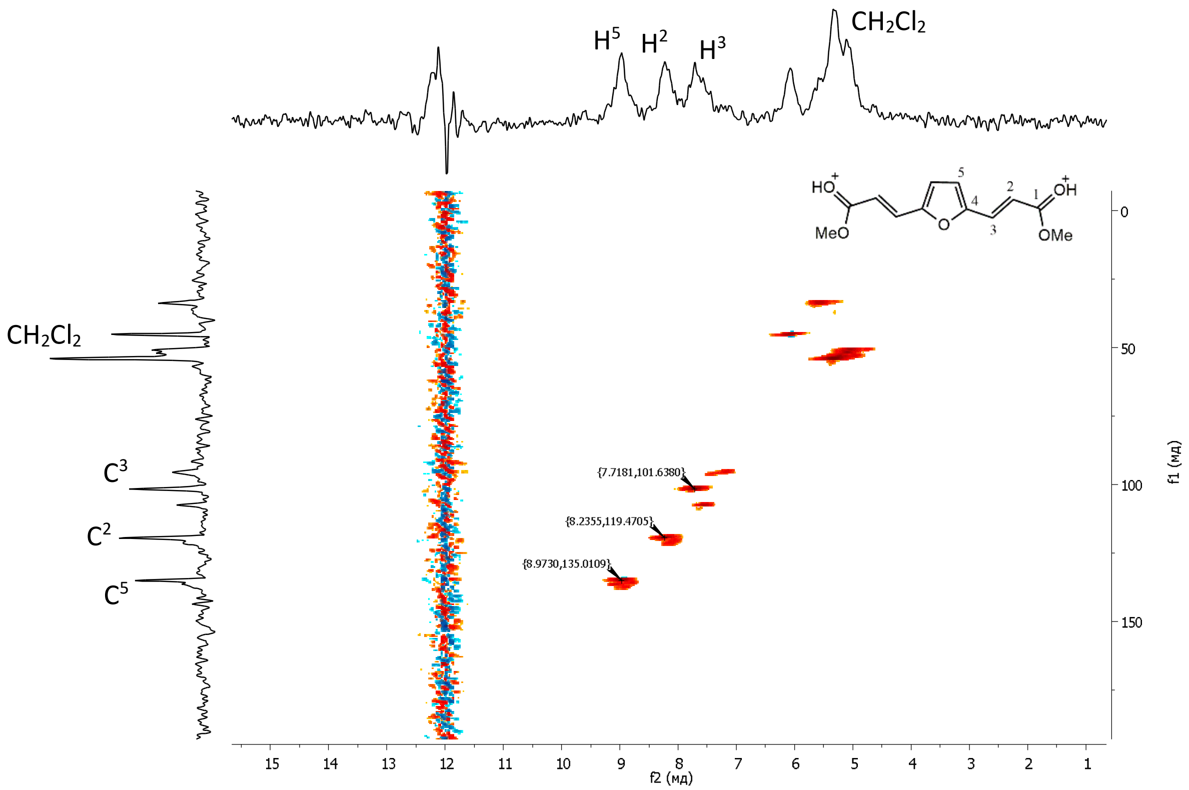


Figure S66. $^{1}H$–$^{13}C$ HSQC spectrum of cation **Ah** in TFOH at room temperature ($CH_2Cl_2$ as internal standard).

## 5. Data of DFT calculations of compounds 1 and species A-C

**Compound 1a**
**Energy E(B3LYP) = -496.19161622 h, $G^{298}$ = -496.110577 h, μ=4.70 D**

**Cartesian coordinates, Å**

| N | atom | x | y | z |
|---|---|---|---|---|
| 1 | C | -3.027947 | -1.366141 | 0.000000 |
| 2 | C | -2.224016 | -2.466232 | 0.000000 |
| 3 | C | -0.888102 | -1.983538 | 0.000000 |
| 4 | C | -0.958886 | -0.613335 | 0.000000 |
| 5 | O | -2.284495 | -0.233441 | 0.000000 |
| 6 | C | 0.000000 | 0.448866 | 0.000000 |
| 7 | C | 1.337883 | 0.289522 | 0.000000 |
| 8 | C | 2.289653 | 1.397185 | 0.000000 |
| 9 | O | 1.734516 | 2.624064 | 0.000000 |
| 10 | O | 3.502506 | 1.236274 | 0.000000 |
| 11 | H | -2.549587 | -3.495557 | 0.000000 |
| 12 | H | 0.017125 | -2.572778 | 0.000000 |
| 13 | H | -4.099510 | -1.233439 | 0.000000 |
| 14 | H | -0.418832 | 1.450835 | 0.000000 |
| 15 | H | 1.794946 | -0.693384 | 0.000000 |
| 16 | H | 2.464129 | 3.291187 | 0.000000 |

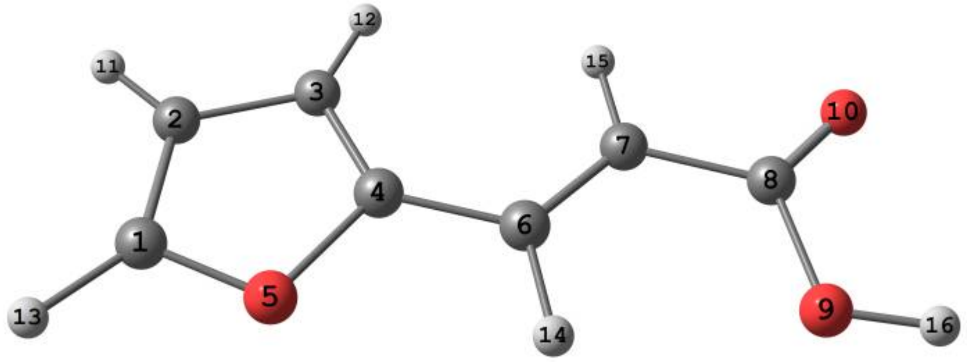

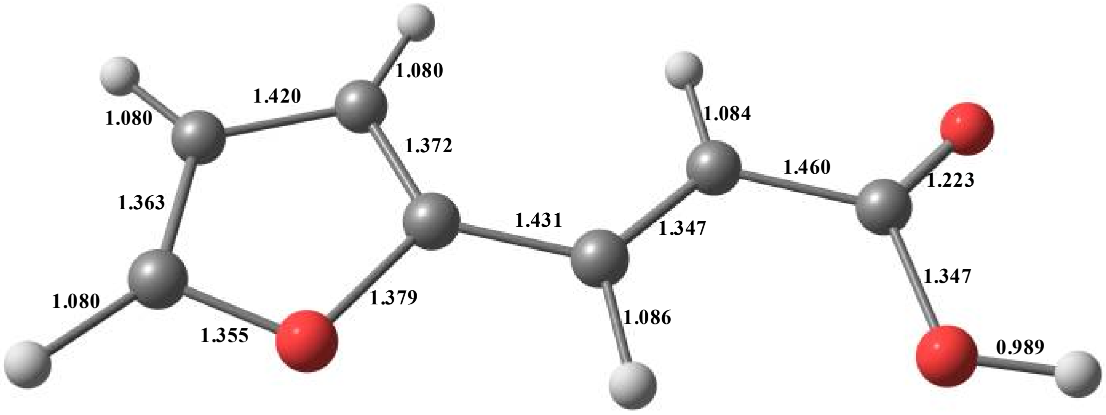


**Summary of Natural Population Analysis:**

| Atom | No | Natural Charge | Core | Valence | Rydberg | Total |
|---|---|---|---|---|---|---|
| C | 1 | 0.14732 | 1.99908 | 3.82860 | 0.02499 | 5.85268 |
| C | 2 | -0.30733 | 1.99916 | 4.28940 | 0.01877 | 6.30733 |
| C | 3 | -0.24174 | 1.99903 | 4.22559 | 0.01712 | 6.24174 |
| C | 4 | 0.23379 | 1.99893 | 3.74256 | 0.02472 | 5.76621 |
| O | 5 | -0.46982 | 1.99973 | 6.44562 | 0.02448 | 8.46982 |
| C | 6 | -0.15432 | 1.99904 | 4.13504 | 0.02025 | 6.15432 |
| C | 7 | -0.31694 | 1.99899 | 4.29750 | 0.02045 | 6.31694 |
| C | 8 | 0.78286 | 1.99936 | 3.17937 | 0.03841 | 5.21714 |
| O | 9 | -0.72431 | 1.99975 | 6.70199 | 0.02257 | 8.72431 |
| O | 10 | -0.68539 | 1.99975 | 6.65927 | 0.02637 | 8.68539 |
| H | 11 | 0.24677 | 0.00000 | 0.75174 | 0.00150 | 0.75323 |
| H | 12 | 0.24755 | 0.00000 | 0.75094 | 0.00151 | 0.75245 |
| H | 13 | 0.22557 | 0.00000 | 0.77299 | 0.00144 | 0.77443 |
| H | 14 | 0.24433 | 0.00000 | 0.75338 | 0.00229 | 0.75567 |
| H | 15 | 0.23138 | 0.00000 | 0.76636 | 0.00226 | 0.76862 |
| H | 16 | 0.54030 | 0.00000 | 0.45665 | 0.00305 | 0.45970 |
| * Total * | | 0.00000 | 19.99281 | 51.75702 | 0.25018 | 72.00000 |

**Aa**

**Energy E(B3LYP) = -496.618181837 h, $G^{298}$ = -496.526579 h, μ=8.55 D**

**Cartesian coordinates, Å**

| N | atom | x | y | z |
|---|---|---|---|---|
| 1 | C | -3.317050 | -0.560987 | -0.007732 |
| 2 | C | -3.252289 | 0.807760 | 0.007338 |
| 3 | C | -1.882605 | 1.135313 | 0.012156 |
| 4 | C | -1.183261 | -0.056741 | 0.000311 |
| 5 | O | -2.087054 | -1.101940 | -0.011663 |
| 6 | C | 0.181271 | -0.403138 | -0.000794 |
| 7 | C | 1.215630 | 0.487248 | -0.001413 |
| 8 | C | 2.561920 | 0.063782 | -0.000418 |
| 9 | O | 2.862023 | -1.201046 | 0.018913 |
| 10 | O | 3.478979 | 0.986465 | -0.016317 |
| 11 | H | -4.092403 | 1.485983 | 0.014379 |
| 12 | H | -1.447214 | 2.124812 | 0.024337 |
| 13 | H | -4.145838 | -1.255051 | -0.016987 |
| 14 | H | 0.394128 | -1.469853 | -0.002799 |
| 15 | H | 1.059464 | 1.559565 | -0.005482 |
| 16 | H | 3.848952 | -1.369610 | 0.012928 |
| 17 | H | 4.409633 | 0.616897 | -0.010523 |

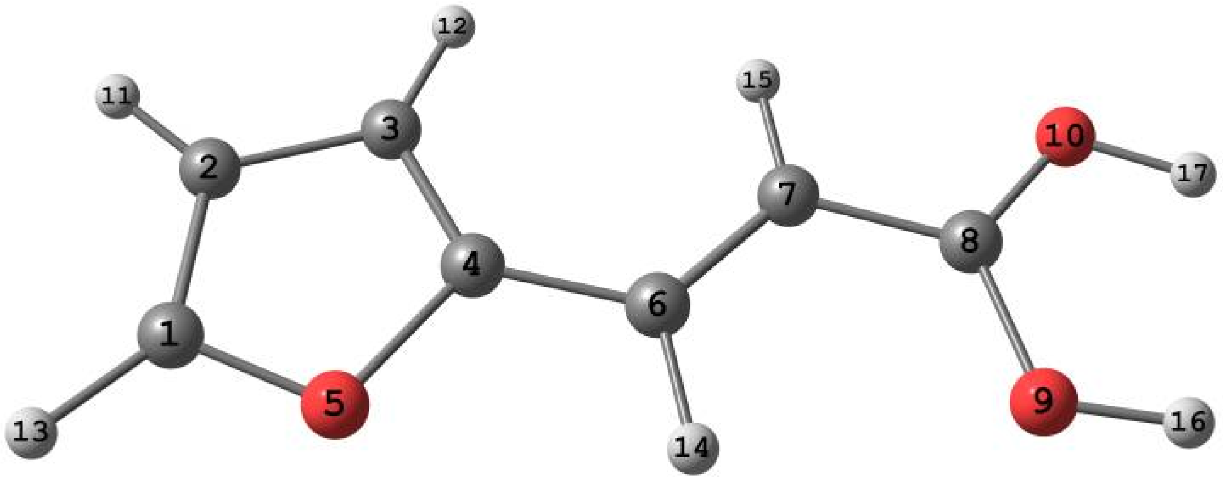

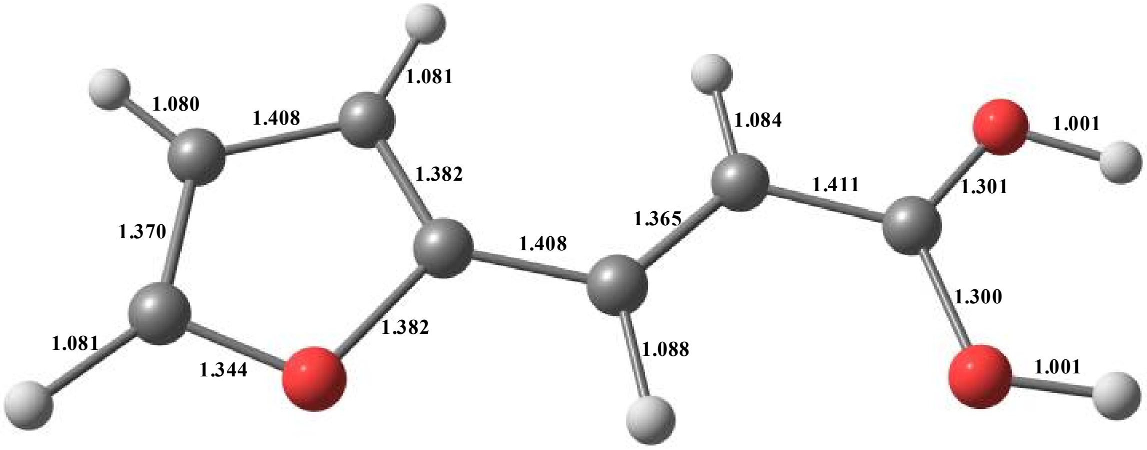


**Summary of Natural Population Analysis:**

| Atom | No | Natural Charge | Natural Population Core | Valence | Rydberg | Total |
|---|---|---|---|---|---|---|
| C | 1 | 0.20254 | 1.99911 | 3.77398 | 0.02437 | 5.79746 |
| C | 2 | -0.30190 | 1.99917 | 4.28414 | 0.01859 | 6.30190 |
| C | 3 | -0.18516 | 1.99904 | 4.16950 | 0.01662 | 6.18516 |
| C | 4 | 0.21780 | 1.99893 | 3.75934 | 0.02393 | 5.78220 |
| O | 5 | -0.45231 | 1.99972 | 6.42819 | 0.02440 | 8.45231 |
| C | 6 | -0.07691 | 1.99905 | 4.05898 | 0.01888 | 6.07691 |
| C | 7 | -0.35467 | 1.99901 | 4.33509 | 0.02057 | 6.35467 |
| C | 8 | 0.82651 | 1.99922 | 3.14489 | 0.02938 | 5.17349 |
| O | 9 | -0.64670 | 1.99970 | 6.62277 | 0.02423 | 8.64670 |
| O | 10 | -0.63609 | 1.99970 | 6.61199 | 0.02439 | 8.63609 |
| H | 11 | 0.25603 | 0.00000 | 0.74249 | 0.00148 | 0.74397 |
| H | 12 | 0.25687 | 0.00000 | 0.74169 | 0.00144 | 0.74313 |
| H | 13 | 0.23420 | 0.00000 | 0.76443 | 0.00137 | 0.76580 |
| H | 14 | 0.25691 | 0.00000 | 0.74100 | 0.00209 | 0.74309 |
| H | 15 | 0.25697 | 0.00000 | 0.74115 | 0.00189 | 0.74303 |
| H | 16 | 0.57425 | 0.00000 | 0.42304 | 0.00271 | 0.42575 |
| H | 17 | 0.57167 | 0.00000 | 0.42566 | 0.00267 | 0.42833 |
| * Total * | | 1.00000 | 19.99265 | 51.76833 | 0.23902 | 72.00000 |

**Ba**

**Energy E(B3LYP) = -497.007336659 h, $G^{298}$ = -496.906348 h, μ=7.35 D**

**Cartesian coordinates, Å**

| N | atom | x | y | z |
|---|---|---|---|---|
| 1 | C | 3.258420 | -0.501921 | -0.135622 |
| 2 | C | 3.099554 | 0.867914 | -0.387335 |
| 3 | C | 1.769372 | 1.135311 | -0.188885 |
| 4 | C | 1.141770 | -0.090732 | 0.185016 |
| 5 | O | 2.133381 | -1.082946 | 0.199758 |
| 6 | C | -0.110654 | -0.451767 | 0.501286 |
| 7 | C | -1.249070 | 0.499328 | 0.592692 |
| 8 | C | -2.511468 | 0.022422 | -0.050065 |
| 9 | O | -2.544681 | -1.155137 | -0.528300 |
| 10 | O | -3.477003 | 0.849887 | -0.061858 |
| 11 | H | 3.886554 | 1.549302 | -0.680344 |
| 12 | H | 1.268459 | 2.091272 | -0.294336 |
| 13 | H | 4.141532 | -1.134949 | -0.175888 |
| 14 | H | -0.288749 | -1.499107 | 0.747269 |
| 15 | H | -1.030739 | 1.495595 | 0.201742 |
| 16 | H | -3.437798 | -1.417157 | -0.938407 |
| 17 | H | -4.323638 | 0.497463 | -0.498420 |
| 18 | H | -1.496734 | 0.639817 | 1.659068 |

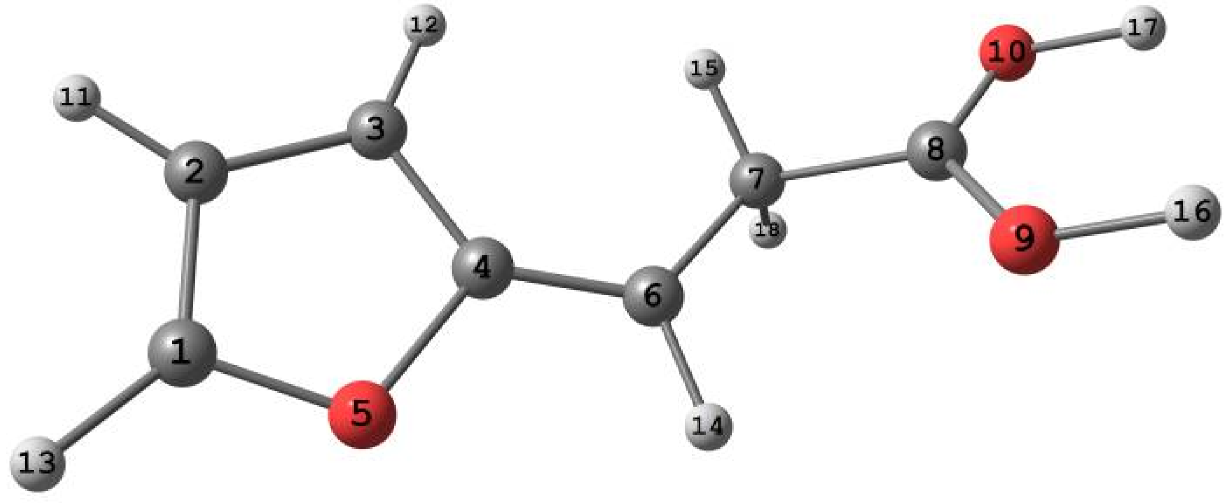

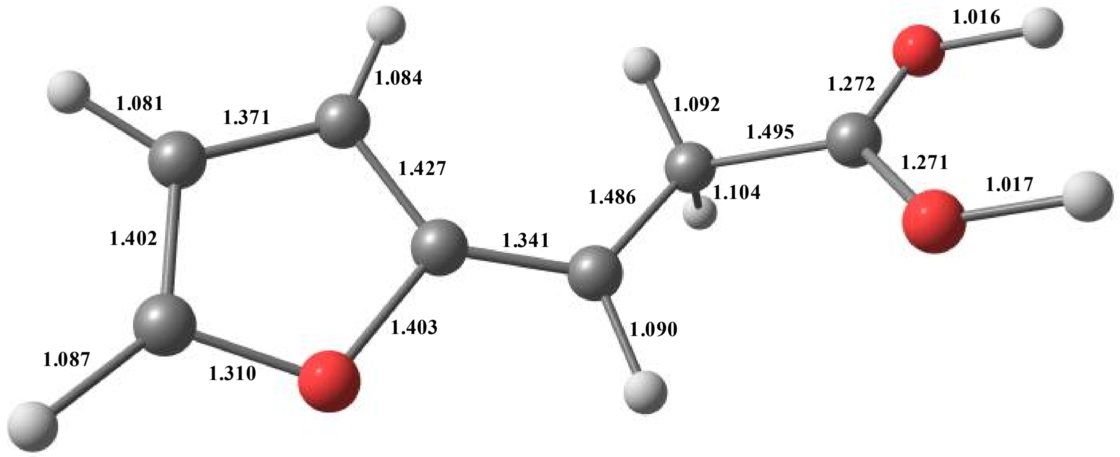


**Summary of Natural Population Analysis:**

| Atom | No | Natural Charge | Core | Valence | Rydberg | Total |
|---|---|---|---|---|---|---|
| C | 1 | 0.38115 | 1.99921 | 3.59599 | 0.02364 | 5.61885 |
| C | 2 | -0.27540 | 1.99916 | 4.25781 | 0.01843 | 6.27540 |
| C | 3 | -0.07009 | 1.99906 | 4.05440 | 0.01663 | 6.07009 |
| C | 4 | 0.22676 | 1.99882 | 3.75198 | 0.02244 | 5.77324 |
| O | 5 | -0.41138 | 1.99971 | 6.38624 | 0.02543 | 8.41138 |
| C | 6 | 0.02001 | 1.99897 | 3.96280 | 0.01821 | 5.97999 |
| C | 7 | -0.54328 | 1.99911 | 4.52438 | 0.01979 | 6.54328 |
| C | 8 | 0.94165 | 1.99944 | 3.02790 | 0.03101 | 5.05835 |
| O | 9 | -0.59868 | 1.99967 | 6.57423 | 0.02478 | 8.59868 |
| O | 10 | -0.59553 | 1.99967 | 6.57079 | 0.02507 | 8.59553 |
| H | 11 | 0.28358 | 0.00000 | 0.71504 | 0.00138 | 0.71642 |
| H | 12 | 0.28120 | 0.00000 | 0.71722 | 0.00158 | 0.71880 |
| H | 13 | 0.26204 | 0.00000 | 0.73676 | 0.00120 | 0.73796 |
| H | 14 | 0.28167 | 0.00000 | 0.71636 | 0.00198 | 0.71833 |
| H | 15 | 0.29133 | 0.00000 | 0.70703 | 0.00164 | 0.70867 |
| H | 16 | 0.60282 | 0.00000 | 0.39464 | 0.00254 | 0.39718 |
| H | 17 | 0.59948 | 0.00000 | 0.39794 | 0.00258 | 0.40052 |
| H | 18 | 0.32266 | 0.00000 | 0.67576 | 0.00158 | 0.67734 |
| * Total * | | 2.00000 | 19.99281 | 51.76727 | 0.23992 | 72.00000 |

Core, Valence, Rydberg and Total are printed under the spanning heading "Natural Population".

**Compound 1g**
**Energy E(B3LYP) = -535.494711806 h, $G^{298}$ = -535.387039 h, μ=4.49 D**

**Cartesian coordinates, Å**

| N | atom | x | y | z |
|---|---|---|---|---|
| 1 | C | 3.654663 | -0.789203 | 0.000338 |
| 2 | C | 3.752951 | 0.569535 | -0.000016 |
| 3 | C | 2.421545 | 1.065770 | -0.000371 |
| 4 | C | 1.589160 | -0.024609 | -0.000099 |
| 5 | O | 2.354477 | -1.171886 | 0.000496 |
| 6 | C | 0.170757 | -0.218445 | -0.000174 |
| 7 | C | -0.751044 | 0.763225 | -0.000712 |
| 8 | C | -2.195979 | 0.534422 | -0.000080 |
| 9 | O | -2.550297 | -0.765577 | -0.000874 |
| 10 | C | -3.963929 | -1.042715 | 0.000092 |
| 11 | O | -3.016884 | 1.439070 | 0.001160 |
| 12 | H | 4.666974 | 1.144003 | -0.000098 |
| 13 | H | 2.112975 | 2.100772 | -0.000781 |
| 14 | H | 4.385631 | -1.583744 | 0.000656 |
| 15 | H | -0.153134 | -1.254943 | 0.000365 |
| 16 | H | -0.468562 | 1.810045 | -0.001180 |
| 17 | H | -4.432215 | -0.626482 | 0.889606 |
| 18 | H | -4.433555 | -0.625521 | -0.888265 |
| 19 | H | -4.045219 | -2.124867 | -0.000422 |

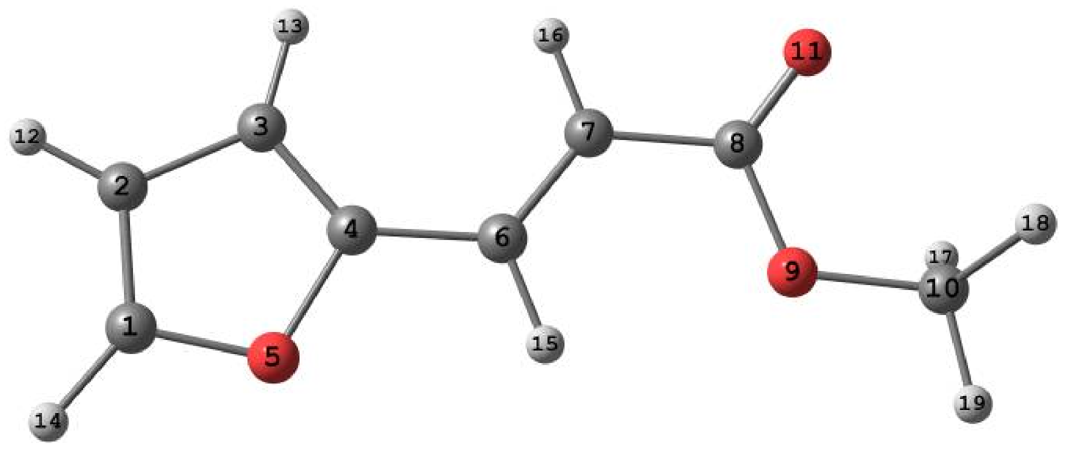

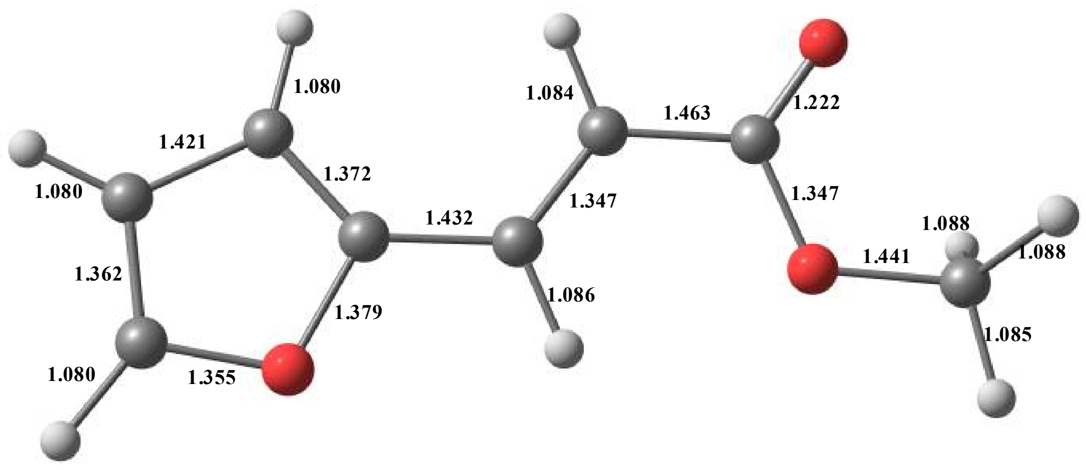


**Summary of Natural Population Analysis:**

| Atom | No | Natural Charge | Core | Valence | Rydberg | Total |
|---|---|---|---|---|---|---|
| C | 1 | 0.14555 | 1.99908 | 3.83036 | 0.02501 | 5.85445 |
| C | 2 | -0.30739 | 1.99916 | 4.28946 | 0.01876 | 6.30739 |
| C | 3 | -0.24383 | 1.99902 | 4.22771 | 0.01709 | 6.24383 |
| C | 4 | 0.23528 | 1.99893 | 3.74128 | 0.02452 | 5.76472 |
| O | 5 | -0.47044 | 1.99973 | 6.44639 | 0.02433 | 8.47044 |
| C | 6 | -0.15582 | 1.99903 | 4.13735 | 0.01944 | 6.15582 |
| C | 7 | -0.30778 | 1.99900 | 4.28833 | 0.02045 | 6.30778 |
| C | 8 | 0.78951 | 1.99931 | 3.17455 | 0.03664 | 5.21049 |
| O | 9 | -0.56433 | 1.99971 | 6.54068 | 0.02394 | 8.56433 |
| C | 10 | -0.21221 | 1.99923 | 4.19888 | 0.01411 | 6.21221 |
| O | 11 | -0.67152 | 1.99975 | 6.64624 | 0.02553 | 8.67152 |
| H | 12 | 0.24643 | 0.00000 | 0.75208 | 0.00150 | 0.75357 |
| H | 13 | 0.24716 | 0.00000 | 0.75133 | 0.00151 | 0.75284 |
| H | 14 | 0.22521 | 0.00000 | 0.77334 | 0.00145 | 0.77479 |
| H | 15 | 0.24375 | 0.00000 | 0.75396 | 0.00229 | 0.75625 |
| H | 16 | 0.23134 | 0.00000 | 0.76651 | 0.00215 | 0.76866 |
| H | 17 | 0.18806 | 0.00000 | 0.80984 | 0.00210 | 0.81194 |
| H | 18 | 0.18808 | 0.00000 | 0.80982 | 0.00210 | 0.81192 |
| H | 19 | 0.19296 | 0.00000 | 0.80570 | 0.00135 | 0.80704 |
| * Total * | | 0.00000 | 21.99195 | 57.74380 | 0.26424 | 80.00000 |

**Ag**

**Energy E(B3LYP) = -535.909938855 h, $G^{298}$ = -535.79194 h, μ=6.39 D**

**Cartesian coordinates, Å**

| N | atom | x | y | z |
|---|---|---|---|---|
| 1 | C | 3.681990 | -0.772453 | 0.000535 |
| 2 | C | 3.763278 | 0.596304 | -0.000275 |
| 3 | C | 2.436657 | 1.067150 | -0.000519 |
| 4 | C | 1.613783 | -0.044387 | -0.000024 |
| 5 | O | 2.402890 | -1.179576 | 0.000234 |
| 6 | C | 0.222178 | -0.248223 | 0.000099 |
| 7 | C | -0.718432 | 0.742032 | -0.000081 |
| 8 | C | -2.101234 | 0.458342 | -0.000063 |
| 9 | O | -2.504275 | -0.776231 | -0.000711 |
| 10 | C | -3.907734 | -1.141872 | -0.000090 |
| 11 | O | -2.903702 | 1.485679 | 0.000784 |
| 12 | H | 4.670926 | 1.181039 | -0.000549 |
| 13 | H | 2.108563 | 2.097250 | -0.001050 |
| 14 | H | 4.433635 | -1.549608 | 0.000994 |
| 15 | H | -0.097881 | -1.287737 | 0.000296 |
| 16 | H | -3.873545 | 1.335166 | 0.000318 |
| 17 | H | -0.455278 | 1.793313 | -0.000147 |
| 18 | H | -4.388373 | -0.762545 | 0.900589 |
| 19 | H | -4.389376 | -0.761921 | -0.899969 |
| 20 | H | -3.910893 | -2.225294 | -0.000429 |

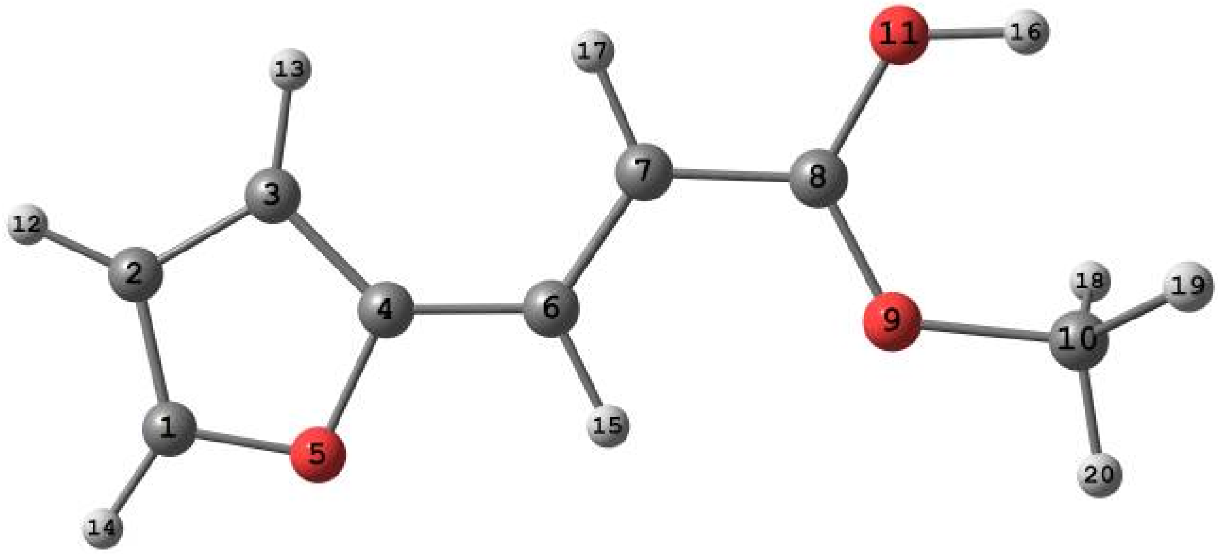

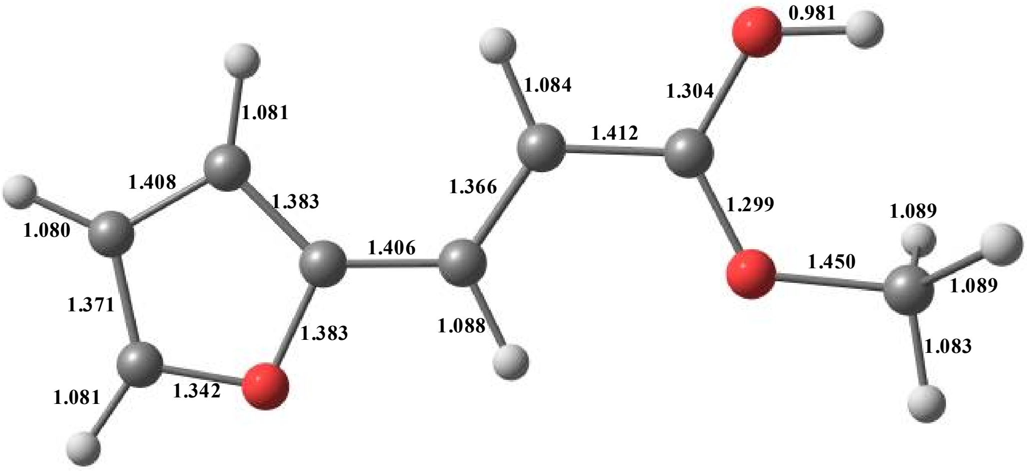


**Summary of Natural Population Analysis:**

| Atom | No | Natural Charge | Natural Population: Core | Valence | Rydberg | Total |
|---|---|---|---|---|---|---|
| C | 1 | 0.20571 | 1.99912 | 3.77080 | 0.02437 | 5.79429 |
| C | 2 | -0.30155 | 1.99917 | 4.28380 | 0.01859 | 6.30155 |
| C | 3 | -0.18296 | 1.99904 | 4.16734 | 0.01658 | 6.18296 |
| C | 4 | 0.21843 | 1.99893 | 3.75887 | 0.02377 | 5.78157 |
| O | 5 | -0.45159 | 1.99972 | 6.42738 | 0.02448 | 8.45159 |
| C | 6 | -0.07519 | 1.99904 | 4.05725 | 0.01889 | 6.07519 |
| C | 7 | -0.34671 | 1.99902 | 4.32651 | 0.02118 | 6.34671 |
| C | 8 | 0.83186 | 1.99914 | 3.14134 | 0.02766 | 5.16814 |
| O | 9 | -0.48790 | 1.99965 | 6.46419 | 0.02406 | 8.48790 |
| C | 10 | -0.23111 | 1.99920 | 4.21921 | 0.01269 | 6.23111 |
| O | 11 | -0.63119 | 1.99969 | 6.60751 | 0.02399 | 8.63119 |
| H | 12 | 0.25641 | 0.00000 | 0.74211 | 0.00147 | 0.74359 |
| H | 13 | 0.25729 | 0.00000 | 0.74127 | 0.00145 | 0.74271 |
| H | 14 | 0.23459 | 0.00000 | 0.76405 | 0.00136 | 0.76541 |
| H | 15 | 0.25715 | 0.00000 | 0.74035 | 0.00250 | 0.74285 |
| H | 16 | 0.55556 | 0.00000 | 0.44179 | 0.00264 | 0.44444 |
| H | 17 | 0.25827 | 0.00000 | 0.73983 | 0.00190 | 0.74173 |
| H | 18 | 0.20496 | 0.00000 | 0.79334 | 0.00170 | 0.79504 |
| H | 19 | 0.20500 | 0.00000 | 0.79330 | 0.00170 | 0.79500 |
| H | 20 | 0.22295 | 0.00000 | 0.77584 | 0.00121 | 0.77705 |
| * Total * | | 1.00000 | 21.99172 | 57.75609 | 0.25219 | 80.00000 |

**Bg**
**Energy E(B3LYP) = -536.298620401 h, $G^{298}$ = -536.169898 h, μ=4.10 D**

**Cartesian coordinates, Å**

| N | atom | x | y | z |
|---|---|---|---|---|
| 1 | C | 3.567431 | 0.731844 | 0.047186 |
| 2 | C | 3.524406 | -0.519977 | 0.677754 |
| 3 | C | 2.242934 | -0.979121 | 0.514343 |
| 4 | C | 1.527193 | 0.013046 | -0.220941 |
| 5 | O | 2.417991 | 1.065454 | -0.482752 |
| 6 | C | 0.271761 | 0.127290 | -0.677970 |
| 7 | C | -0.756634 | -0.937434 | -0.546361 |
| 8 | C | -2.106636 | -0.456469 | -0.112018 |
| 9 | O | -2.241590 | 0.780538 | 0.135422 |
| 10 | C | -3.506583 | 1.399168 | 0.563015 |
| 11 | O | -2.993185 | -1.366117 | -0.034189 |
| 12 | H | 4.351214 | -0.998620 | 1.184641 |
| 13 | H | 1.829346 | -1.915850 | 0.871959 |
| 14 | H | 4.382231 | 1.443414 | -0.064082 |
| 15 | H | 0.009922 | 1.036152 | -1.221050 |
| 16 | H | -0.469021 | -1.752063 | 0.123712 |
| 17 | H | -3.928196 | -1.175410 | 0.253884 |
| 18 | H | -0.907468 | -1.398799 | -1.536484 |
| 19 | H | -4.249241 | 1.245942 | -0.216532 |
| 20 | H | -3.805502 | 0.958276 | 1.511163 |
| 21 | H | -3.262247 | 2.447867 | 0.674881 |

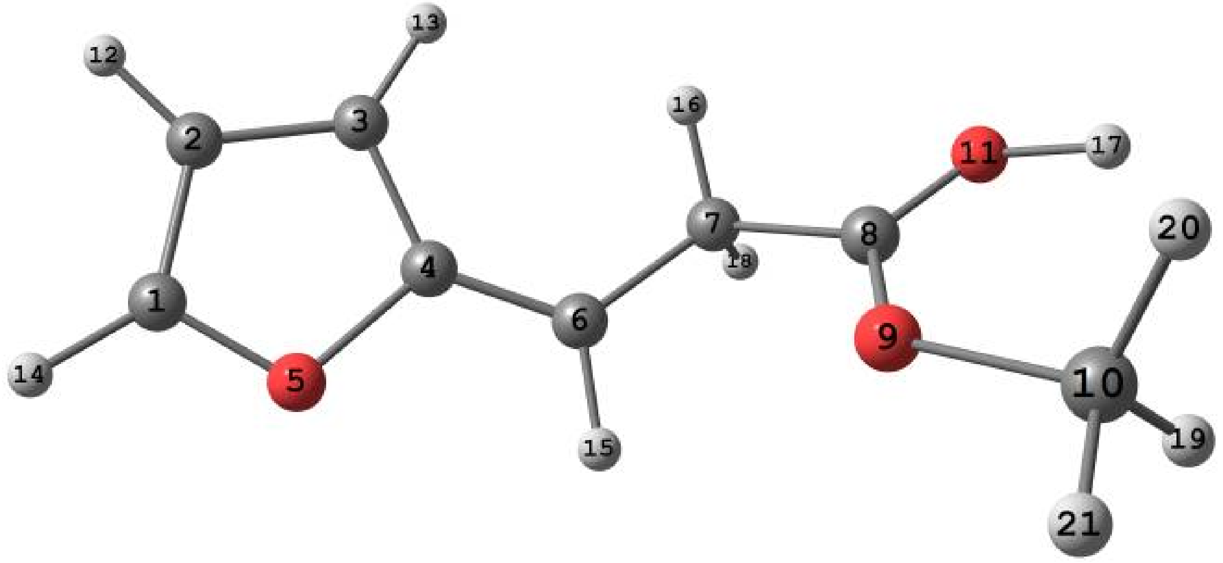

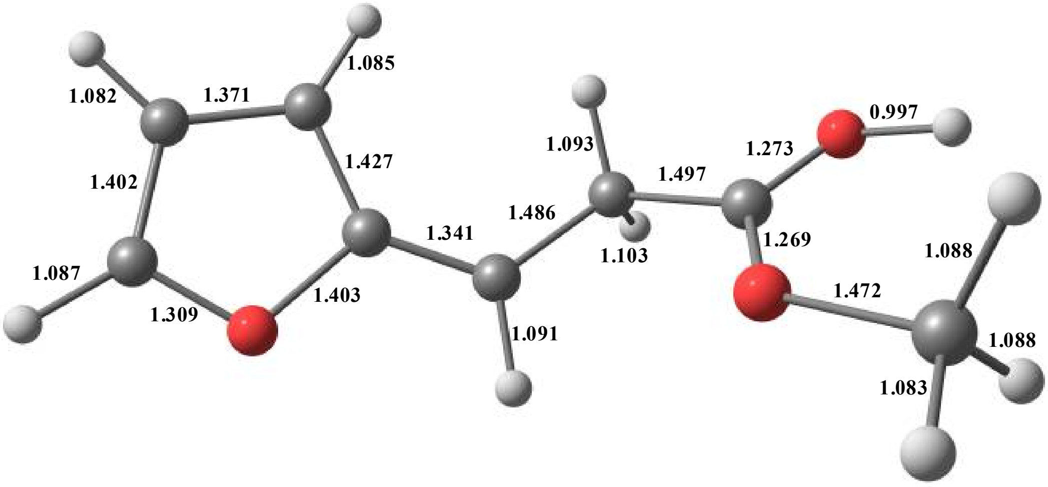


**Summary of Natural Population Analysis:**

| Atom | No | Natural Charge | Natural Population Core | Valence | Rydberg | Total |
|---|---|---|---|---|---|---|
| C | 1 | 0.38226 | 1.99921 | 3.59486 | 0.02366 | 5.61774 |
| C | 2 | -0.27501 | 1.99916 | 4.25741 | 0.01844 | 6.27501 |
| C | 3 | -0.07047 | 1.99906 | 4.05475 | 0.01666 | 6.07047 |
| C | 4 | 0.22751 | 1.99881 | 3.75102 | 0.02266 | 5.77249 |
| O | 5 | -0.41117 | 1.99971 | 6.38579 | 0.02567 | 8.41117 |
| C | 6 | 0.01836 | 1.99897 | 3.96429 | 0.01839 | 5.98164 |
| C | 7 | -0.53610 | 1.99912 | 4.51683 | 0.02015 | 6.53610 |
| C | 8 | 0.95159 | 1.99934 | 3.02027 | 0.02880 | 5.04841 |
| O | 9 | -0.44403 | 1.99961 | 6.42181 | 0.02261 | 8.44403 |
| C | 10 | -0.22775 | 1.99920 | 4.21462 | 0.01393 | 6.22775 |
| O | 11 | -0.59676 | 1.99965 | 6.57287 | 0.02425 | 8.59676 |
| H | 12 | 0.28377 | 0.00000 | 0.71484 | 0.00138 | 0.71623 |
| H | 13 | 0.28144 | 0.00000 | 0.71697 | 0.00159 | 0.71856 |
| H | 14 | 0.26217 | 0.00000 | 0.73663 | 0.00120 | 0.73783 |
| H | 15 | 0.28171 | 0.00000 | 0.71592 | 0.00237 | 0.71829 |
| H | 16 | 0.29327 | 0.00000 | 0.70502 | 0.00171 | 0.70673 |
| H | 17 | 0.58933 | 0.00000 | 0.40814 | 0.00253 | 0.41067 |
| H | 18 | 0.32040 | 0.00000 | 0.67795 | 0.00165 | 0.67960 |
| H | 19 | 0.21887 | 0.00000 | 0.77981 | 0.00132 | 0.78113 |
| H | 20 | 0.21883 | 0.00000 | 0.77985 | 0.00132 | 0.78117 |
| H | 21 | 0.23179 | 0.00000 | 0.76719 | 0.00101 | 0.76821 |
| * Total * | | 2.00000 | 21.99184 | 57.75685 | 0.25131 | 80.00000 |

**Compound 1e**
**Energy E(B3LYP) = -762.280625947 h, $G^{298}$ = -762.160726 h, μ=6.38 D**

**Cartesian coordinates, Å**

| N | atom | x | y | z |
|---|---|---|---|---|
| 1 | C | 1.104502 | 0.357988 | 0.000128 |
| 2 | C | 0.703227 | 1.676870 | 0.000188 |
| 3 | C | -0.703271 | 1.676852 | -0.000065 |
| 4 | C | -1.104497 | 0.357952 | -0.000398 |
| 5 | O | 0.000000 | -0.448606 | -0.000276 |
| 6 | C | -2.374732 | -0.300209 | -0.000628 |
| 7 | C | -3.567140 | 0.322248 | -0.000126 |
| 8 | C | -4.819476 | -0.440717 | -0.000016 |
| 9 | O | -4.919658 | -1.656507 | -0.000803 |
| 10 | C | 2.374726 | -0.300174 | 0.000411 |
| 11 | C | 3.567135 | 0.322283 | -0.000016 |
| 12 | C | 4.819532 | -0.440717 | 0.000287 |
| 13 | O | 5.893364 | 0.372512 | -0.001273 |
| 14 | O | -5.893346 | 0.372547 | 0.000910 |
| 15 | O | 4.919635 | -1.656503 | 0.001626 |
| 16 | H | 1.354344 | 2.538797 | 0.000478 |
| 17 | H | -1.354400 | 2.538768 | -0.000059 |
| 18 | H | -2.345710 | -1.386908 | -0.001035 |
| 19 | H | -3.652732 | 1.402989 | 0.000531 |
| 20 | H | 2.345703 | -1.386873 | 0.000911 |
| 21 | H | 3.652761 | 1.403022 | -0.000779 |
| 22 | H | -6.710229 | -0.185795 | 0.000882 |
| 23 | H | 6.710275 | -0.185797 | -0.000993 |

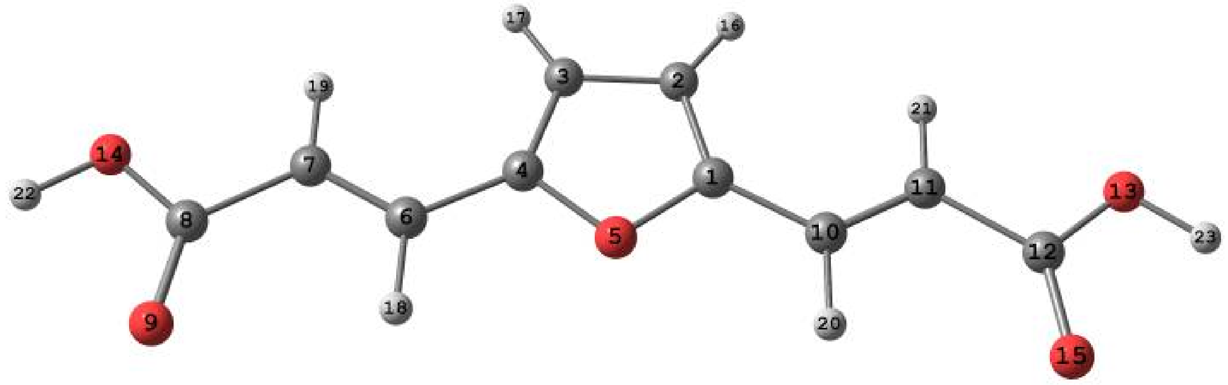

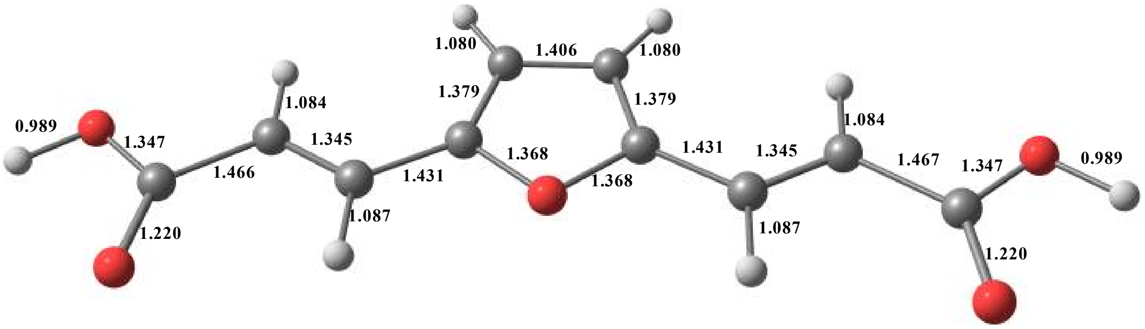


**Summary of Natural Population Analysis:**

| Atom | No | Natural Charge | Core | Valence | Rydberg | Total |
|---|---|---|---|---|---|---|
| C | 1 | 0.27035 | 1.99894 | 3.70686 | 0.02385 | 5.72965 |
| C | 2 | -0.23927 | 1.99904 | 4.22322 | 0.01701 | 6.23927 |
| C | 3 | -0.23926 | 1.99904 | 4.22321 | 0.01701 | 6.23926 |
| C | 4 | 0.27035 | 1.99894 | 3.70686 | 0.02385 | 5.72965 |
| O | 5 | -0.45841 | 1.99970 | 6.43478 | 0.02393 | 8.45841 |
| C | 6 | -0.16993 | 1.99906 | 4.14710 | 0.02376 | 6.16993 |
| C | 7 | -0.31097 | 1.99887 | 4.28284 | 0.02927 | 6.31097 |
| C | 8 | 0.82744 | 1.99941 | 3.13622 | 0.03692 | 5.17256 |
| O | 9 | -0.69516 | 1.99975 | 6.66400 | 0.03142 | 8.69516 |
| C | 10 | -0.16996 | 1.99906 | 4.14714 | 0.02376 | 6.16996 |
| C | 11 | -0.31094 | 1.99887 | 4.28281 | 0.02927 | 6.31094 |
| C | 12 | 0.82745 | 1.99941 | 3.13621 | 0.03692 | 5.17255 |
| O | 13 | -0.72290 | 1.99975 | 6.69725 | 0.02590 | 8.72290 |
| O | 14 | -0.72292 | 1.99975 | 6.69727 | 0.02590 | 8.72292 |
| O | 15 | -0.69516 | 1.99975 | 6.66400 | 0.03142 | 8.69516 |
| H | 16 | 0.25191 | 0.00000 | 0.74656 | 0.00153 | 0.74809 |
| H | 17 | 0.25191 | 0.00000 | 0.74656 | 0.00153 | 0.74809 |
| H | 18 | 0.24711 | 0.00000 | 0.75068 | 0.00221 | 0.75289 |
| H | 19 | 0.23240 | 0.00000 | 0.76560 | 0.00200 | 0.76760 |
| H | 20 | 0.24711 | 0.00000 | 0.75068 | 0.00221 | 0.75289 |
| H | 21 | 0.23240 | 0.00000 | 0.76561 | 0.00200 | 0.76760 |
| H | 22 | 0.53960 | 0.00000 | 0.45745 | 0.00295 | 0.46040 |
| H | 23 | 0.53961 | 0.00000 | 0.45744 | 0.00295 | 0.46039 |
| * Total * | | 0.00274 | 29.98936 | 77.59035 | 0.41755 | 107.99726 |

Core, Valence, Rydberg and Total are printed under the spanning header "Natural Population".

**Ae**

**Energy E(B3LYP) = -763.12469868 h, $G^{298}$ = -762.984781 h, μ=4.31 D**

**Cartesian coordinates, Å**

| N | atom | x | y | z |
|---|---|---|---|---|
| 1 | C | -1.097662 | 0.391170 | -0.000629 |
| 2 | C | -0.698742 | 1.718785 | -0.001036 |
| 3 | C | 0.698746 | 1.718784 | -0.000960 |
| 4 | C | 1.097661 | 0.391167 | -0.000503 |
| 5 | O | -0.000001 | -0.414992 | -0.000341 |
| 6 | C | 2.359204 | -0.260455 | -0.000088 |
| 7 | C | 3.553581 | 0.381253 | 0.000224 |
| 8 | C | 4.784350 | -0.337673 | 0.000386 |
| 9 | O | 4.782355 | -1.630375 | -0.000810 |
| 10 | C | -2.359206 | -0.260448 | -0.000376 |
| 11 | C | -3.553583 | 0.381259 | 0.000265 |
| 12 | C | -4.784351 | -0.337673 | 0.000398 |
| 13 | O | -5.873687 | 0.360056 | 0.001539 |
| 14 | O | 5.873683 | 0.360062 | 0.001722 |
| 15 | O | -4.782349 | -1.630375 | -0.000483 |
| 16 | H | -1.352022 | 2.580297 | -0.001449 |
| 17 | H | 1.352029 | 2.580294 | -0.001290 |
| 18 | H | 2.324942 | -1.347665 | 0.000098 |
| 19 | H | 3.641212 | 1.462131 | 0.000426 |
| 20 | H | -2.324952 | -1.347659 | -0.000677 |
| 21 | H | -3.641216 | 1.462136 | 0.000817 |
| 22 | H | 6.707506 | -0.200657 | 0.001705 |
| 23 | H | -5.703770 | -2.030120 | -0.000068 |
| 24 | H | 5.703778 | -2.030118 | -0.000293 |
| 25 | H | -6.707507 | -0.200668 | 0.001618 |

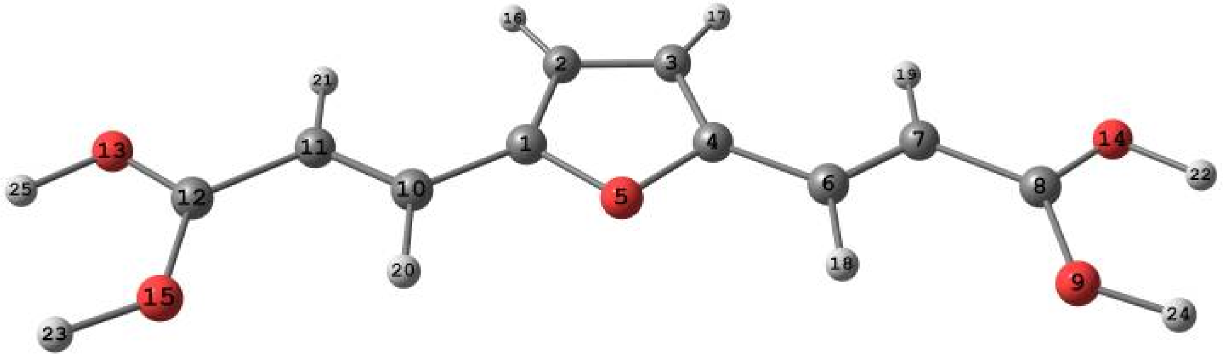

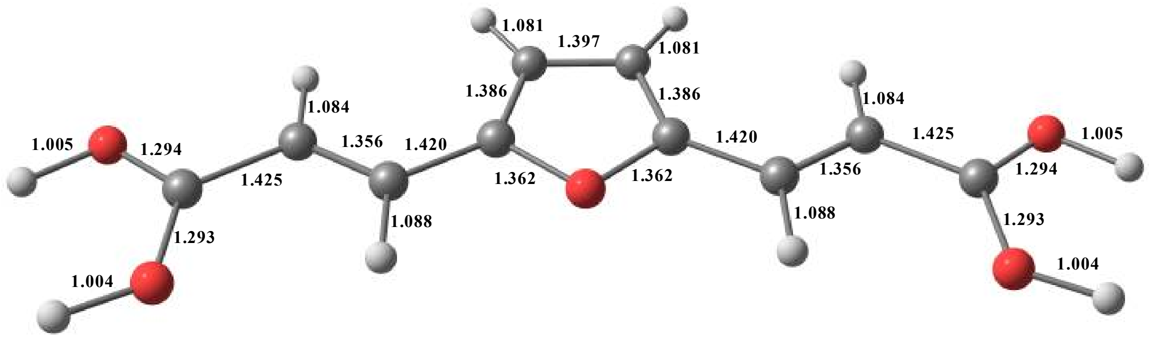


**Summary of Natural Population Analysis:**

| Atom | No | Natural Charge | Core | Valence | Rydberg | Total |
|---|---|---|---|---|---|---|
| C | 1 | 0.27527 | 1.99897 | 3.70058 | 0.02518 | 5.72473 |
| C | 2 | -0.20378 | 1.99904 | 4.18635 | 0.01839 | 6.20378 |
| C | 3 | -0.20337 | 1.99904 | 4.18613 | 0.01820 | 6.20337 |
| C | 4 | 0.28100 | 1.99896 | 3.69565 | 0.02438 | 5.71900 |
| O | 5 | -0.43646 | 1.99970 | 6.41200 | 0.02477 | 8.43646 |
| C | 6 | -0.13737 | 1.99914 | 4.10038 | 0.03784 | 6.13737 |
| C | 7 | -0.24064 | 1.99889 | 4.22150 | 0.02025 | 6.24064 |
| C | 8 | 0.86035 | 1.99936 | 3.11423 | 0.02605 | 5.13965 |
| O | 9 | -0.64263 | 1.99970 | 6.61432 | 0.02862 | 8.64263 |
| C | 10 | -0.12436 | 1.99893 | 4.08694 | 0.03849 | 6.12436 |
| C | 11 | -0.24578 | 1.99890 | 4.22467 | 0.02222 | 6.24578 |
| C | 12 | 0.85978 | 1.99936 | 3.11434 | 0.02652 | 5.14022 |
| O | 13 | -0.63324 | 1.99971 | 6.60346 | 0.03007 | 8.63324 |
| O | 14 | -0.63350 | 1.99971 | 6.60358 | 0.03022 | 8.63350 |
| O | 15 | -0.64266 | 1.99970 | 6.61436 | 0.02860 | 8.64266 |
| H | 16 | 0.26459 | 0.00000 | 0.73374 | 0.00167 | 0.73541 |
| H | 17 | 0.26455 | 0.00000 | 0.73380 | 0.00165 | 0.73545 |
| H | 18 | 0.26282 | 0.00000 | 0.73510 | 0.00207 | 0.73718 |
| H | 19 | 0.25190 | 0.00000 | 0.74412 | 0.00399 | 0.74810 |
| H | 20 | 0.26133 | 0.00000 | 0.73629 | 0.00238 | 0.73867 |
| H | 21 | 0.25296 | 0.00000 | 0.74327 | 0.00377 | 0.74704 |
| H | 22 | 0.57884 | 0.00000 | 0.41840 | 0.00276 | 0.42116 |
| H | 23 | 0.58115 | 0.00000 | 0.41614 | 0.00270 | 0.41885 |
| H | 24 | 0.58116 | 0.00000 | 0.41614 | 0.00271 | 0.41884 |
| H | 25 | 0.57881 | 0.00000 | 0.41840 | 0.00279 | 0.42119 |
| * Total * | | 2.01073 | 29.98911 | 77.57388 | 0.42628 | 107.98927 |

**Be**

**Energy E(B3LYP) = -763.503051513 h, $G^{298}$ = -763.354135 h, μ=4.09 D**

**Cartesian coordinates, Å**

| N | atom | x | y | z |
|---|---|---|---|---|
| 1 | C | 1.104910 | 0.296007 | 0.246846 |
| 2 | C | 0.702204 | 1.620823 | -0.093864 |
| 3 | C | -0.662042 | 1.617865 | -0.199209 |
| 4 | C | -1.076190 | 0.296782 | 0.073594 |
| 5 | O | -0.043398 | -0.487116 | 0.337223 |
| 6 | C | -2.374892 | -0.321908 | 0.111747 |
| 7 | C | -3.499465 | 0.368017 | -0.129950 |
| 8 | C | -4.807182 | -0.277730 | -0.088982 |
| 9 | O | -4.875087 | -1.523581 | 0.182872 |
| 10 | C | 2.289076 | -0.296827 | 0.484791 |
| 11 | C | 3.587902 | 0.422640 | 0.499896 |
| 12 | C | 4.719799 | -0.323475 | -0.129922 |
| 13 | O | 5.857900 | 0.240918 | -0.075415 |
| 14 | O | -5.810128 | 0.474125 | -0.337536 |
| 15 | O | 4.477511 | -1.445900 | -0.675666 |
| 16 | H | 1.367701 | 2.464321 | -0.242002 |
| 17 | H | -1.308666 | 2.450238 | -0.445761 |
| 18 | H | -2.391169 | -1.384050 | 0.348212 |
| 19 | H | -3.516538 | 1.428476 | -0.367875 |
| 20 | H | 2.280057 | -1.360012 | 0.727724 |
| 21 | H | 3.881892 | 0.590747 | 1.549736 |
| 22 | H | -6.703461 | -0.004311 | -0.301377 |
| 23 | H | 5.292989 | -1.902300 | -1.078505 |
| 24 | H | 3.551282 | 1.414474 | 0.040047 |
| 25 | H | -5.818282 | -1.894202 | 0.189570 |
| 26 | H | 6.605089 | -0.284111 | -0.521283 |

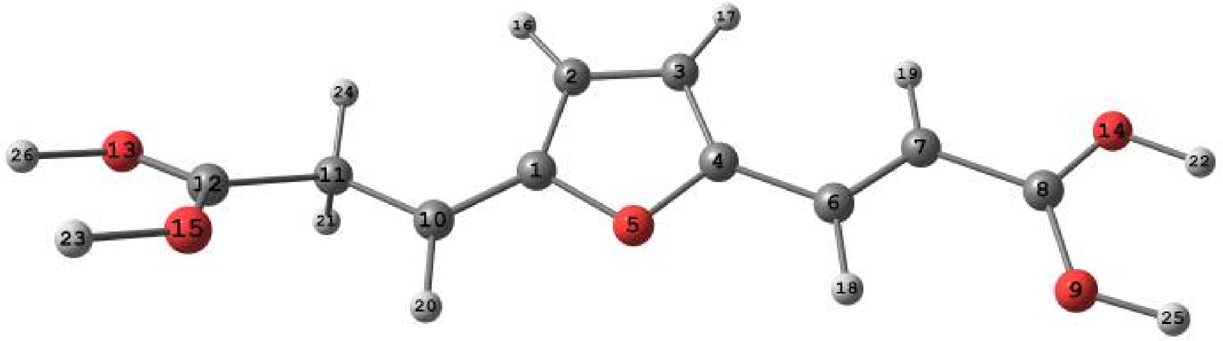

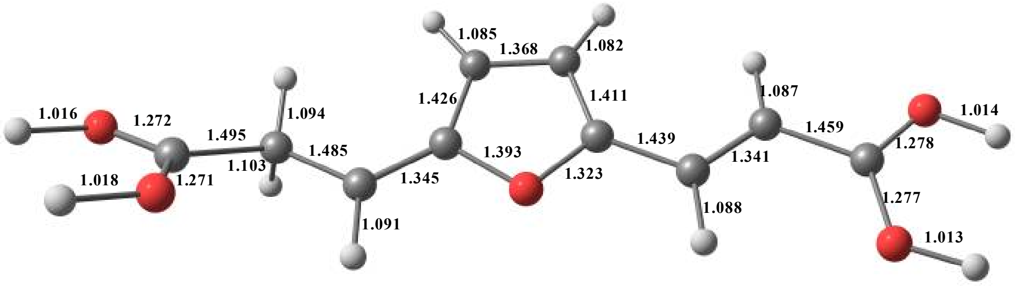


**Summary of Natural Population Analysis:**

| Atom | No | Natural Charge | Core | Valence | Rydberg | Total |
|---|---|---|---|---|---|---|
| C | 1 | 0.23870 | 1.99883 | 3.74064 | 0.02183 | 5.76130 |
| C | 2 | -0.07413 | 1.99906 | 4.05823 | 0.01684 | 6.07413 |
| C | 3 | -0.23245 | 1.99904 | 4.21401 | 0.01940 | 6.23245 |
| C | 4 | 0.46789 | 1.99905 | 3.50802 | 0.02505 | 5.53211 |
| O | 5 | -0.40839 | 1.99968 | 6.38306 | 0.02565 | 8.40839 |
| C | 6 | -0.18834 | 1.99912 | 4.15261 | 0.03662 | 6.18834 |
| C | 7 | -0.12249 | 1.99889 | 4.10473 | 0.01888 | 6.12249 |
| C | 8 | 0.88975 | 1.99946 | 3.08479 | 0.02601 | 5.11025 |
| O | 9 | -0.61050 | 1.99969 | 6.58203 | 0.02878 | 8.61050 |
| C | 10 | 0.01440 | 1.99902 | 3.96799 | 0.01859 | 5.98560 |
| C | 11 | -0.53408 | 1.99899 | 4.51681 | 0.01829 | 6.53408 |
| C | 12 | 0.93950 | 1.99945 | 3.02997 | 0.03107 | 5.06050 |
| O | 13 | -0.59527 | 1.99967 | 6.57061 | 0.02499 | 8.59527 |
| O | 14 | -0.60031 | 1.99970 | 6.57027 | 0.03034 | 8.60031 |
| O | 15 | -0.59851 | 1.99967 | 6.57394 | 0.02490 | 8.59851 |
| H | 16 | 0.28380 | 0.00000 | 0.71464 | 0.00156 | 0.71620 |
| H | 17 | 0.28582 | 0.00000 | 0.71240 | 0.00177 | 0.71418 |
| H | 18 | 0.28299 | 0.00000 | 0.71499 | 0.00202 | 0.71701 |
| H | 19 | 0.26570 | 0.00000 | 0.73038 | 0.00392 | 0.73430 |
| H | 20 | 0.28640 | 0.00000 | 0.71175 | 0.00185 | 0.71360 |
| H | 21 | 0.32146 | 0.00000 | 0.67643 | 0.00212 | 0.67854 |
| H | 22 | 0.59636 | 0.00000 | 0.40101 | 0.00263 | 0.40364 |
| H | 23 | 0.60334 | 0.00000 | 0.39414 | 0.00251 | 0.39666 |
| H | 24 | 0.29439 | 0.00000 | 0.70357 | 0.00204 | 0.70561 |
| H | 25 | 0.59849 | 0.00000 | 0.39893 | 0.00258 | 0.40151 |
| H | 26 | 0.59998 | 0.00000 | 0.39747 | 0.00256 | 0.40002 |
| * Total * | | 3.00450 | 29.98930 | 77.61341 | 0.39279 | 107.99550 |

**Ce**

**Energy E(B3LYP) = -763.81729201 h, $G^{298}$ = -763.659391 h, μ=2.87 D**

**Cartesian coordinates, Å**

| N | atom | x | y | z |
|---|---|---|---|---|
| 1 | C | -1.081903 | 0.375339 | -0.014139 |
| 2 | O | 0.000161 | -0.436368 | -0.018067 |
| 3 | C | 1.082119 | 0.375370 | -0.008656 |
| 4 | C | 0.694580 | 1.727420 | 0.001037 |
| 5 | C | -0.694484 | 1.727417 | -0.003458 |
| 6 | C | 2.325867 | -0.256397 | -0.004426 |
| 7 | C | 3.592628 | 0.462362 | 0.030214 |
| 8 | C | 4.834110 | -0.377242 | 0.006750 |
| 9 | O | 5.923035 | 0.270548 | 0.003560 |
| 10 | C | -2.325614 | -0.256376 | -0.016211 |
| 11 | C | -3.592532 | 0.462937 | -0.013602 |
| 12 | C | -4.833859 | -0.377025 | 0.004589 |
| 13 | O | -4.706307 | -1.636776 | 0.022221 |
| 14 | O | 4.705747 | -1.637025 | -0.009650 |
| 15 | O | -5.923238 | 0.270009 | 0.006044 |
| 16 | H | 1.351229 | 2.593793 | 0.009189 |
| 17 | H | -1.351180 | 2.593771 | 0.002233 |
| 18 | H | -2.301743 | -1.353670 | -0.024019 |
| 19 | H | -3.635029 | 1.170830 | 0.838050 |
| 20 | H | 2.302014 | -1.353639 | -0.019002 |
| 21 | H | 3.630984 | 1.111882 | 0.929664 |
| 22 | H | -6.760600 | -0.312401 | 0.022289 |
| 23 | H | 5.585911 | -2.155106 | -0.025158 |
| 24 | H | 3.648557 | 1.200102 | -0.794133 |
| 25 | H | -3.644934 | 1.144224 | -0.887719 |
| 26 | H | -5.586715 | -2.154506 | 0.037411 |
| 27 | H | 6.760860 | -0.311208 | -0.014265 |

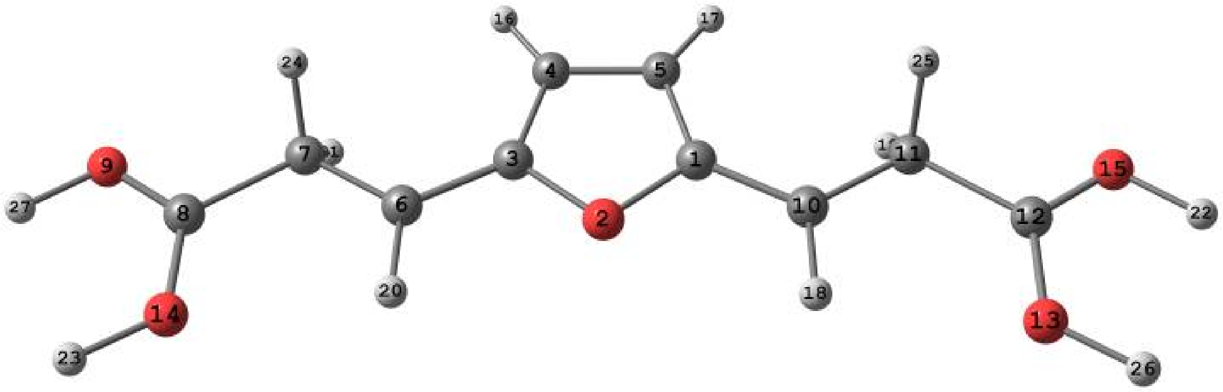

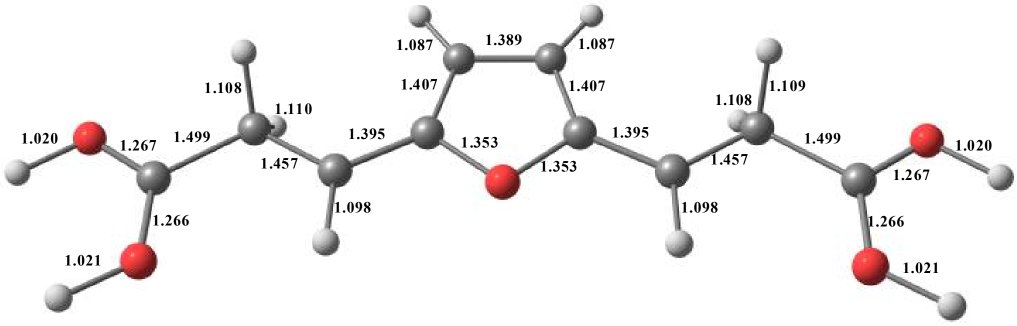


**Summary of Natural Population Analysis:**

| Atom | No | Natural Charge | Core | Valence | Rydberg | Total |
|---|---|---|---|---|---|---|
| C | 1 | 0.27550 | 1.99898 | 3.70228 | 0.02324 | 5.72450 |
| C | 2 | -0.07795 | 1.99907 | 4.05980 | 0.01907 | 6.07795 |
| C | 3 | -0.07590 | 1.99908 | 4.05967 | 0.01714 | 6.07590 |
| C | 4 | 0.28150 | 1.99897 | 3.69721 | 0.02232 | 5.71850 |
| O | 5 | -0.36135 | 1.99968 | 6.33659 | 0.02508 | 8.36135 |
| C | 6 | 0.24362 | 1.99911 | 3.73967 | 0.01760 | 5.75638 |
| C | 7 | -0.59595 | 1.99896 | 4.57936 | 0.01763 | 6.59595 |
| C | 8 | 0.93706 | 1.99947 | 3.03230 | 0.03116 | 5.06294 |
| O | 9 | -0.59175 | 1.99966 | 6.56694 | 0.02515 | 8.59175 |
| C | 10 | 0.22413 | 1.99902 | 3.74956 | 0.02729 | 5.77587 |
| C | 11 | -0.51537 | 1.99897 | 4.49152 | 0.02488 | 6.51537 |
| C | 12 | 0.91776 | 1.99954 | 3.04721 | 0.03548 | 5.08224 |
| O | 13 | -0.58753 | 1.99968 | 6.56242 | 0.02543 | 8.58753 |
| O | 14 | -0.58024 | 1.99966 | 6.55549 | 0.02509 | 8.58024 |
| O | 15 | -0.59849 | 1.99968 | 6.57422 | 0.02460 | 8.59849 |
| H | 16 | 0.30634 | 0.00000 | 0.69200 | 0.00167 | 0.69366 |
| H | 17 | 0.30658 | 0.00000 | 0.69188 | 0.00154 | 0.69342 |
| H | 18 | 0.30709 | 0.00000 | 0.69099 | 0.00192 | 0.69291 |
| H | 19 | 0.36111 | 0.00000 | 0.63686 | 0.00202 | 0.63889 |
| H | 20 | 0.30592 | 0.00000 | 0.69193 | 0.00215 | 0.69408 |
| H | 21 | 0.35715 | 0.00000 | 0.63771 | 0.00514 | 0.64285 |
| H | 22 | 0.60894 | 0.00000 | 0.38860 | 0.00246 | 0.39106 |
| H | 23 | 0.61488 | 0.00000 | 0.38251 | 0.00260 | 0.38512 |
| H | 24 | 0.35071 | 0.00000 | 0.64408 | 0.00521 | 0.64929 |
| H | 25 | 0.36467 | 0.00000 | 0.63331 | 0.00203 | 0.63533 |
| H | 26 | 0.61387 | 0.00000 | 0.38370 | 0.00243 | 0.38613 |
| H | 27 | 0.61002 | 0.00000 | 0.38740 | 0.00257 | 0.38998 |
| * Total * | | 4.00235 | 29.98953 | 77.61521 | 0.39291 | 107.99765 |

(Core, Valence, Rydberg and Total are grouped under "Natural Population".)

**Compound 1h**
**Energy E(B3LYP) = -840.887057444 h, $G^{298}$ = -840.714184 h, μ=3.23 D**

**Cartesian coordinates, Å**

| N | atom | x | y | z |
|---|---|---|---|---|
| 1 | C | 1.044988 | 0.528149 | -0.004258 |
| 2 | C | 0.717597 | 1.866966 | -0.005914 |
| 3 | C | -0.687499 | 1.945563 | -0.006260 |
| 4 | C | -1.161453 | 0.651275 | -0.004891 |
| 5 | O | -0.103315 | -0.215051 | -0.003918 |
| 6 | C | -2.464830 | 0.058256 | -0.003802 |
| 7 | C | -3.622943 | 0.744124 | 0.001726 |
| 8 | C | -4.948896 | 0.120361 | 0.005682 |
| 9 | O | -4.924814 | -1.224783 | -0.008712 |
| 10 | C | -6.204434 | -1.887626 | -0.004319 |
| 11 | C | 2.273582 | -0.205334 | -0.001944 |
| 12 | C | 3.503886 | 0.338092 | -0.001327 |
| 13 | C | 4.700258 | -0.510932 | 0.002189 |
| 14 | O | 5.822555 | 0.238359 | 0.001147 |
| 15 | C | 7.070977 | -0.481833 | 0.004612 |
| 16 | O | -5.987037 | 0.762277 | 0.021050 |
| 17 | O | 4.719256 | -1.728940 | 0.005774 |
| 18 | H | 1.415388 | 2.691522 | -0.006686 |
| 19 | H | -1.289190 | 2.842667 | -0.007463 |
| 20 | H | -2.481528 | -1.027490 | -0.006728 |
| 21 | H | -3.644170 | 1.828265 | 0.006616 |
| 22 | H | 2.175855 | -1.287990 | -0.000103 |
| 23 | H | 3.662324 | 1.410573 | -0.003210 |
| 24 | H | -6.762730 | -1.628292 | 0.892738 |
| 25 | H | -6.778882 | -1.608739 | -0.885159 |
| 26 | H | -5.978833 | -2.948906 | -0.018036 |
| 27 | H | 7.149707 | -1.106762 | -0.882503 |
| 28 | H | 7.843902 | 0.279657 | 0.003420 |
| 29 | H | 7.147600 | -1.101773 | 0.895412 |

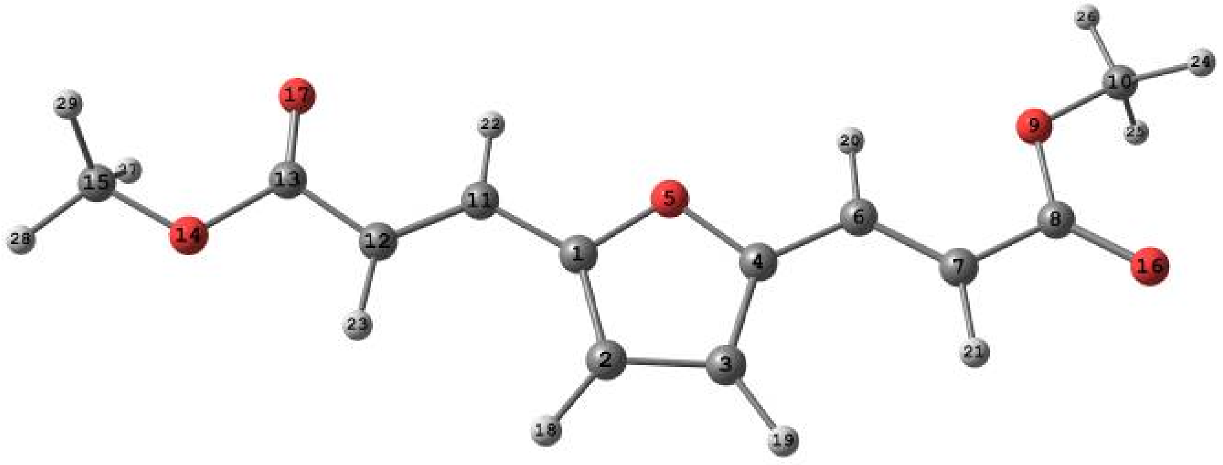

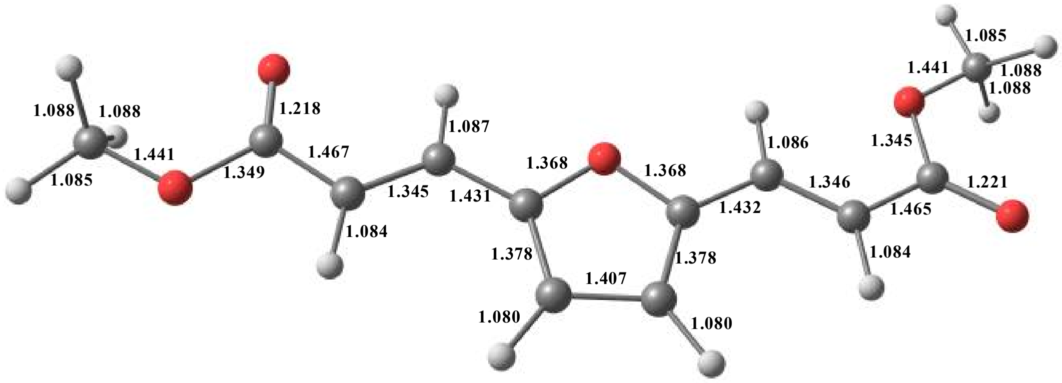


**Summary of Natural Population Analysis:**

| Atom | No | Natural Charge | Core | Valence | Rydberg | Total |
|---|---|---|---|---|---|---|
| C | 1 | 0.27042 | 1.99894 | 3.70700 | 0.02365 | 5.72958 |
| C | 2 | -0.24023 | 1.99904 | 4.22431 | 0.01689 | 6.24023 |
| C | 3 | -0.24163 | 1.99903 | 4.22557 | 0.01703 | 6.24163 |
| C | 4 | 0.26624 | 1.99895 | 3.71058 | 0.02422 | 5.73376 |
| O | 5 | -0.46002 | 1.99970 | 6.43608 | 0.02423 | 8.46002 |
| C | 6 | -0.16016 | 1.99884 | 4.13983 | 0.02148 | 6.16016 |
| C | 7 | -0.28237 | 1.99886 | 4.26196 | 0.02154 | 6.28237 |
| C | 8 | 0.78756 | 1.99932 | 3.17692 | 0.03620 | 5.21244 |
| O | 9 | -0.56207 | 1.99971 | 6.53767 | 0.02468 | 8.56207 |
| C | 10 | -0.21259 | 1.99923 | 4.19927 | 0.01409 | 6.21259 |
| C | 11 | -0.17053 | 1.99906 | 4.15009 | 0.02138 | 6.17053 |
| C | 12 | -0.28410 | 1.99883 | 4.26404 | 0.02123 | 6.28410 |
| C | 13 | 0.78555 | 1.99934 | 3.17464 | 0.04046 | 5.21445 |
| O | 14 | -0.54969 | 1.99973 | 6.53341 | 0.01655 | 8.54969 |
| C | 15 | -0.21529 | 1.99923 | 4.20153 | 0.01454 | 6.21529 |
| O | 16 | -0.66499 | 1.99975 | 6.63989 | 0.02534 | 8.66499 |
| O | 17 | -0.67119 | 1.99974 | 6.64567 | 0.02578 | 8.67119 |
| H | 18 | 0.25145 | 0.00000 | 0.74702 | 0.00153 | 0.74855 |
| H | 19 | 0.25164 | 0.00000 | 0.74682 | 0.00154 | 0.74836 |
| H | 20 | 0.24580 | 0.00000 | 0.75179 | 0.00242 | 0.75420 |
| H | 21 | 0.23210 | 0.00000 | 0.76569 | 0.00222 | 0.76790 |
| H | 22 | 0.24640 | 0.00000 | 0.75119 | 0.00241 | 0.75360 |
| H | 23 | 0.23348 | 0.00000 | 0.76435 | 0.00217 | 0.76652 |
| H | 24 | 0.18882 | 0.00000 | 0.80911 | 0.00207 | 0.81118 |
| H | 25 | 0.18887 | 0.00000 | 0.80907 | 0.00206 | 0.81113 |
| H | 26 | 0.19375 | 0.00000 | 0.80489 | 0.00136 | 0.80625 |

| | | | | | | |
|---|---|---|---|---|---|---|
| H | 27 | 0.18949 | 0.00000 | 0.80866 | 0.00185 | 0.81051 |
| H | 28 | 0.19385 | 0.00000 | 0.80484 | 0.00131 | 0.80615 |
| H | 29 | 0.18947 | 0.00000 | 0.80868 | 0.00185 | 0.81053 |
| * Total * | | 0.00003 | 33.98732 | 89.60056 | 0.41210 | 123.99997 |

**Ah**

**Energy E(B3LYP) = -841.708370748 h, $G^{298}$ = -841.514366 h, μ=2.87 D**

**Cartesian coordinates, Å**

| N | atom | x | y | z |
|---|---|---|---|---|
| 1 | C | -1.047205 | -0.508174 | -0.002621 |
| 2 | C | -0.713583 | -1.854029 | -0.001823 |
| 3 | C | 0.681643 | -1.922570 | -0.001179 |
| 4 | C | 1.145209 | -0.615515 | -0.001580 |
| 5 | O | 0.088518 | 0.243333 | -0.002669 |
| 6 | C | 2.436911 | -0.027275 | -0.001035 |
| 7 | C | 3.596068 | -0.731190 | 0.000819 |
| 8 | C | 4.868142 | -0.086432 | 0.001283 |
| 9 | O | 4.921753 | 1.202932 | -0.001347 |
| 10 | C | 6.173899 | 1.943872 | 0.001154 |
| 11 | C | -2.274370 | 0.204859 | -0.002991 |
| 12 | C | -3.499142 | -0.377984 | -0.001976 |
| 13 | C | -4.694929 | 0.397945 | -0.000879 |
| 14 | O | -5.802517 | -0.268350 | 0.002652 |
| 15 | C | -7.107521 | 0.374923 | 0.005534 |
| 16 | O | 5.899524 | -0.870637 | 0.004496 |
| 17 | O | -4.608966 | 1.688740 | -0.001709 |
| 18 | H | -1.408198 | -2.682566 | -0.001592 |
| 19 | H | 1.291883 | -2.815133 | -0.000376 |
| 20 | H | 2.458862 | 1.060274 | -0.002046 |
| 21 | H | 3.624823 | -1.815316 | 0.002168 |
| 22 | H | -2.186103 | 1.288867 | -0.003684 |
| 23 | H | -3.640393 | -1.453168 | -0.001000 |
| 24 | H | 6.738326 | 1.703230 | 0.900566 |
| 25 | H | 6.736786 | 1.710680 | -0.901164 |
| 26 | H | 5.875372 | 2.985031 | 0.005798 |
| 27 | H | -7.221403 | 0.972135 | -0.897710 |
| 28 | H | -7.816244 | -0.444083 | 0.012665 |
| 29 | H | -7.213547 | 0.980791 | 0.903982 |
| 30 | H | 6.807853 | -0.489036 | 0.003673 |
| 31 | H | -5.425234 | 2.239575 | -0.000890 |

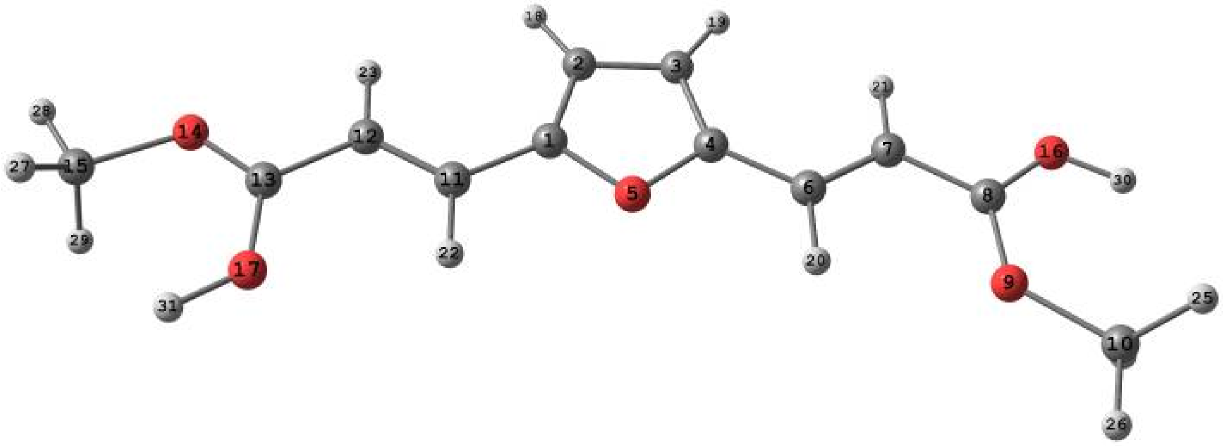

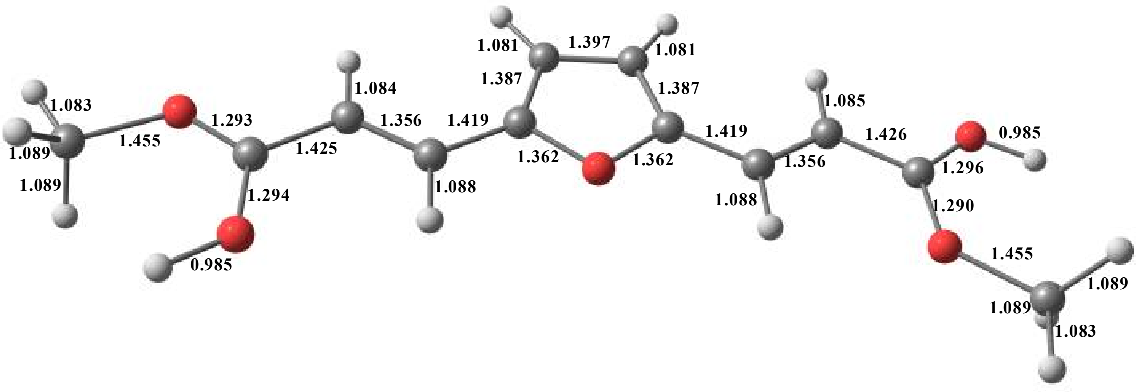


**Summary of Natural Population Analysis:**

| Atom | No | Natural Charge | Core | Valence | Rydberg | Total |
|---|---|---|---|---|---|---|
| C | 1 | 0.27696 | 1.99897 | 3.69928 | 0.02479 | 5.72304 |
| C | 2 | -0.20260 | 1.99904 | 4.18533 | 0.01823 | 6.20260 |
| C | 3 | -0.20252 | 1.99905 | 4.18514 | 0.01834 | 6.20252 |
| C | 4 | 0.28254 | 1.99896 | 3.69410 | 0.02440 | 5.71746 |
| O | 5 | -0.43612 | 1.99970 | 6.41153 | 0.02489 | 8.43612 |
| C | 6 | -0.13821 | 1.99914 | 4.09988 | 0.03919 | 6.13821 |
| C | 7 | -0.23160 | 1.99891 | 4.21012 | 0.02256 | 6.23160 |
| C | 8 | 0.88217 | 1.99927 | 3.09436 | 0.02420 | 5.11783 |
| O | 9 | -0.48621 | 1.99965 | 6.45730 | 0.02927 | 8.48621 |
| C | 10 | -0.23457 | 1.99921 | 4.21884 | 0.01653 | 6.23457 |
| C | 11 | -0.11701 | 1.99893 | 4.08628 | 0.03180 | 6.11701 |
| C | 12 | -0.22474 | 1.99887 | 4.20489 | 0.02098 | 6.22474 |
| C | 13 | 0.82089 | 1.99927 | 3.14549 | 0.03435 | 5.17911 |
| O | 14 | -0.46255 | 1.99965 | 6.43936 | 0.02355 | 8.46255 |
| C | 15 | -0.23368 | 1.99920 | 4.22161 | 0.01287 | 6.23368 |
| O | 16 | -0.63472 | 1.99970 | 6.60276 | 0.03227 | 8.63472 |
| O | 17 | -0.62854 | 1.99967 | 6.60558 | 0.02329 | 8.62854 |
| H | 18 | 0.26497 | 0.00000 | 0.73342 | 0.00161 | 0.73503 |
| H | 19 | 0.26482 | 0.00000 | 0.73349 | 0.00169 | 0.73518 |
| H | 20 | 0.26325 | 0.00000 | 0.73425 | 0.00250 | 0.73675 |
| H | 21 | 0.25252 | 0.00000 | 0.74315 | 0.00433 | 0.74748 |
| H | 22 | 0.26217 | 0.00000 | 0.73548 | 0.00235 | 0.73783 |
| H | 23 | 0.25430 | 0.00000 | 0.74157 | 0.00414 | 0.74570 |
| H | 24 | 0.20809 | 0.00000 | 0.78987 | 0.00204 | 0.79191 |
| H | 25 | 0.20838 | 0.00000 | 0.78959 | 0.00203 | 0.79162 |
| H | 26 | 0.22546 | 0.00000 | 0.77324 | 0.00129 | 0.77454 |
| H | 27 | 0.20908 | 0.00000 | 0.78921 | 0.00171 | 0.79092 |

| | | | | | | |
|---|---|---|---|---|---|---|
| H | 28 | 0.22549 | 0.00000 | 0.77335 | 0.00116 | 0.77451 |
| H | 29 | 0.20887 | 0.00000 | 0.78942 | 0.00172 | 0.79113 |
| H | 30 | 0.56425 | 0.00000 | 0.43302 | 0.00273 | 0.43575 |
| H | 31 | 0.56670 | 0.00000 | 0.43043 | 0.00286 | 0.43330 |

====================================================================

| | | | | | |
|---|---|---|---|---|---|
| * Total * | 2.00783 | 33.98720 | 89.55132 | 0.45365 | 123.99217 |

**Bh**

**Energy E(B3LYP) = -842.085368228 h, $G^{298}$ = -841.880944 h, μ=2.21 D**

**Cartesian coordinates, Å**

| N | atom | x | y | z |
|---|---|---|---|---|
| 1 | O | -0.131740 | -0.373248 | 0.348377 |
| 2 | C | 1.058216 | 0.350352 | 0.326147 |
| 3 | C | 0.736183 | 1.709420 | 0.038202 |
| 4 | C | -0.621620 | 1.783527 | -0.111175 |
| 5 | C | -1.113252 | 0.476798 | 0.092506 |
| 6 | C | 2.204066 | -0.311422 | 0.570542 |
| 7 | C | 3.528934 | 0.352185 | 0.666638 |
| 8 | C | 4.656527 | -0.379528 | 0.004242 |
| 9 | O | 4.399014 | -1.512326 | -0.515238 |
| 10 | C | -2.445248 | -0.066631 | 0.073639 |
| 11 | C | -3.517245 | 0.698334 | -0.181627 |
| 12 | C | -4.868964 | 0.145535 | -0.192333 |
| 13 | O | -5.790254 | 0.990948 | -0.464614 |
| 14 | O | -5.013071 | -1.093784 | 0.066096 |
| 15 | C | -6.311453 | -1.771890 | 0.101115 |
| 16 | O | 5.769584 | 0.227560 | 0.036575 |
| 17 | C | 7.015576 | -0.289618 | -0.545416 |
| 18 | H | 1.448564 | 2.522415 | -0.050591 |
| 19 | H | -1.214939 | 2.660018 | -0.338000 |
| 20 | H | -2.532794 | -1.131850 | 0.278549 |
| 21 | H | -3.459153 | 1.762979 | -0.393537 |
| 22 | H | 2.140736 | -1.383743 | 0.758405 |
| 23 | H | 3.805358 | 0.414451 | 1.733803 |
| 24 | H | -6.922188 | -1.320111 | 0.880041 |
| 25 | H | -6.776269 | -1.694187 | -0.879687 |
| 26 | H | -6.069351 | -2.799824 | 0.339410 |
| 27 | H | 6.867823 | -0.435946 | -1.612982 |
| 28 | H | 7.740723 | 0.490084 | -0.350526 |
| 29 | H | 7.280391 | -1.213634 | -0.036659 |
| 30 | H | -6.748163 | 0.725915 | -0.499628 |
| 31 | H | 5.102362 | -2.072193 | -0.949626 |
| 32 | H | 3.538317 | 1.380035 | 0.296569 |

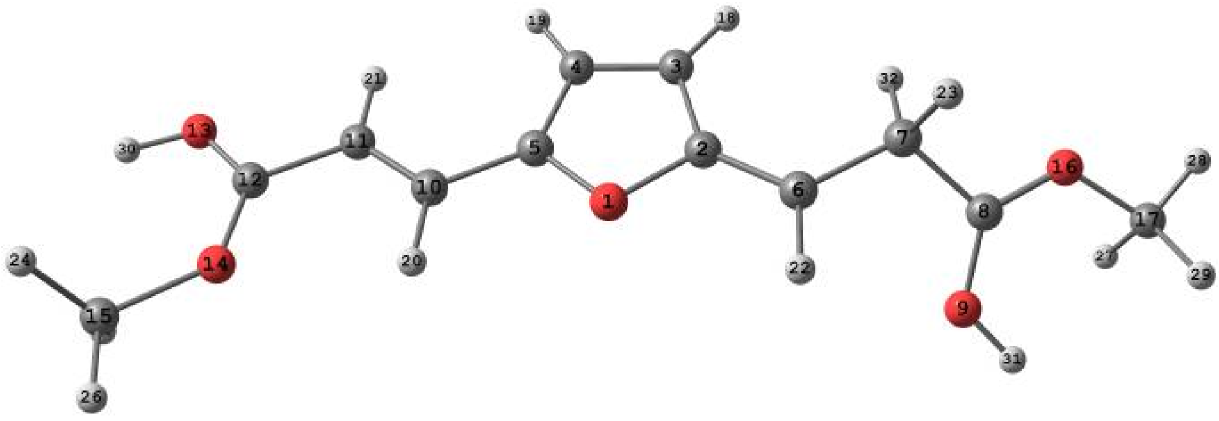

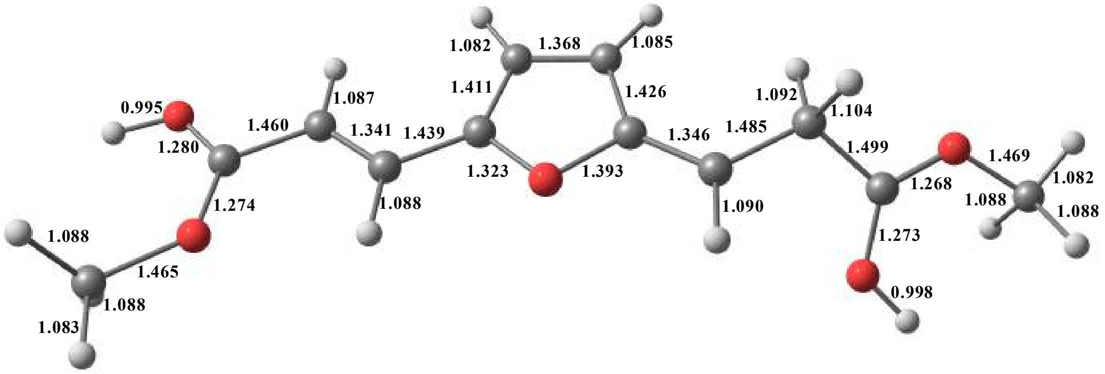


**Summary of Natural Population Analysis:**

| Atom | No | Natural Charge | Core | Valence | Rydberg | Total |
|---|---|---|---|---|---|---|
| O | 1 | -0.40868 | 1.99968 | 6.38309 | 0.02591 | 8.40868 |
| C | 2 | 0.23907 | 1.99883 | 3.73960 | 0.02250 | 5.76093 |
| C | 3 | -0.07432 | 1.99906 | 4.05844 | 0.01682 | 6.07432 |
| C | 4 | -0.23291 | 1.99903 | 4.21412 | 0.01976 | 6.23291 |
| C | 5 | 0.46238 | 1.99905 | 3.51286 | 0.02571 | 5.53762 |
| C | 6 | 0.02546 | 1.99899 | 3.95762 | 0.01794 | 5.97454 |
| C | 7 | -0.53801 | 1.99912 | 4.51913 | 0.01977 | 6.53801 |
| C | 8 | 0.95046 | 1.99934 | 3.02058 | 0.02961 | 5.04954 |
| O | 9 | -0.59927 | 1.99965 | 6.57583 | 0.02380 | 8.59927 |
| C | 10 | -0.17662 | 1.99890 | 4.13833 | 0.03938 | 6.17662 |
| C | 11 | -0.11951 | 1.99891 | 4.09790 | 0.02270 | 6.11951 |
| C | 12 | 0.91349 | 1.99936 | 3.06273 | 0.02443 | 5.08651 |
| O | 13 | -0.60614 | 1.99968 | 6.57446 | 0.03199 | 8.60614 |
| O | 14 | -0.45679 | 1.99964 | 6.42857 | 0.02858 | 8.45679 |
| C | 15 | -0.23113 | 1.99921 | 4.21382 | 0.01809 | 6.23113 |
| O | 16 | -0.44175 | 1.99961 | 6.41919 | 0.02294 | 8.44175 |
| C | 17 | -0.22682 | 1.99920 | 4.21366 | 0.01396 | 6.22682 |
| H | 18 | 0.28380 | 0.00000 | 0.71458 | 0.00162 | 0.71620 |
| H | 19 | 0.28579 | 0.00000 | 0.71236 | 0.00185 | 0.71421 |
| H | 20 | 0.28171 | 0.00000 | 0.71569 | 0.00260 | 0.71829 |
| H | 21 | 0.26709 | 0.00000 | 0.72898 | 0.00392 | 0.73291 |
| H | 22 | 0.28223 | 0.00000 | 0.71578 | 0.00199 | 0.71777 |
| H | 23 | 0.32485 | 0.00000 | 0.67331 | 0.00185 | 0.67515 |
| H | 24 | 0.21687 | 0.00000 | 0.78141 | 0.00172 | 0.78313 |
| H | 25 | 0.21636 | 0.00000 | 0.78191 | 0.00173 | 0.78364 |
| H | 26 | 0.23071 | 0.00000 | 0.76816 | 0.00113 | 0.76929 |

| | | | | | | |
|---|---|---|---|---|---|---|
| H | 27 | 0.21749 | 0.00000 | 0.78120 | 0.00131 | 0.78251 |
| H | 28 | 0.23101 | 0.00000 | 0.76800 | 0.00099 | 0.76899 |
| H | 29 | 0.21803 | 0.00000 | 0.78068 | 0.00129 | 0.78197 |
| H | 30 | 0.58465 | 0.00000 | 0.41270 | 0.00264 | 0.41535 |
| H | 31 | 0.59267 | 0.00000 | 0.40485 | 0.00249 | 0.40733 |
| H | 32 | 0.29285 | 0.00000 | 0.70514 | 0.00201 | 0.70715 |
| * Total * | | 3.00504 | 33.98725 | 89.57470 | 0.43302 | 123.99496 |

**Ch**

**Energy E(B3LYP) = -842.399291665 h, $G^{298}$ = -842.191548 h, μ=1.29 D**

**Cartesian coordinates, Å**

| N | atom | x | y | z |
|---|---|---|---|---|
| 1 | C | -1.018300 | -0.482640 | 0.081840 |
| 2 | C | -0.697009 | -1.850369 | 0.146243 |
| 3 | C | 0.688916 | -1.921047 | 0.087236 |
| 4 | C | 1.140716 | -0.592729 | -0.011412 |
| 5 | O | 0.100688 | 0.272223 | -0.011443 |
| 6 | C | 2.412655 | -0.028637 | -0.105622 |
| 7 | C | 3.634835 | -0.816018 | -0.189331 |
| 8 | C | 4.932916 | -0.063967 | -0.104612 |
| 9 | O | 4.876088 | 1.171857 | 0.153470 |
| 10 | C | 6.057429 | 2.047915 | 0.291030 |
| 11 | C | -2.228993 | 0.209962 | 0.085263 |
| 12 | C | -3.525197 | -0.436904 | 0.233037 |
| 13 | C | -4.734736 | 0.420632 | -0.014021 |
| 14 | O | -5.831310 | -0.196244 | 0.110303 |
| 15 | C | -7.159337 | 0.414406 | -0.078579 |
| 16 | O | 5.952873 | -0.791518 | -0.298511 |
| 17 | O | -4.530580 | 1.633832 | -0.314614 |
| 18 | H | -1.393835 | -2.681335 | 0.221531 |
| 19 | H | 1.302530 | -2.818159 | 0.114517 |
| 20 | H | 2.448543 | 1.068035 | -0.145461 |
| 21 | H | 3.655362 | -1.633227 | 0.554457 |
| 22 | H | -2.154716 | 1.301276 | -0.005819 |
| 23 | H | -3.592738 | -0.816905 | 1.278630 |
| 24 | H | 6.649183 | 1.690501 | 1.130184 |
| 25 | H | 6.609007 | 2.029550 | -0.645881 |
| 26 | H | 5.634281 | 3.024809 | 0.486624 |
| 27 | H | -7.230538 | 0.773551 | -1.102613 |
| 28 | H | -7.848532 | -0.399258 | 0.107388 |
| 29 | H | -7.276622 | 1.213490 | 0.649693 |
| 30 | H | 6.889560 | -0.437275 | -0.262946 |
| 31 | H | -5.268814 | 2.288983 | -0.500904 |
| 32 | H | -3.610443 | -1.359829 | -0.364941 |
| 33 | H | 3.622322 | -1.369024 | -1.154539 |

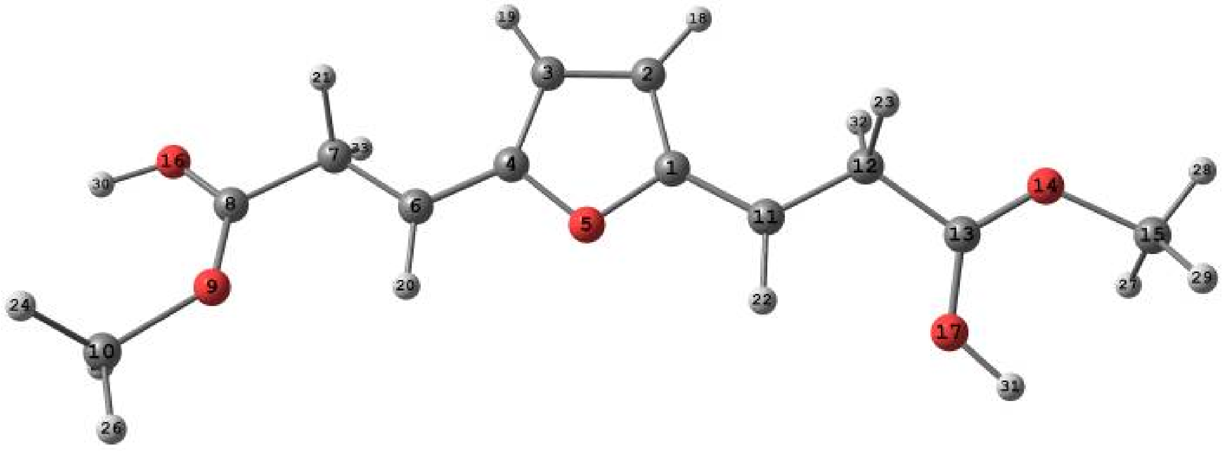

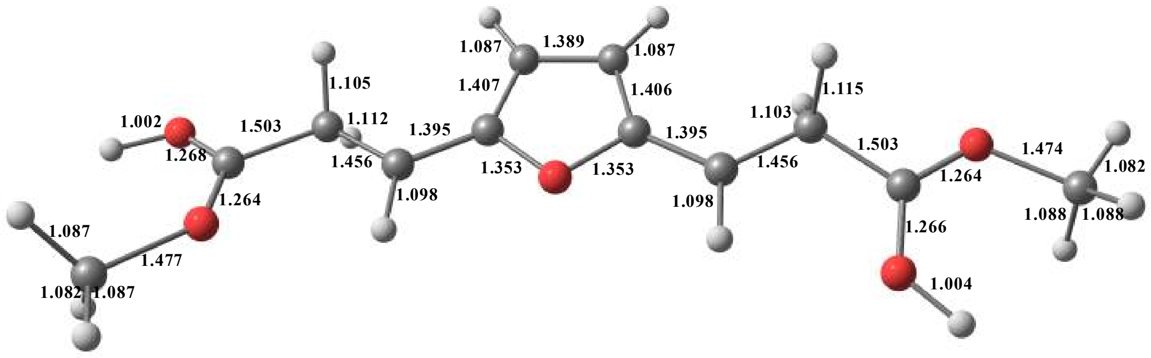


**Summary of Natural Population Analysis:**

| Atom | No | Natural Charge | Natural Population: Core | Natural Population: Valence | Natural Population: Rydberg | Natural Population: Total |
|---|---|---|---|---|---|---|
| C | 1 | 0.28252 | 1.99897 | 3.69674 | 0.02178 | 5.71748 |
| C | 2 | -0.07538 | 1.99908 | 4.05935 | 0.01695 | 6.07538 |
| C | 3 | -0.07544 | 1.99907 | 4.05928 | 0.01709 | 6.07544 |
| C | 4 | 0.27702 | 1.99897 | 3.70183 | 0.02217 | 5.72298 |
| O | 5 | -0.36252 | 1.99968 | 6.33664 | 0.02620 | 8.36252 |
| C | 6 | 0.24529 | 1.99894 | 3.73509 | 0.02068 | 5.75471 |
| C | 7 | -0.59013 | 1.99896 | 4.57306 | 0.01811 | 6.59013 |
| C | 8 | 0.94875 | 1.99937 | 3.02327 | 0.02862 | 5.05125 |
| O | 9 | -0.44768 | 1.99961 | 6.41894 | 0.02913 | 8.44768 |
| C | 10 | -0.20216 | 1.99919 | 4.18644 | 0.01652 | 6.20216 |
| C | 11 | 0.23516 | 1.99912 | 3.74843 | 0.01728 | 5.76484 |
| C | 12 | -0.58626 | 1.99896 | 4.56891 | 0.01839 | 6.58626 |
| C | 13 | 0.94761 | 1.99937 | 3.02381 | 0.02922 | 5.05239 |
| O | 14 | -0.42905 | 1.99961 | 6.40706 | 0.02238 | 8.42905 |
| C | 15 | -0.22384 | 1.99920 | 4.21095 | 0.01369 | 6.22384 |
| O | 16 | -0.58555 | 1.99964 | 6.56228 | 0.02363 | 8.58555 |
| O | 17 | -0.59691 | 1.99964 | 6.57331 | 0.02397 | 8.59691 |
| H | 18 | 0.30661 | 0.00000 | 0.69185 | 0.00153 | 0.69339 |
| H | 19 | 0.30681 | 0.00000 | 0.69165 | 0.00154 | 0.69319 |
| H | 20 | 0.31461 | 0.00000 | 0.68333 | 0.00206 | 0.68539 |
| H | 21 | 0.35176 | 0.00000 | 0.64603 | 0.00221 | 0.64824 |
| H | 22 | 0.31488 | 0.00000 | 0.68304 | 0.00208 | 0.68512 |
| H | 23 | 0.37642 | 0.00000 | 0.62137 | 0.00222 | 0.62358 |
| H | 24 | 0.21877 | 0.00000 | 0.77984 | 0.00138 | 0.78123 |
| H | 25 | 0.21776 | 0.00000 | 0.78084 | 0.00141 | 0.78224 |

| | | | | | | |
|---|---|---|---|---|---|---|
| H | 26 | 0.23001 | 0.00000 | 0.76880 | 0.00119 | 0.76999 |
| H | 27 | 0.22134 | 0.00000 | 0.77740 | 0.00126 | 0.77866 |
| H | 28 | 0.23339 | 0.00000 | 0.76563 | 0.00098 | 0.76661 |
| H | 29 | 0.22133 | 0.00000 | 0.77741 | 0.00126 | 0.77867 |
| H | 30 | 0.60080 | 0.00000 | 0.39666 | 0.00254 | 0.39920 |
| H | 31 | 0.60685 | 0.00000 | 0.39076 | 0.00239 | 0.39315 |
| H | 32 | 0.34734 | 0.00000 | 0.65046 | 0.00220 | 0.65266 |
| H | 33 | 0.37076 | 0.00000 | 0.62687 | 0.00237 | 0.62924 |
| * Total * | | 4.00087 | 33.98739 | 89.61732 | 0.39442 | 123.99913 |
